# Mechanistic insights into JSS1_004-mediated antagonism of the DndBCDE-FGH restriction system and engineering applications

**DOI:** 10.1128/mbio.01386-25

**Published:** 2025-07-14

**Authors:** Yu He, Susu Jiang, Fang Wang, Junwu Bi, Siting Li, Fuliang Yin, Shi Chen, Chao Chen, Lianrong Wang

**Affiliations:** 1 Department of Gastroenterology, Ministry of Education Key Laboratory of Combinatorial Biosynthesis and Drug Discovery, Hubei Clinical Center and Key Laboratory of Intestinal and Colorectal Disease, Zhongnan Hospital of Wuhan University, School of Pharmaceutical Sciences, Wuhan University, Wuhan, Hubei, China; 2 Department of Respiratory Diseases, Institute of Pediatrics, Shenzhen Children's Hospital, Shenzhen, China; 3 Department of Burn and Plastic Surgery, Shenzhen Key Laboratory of Microbiology in Genomic Modification & Editing and Application, Shenzhen Institute of Translational Medicine, Shenzhen University Medical School, Medical Innovation Technology Transformation Center of Shenzhen Second People’s Hospital, The First Affiliated Hospital of Shenzhen University, Shenzhen, China; National Institutes of Health, Bethesda, Maryland, USA

**Keywords:** DNA phosphorothioation, shutoff, antagonism

## Abstract

The bacterial DNA phosphorothioate (PT) modification system is orchestrated by the DndCDE enzymatic complex. This system collaborates with restriction components DndF, DndG, and DndH to establish a widespread prokaryotic anti-phage defense network. In the evolutionary arms race, phages such as JSS1 have evolved counter strategies, including the expression of the JSS1_004 protein, to subvert PT-mediated host immunity. Although the N-terminal kinase domain of JSS1_004 is known to inhibit DndFGH through phosphorylation-dependent inactivation, the functional role of its C-terminal shutoff domain remained enigmatic. Here, we demonstrate that both domains are indispensable for full antagonistic activity against the DndBCDE-FGH system. Genetic dissection revealed that the absence of either domain substantially compromises suppression efficacy. Notably, JSS1_004 exhibits pleiotropic effects on host physiology, including repressing the *dndBCDE-FGH* operon confirmed by transcriptome. ChIP-seq analysis further revealed that JSS1_004 binding to promoter regions and coding sequences of genes associated with protein synthesis machinery, including tRNA, rRNA, and ribosomal subunit genes (SSU/LSU), thereby hijacking the host’s translational apparatus. Although the potent inhibitory efficacy and broad-spectrum antagonistic properties make JSS1_004 a promising functional element for engineered phage applications, the severe cytotoxicity of JSS1_004 constrains its biotechnological applicability. To overcome this limitation, we engineered attenuated JSS1_004 variants. These modified constructs maintained robust antagonism against the DndBCDE-FGH system while significantly reducing host toxicity, enabling cross-protection of heterologous phages against PT-based restriction and expanding the potential of phage therapy. Our findings advance the understanding of phage-host interactions and provide a framework for optimizing phage therapeutics to circumvent bacterial defense barriers.

**IMPORTANCE:**

Our recent investigation elucidated the molecular mechanism by which bacteriophage JSS1 counteracted the bacterial DndBCDE-FGH anti-phage defense system. Upon host invasion, the early gene transcriptional fragment of JSS1 facilitated the rapid expression of JSS1_004, with a N-terminal kinase domain that mediated phosphorylation of serine, threonine, and tyrosine residues within the DndFGH defense complex. This post-translational modification induced conformational changes in the complex, effectively neutralizing its restriction activity against phage propagation. However, the role of the C-terminal shutoff domain remains to be elucidated. In this study, we revealed that both the kinase and shutoff domains were crucial for the antagonistic function of JSS1_004. Moreover, transcriptomic and ChIP-seq analyses revealed JSS1_004’s broad expressional regulation of host gene expression, thus establishing a cellular environment conducive to bacteriophage replication. Furthermore, we successfully developed and integrated attenuated-cytotoxicity variants of JSS1_004 into the genome of M13 phages, conferring robust resistance against the DndBCDE-FGH defense system. These findings provide critical insights into the molecular arms race between phages and prokaryotic hosts while expanding the toolkit for developing phage-based biotechnological applications.

## INTRODUCTION

To counteract the threat posed by phages or viruses, microorganisms have developed a diverse array of defense mechanisms targeting the whole stages of the viral life cycle. From the initial absorption of phages to the eventual burst of viral particles, microorganisms employ multiple strategies to protect themselves. These include receptor modification to prevent phage attachment, using receptors as decoys to lure in phages, non-self DNA degradation systems such as restriction-modification (R-M) systems, R-M-like systems (e.g., PT-related defense systems), or CRISPR-Cas systems specifically target viral genome to disrupt its replication and abortive infection (Abi) systems that sacrifice the infected host to prevent the spread of the invader, thereby safeguarding the larger population, such as Thoeris, Pycsar, Kongming, and Gabija anti-phage system (1–19). To combat these highly equipped bacteria, phages have evolved a variety of countermeasures to breach the bacterial defense systems. Specifically, Ocr (overcome classical restriction) proteins encoded by *gp0.3* of T7 phages can neutralize type I R-M and BREX systems by mimicking DNA structures. Bacillus phage Phi3T encoded the Gad1 protein that is directly bound to the Gabija complex (GajAB), inhibiting its ATPase-driven DNA cleavage activity and enabling escape from Gabija restriction (20–22). Moreover, some antagonistic strategies target the cofactor or signaling molecules of the defense barrier. For instance, S-adenosyl-L-methionine (SAM) hydrolase and Acb (anti-CBASS) proteins degrade SAM or cyclic nucleotides, thereby silencing R-M systems, BREX systems, or CBASS systems (23–25). Phage T5 evaded the Kongming defense system by secreting Dmp protein, which enzymatically degraded dAMP to disrupt nucleotide-mediated immunity. Similarly, phage SBSphiJ7 produced Tad1, an anti-Thoeris effector that sequestered signaling molecules generated by the TIR domain of the Thoeris system, thereby blocking NAD+ depletion and abortive infection (17, 26). Intriguingly, some phages have evolved a broad-spectrum antagonistic mechanism to overcome bacterial defenses. A notable example is the formation of a proteinaceous nucleus-like compartment by jumbo phages within infected cells to shield viral DNA from multiple CRISPR-Cas systems, including type I and II-A and R-M systems (27, 28).

Recently, we have uncovered a novel antagonistic mechanism mediated by JSS1_004, carrying a protein kinase domain in the N terminus (29). This versatile protein kinase, found almost exclusively in *Autographiviridae* phages, exhibits promiscuous activity, phosphorylating thousands of bacterial proteins that participate in diverse cellular metabolic pathways. Notably, JSS1_004 also targets key bacterial defense proteins, including DndFGH, CasD, HerA, and QatABCD, which are components of PT-dependent R-M-like system, the type I-E CRISPR-Cas adaptive immune system, SIR2 + HerA, DUF4297 + HerA Abi systems, and QatABCD system with an unknown mechanism. Phosphorylation of these defense proteins by JSS1_004 disrupts their anti-phage activities, thereby rendering them ineffective.

However, JSS1_004 is a bifunctional protein containing both an N-terminal protein kinase domain and a C-terminal domain responsible for host transcriptional shutoff. Although the C-terminal domain exhibits high toxicity and is proposed to bind DNA via a strongly basic amino acid segment, recent studies of its homologous proteins have provided evidence for nucleic acid-binding activity, which represents significant progress in understanding the molecular mechanism of this unique shutoff domain (30). In this study, we elucidated the molecular mechanism by which the C-terminal domain of JSS1_004 confers antagonistic activity against the DndFGH host defense system, enabling phages to overcome this bacterial restriction mechanism. Notably, transcriptomic and ChIP-seq analyses revealed JSS1_004’s broad expressional regulation of host gene expression, thus establishing a cellular environment conducive to bacteriophage replication. Moreover, we developed attenuated-cytotoxicity JSS1_004 variants that maintain potent anti-DndFGH activity, serving as modular counter-defense elements. These engineered variants demonstrate remarkable versatility in phage defense evasion, enabling filamentous phages to circumvent the host DndBCDE-FGH defense system. This proof-of-concept advancement significantly expands the biotechnological utility of JSS1_004-derived components for phage engineering applications.

## RESULTS

### Both the kinase domain and shutoff domain of JSS1_004 contribute to the antagonism phenotype

**Fig 1:**
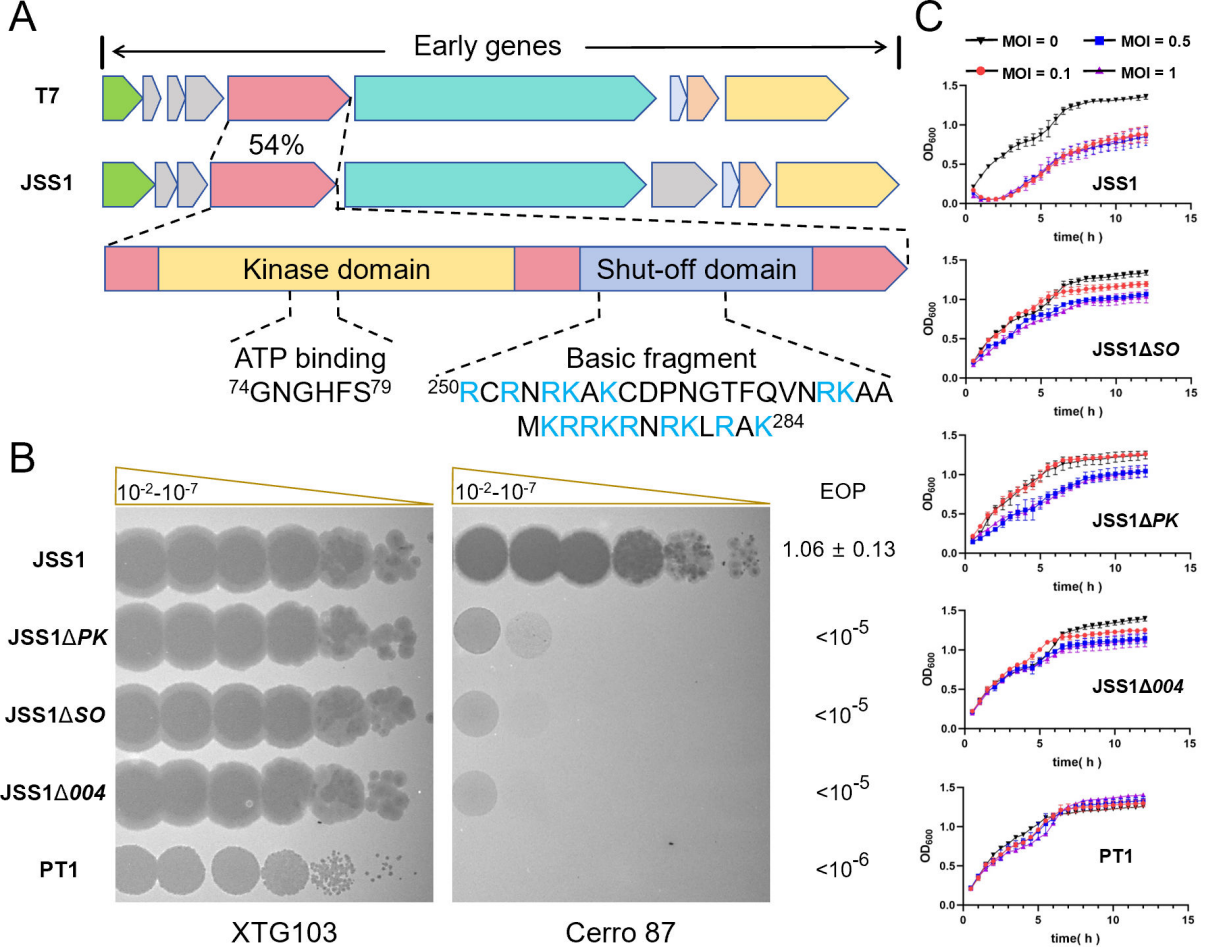
The kinase domain and transcriptional suppression module of JSS1_004 function cooperatively to antagonize the DndFGH bacterial defense machinery. (**A**) Comparative genomic organization of early-phase genes in T7-like phage JSS1 and T7. Homologous genes were color-matched, with *JSS1_004* (encoding a DndBCDE-FGH system antagonist) exhibiting 54% amino acid similarity to T7 *gp0.7*. Domain architectures of JSS1_004 were shown below, highlighting the N-terminal kinase domain and C-terminal shutoff domain whose basic peptide motifs implicated in nucleic acid interactions were mapped, with positively charged residues highlighted in blue. (**B**) Plaque formation efficiency of JSS1 and control phages on wild-type Cerro 87 and its restriction-deficient mutant XTG103. Phage suspensions were serially diluted and spotted using double-layer agar assays. EOP was defined as the ratio of plaque formation efficiency of the phage in the strains of Cerro 87 and XTG103. (**C**) Growth kinetics of Cerro 87 cultures treated with phages at MOIs of 0, 0.1, 0.5, and 1.

Recently, we identified critical phosphorylation hotspots within the DndFGH complex targeted by the N terminus of JSS1_004 through phosphoproteomic analysis. Phosphorylation at serine, threonine, and tyrosine residues induces conformational alterations in the DndFGH complex, compromising its antiphage activity (29). Although the N-terminal kinase domain of JSS1_004 mediates this phosphoregulation, the functional contribution of its C-terminal shutoff domain remains undefined. *JSS1_004* was an early gene of phage JSS1, which was a T7-like phage exhibiting triphasic gene expression (early, middle, and late phases), showing 54% homology to phage T7 *gp0.7* gene (Fig. 1A). Domain architecture analysis reveals two conserved regions: an N-terminal serine/threonine/tyrosine kinase domain and a C-terminal basic amino acid-enriched transcriptional repression domain (Fig. 1A). Previous studies demonstrated that JSS1_004 enables viral evasion of DndBCDE-DndFGH restriction, as evidenced by 10^6^-fold higher survival of wild-type JSS1 compared with JSS1Δ*004,* a *JSS1_004* gene-deficient JSS1 phage mutant, against Cerro 87 (29). To dissect domain-specific contributions, we engineered two truncation mutants: JSS1Δ*PK* (kinase domain deletion) and JSS1Δ*SO* (shutoff domain deletion) (Fig. S1). The direct observation was that both JSS1Δ*PK* and JSS1Δ*SO* abolished the antagonistic effect against DndFGH. Consistent with previous studies, although DndFGH in Cerro 87 could provide strong protection against JSS1Δ*004*, JSS1 remained completely insensitive to this defense barrier, as evidenced by similar efficiencies of plating (EOP) on Cerro 87 and its *dndBCDE-FGH*-deficient derivative XTG103. However, similar to JSS1Δ*004*, JSS1Δ*PK,* and JSS1Δ*SO* mutants also lost the antagonistic effect against DndFGH and exhibited comparable susceptibility, suggesting that both kinase domain and shutoff domain of JSS1_004 were indispensable to the antagonism phenotype (Fig. 1B). A similar trend was observed under liquid culture conditions. Bacterial growth was monitored by measuring OD_600_ after incubating Cerro 87 with phages at varying multiplicities of infection (MOIs). In sharp contrast to JSS1, which caused dramatic culture collapse in Cerro 87 even at an MOI of 0.1 (Fig. 1C), PT1, which was a lytic Cerro 87 phage belonging to *Myoviridae* members, and JSS1Δ*004* exhibited limited inhibition for the bacterial growth. The strong selection pressure posed by JSS1 led to the emergence of the phage-resistance escaper and the regrowth populations (Fig. S2). Notably, JSS1Δ*PK* and JSS1Δ*SO* mutants demonstrated intrinsic, although suboptimal, defense-countering activity, as evidenced by their reduced bacterial growth rates compared with Cerro 87 infected with JSS1Δ*004* (Fig. 1C). Collectively, these data established that both the kinase and shutoff domains were essential for full anti-restriction activity of JSS1_004.

### Suppression of DndBCDE-FGH antiviral system and host physiology optimization by JSS1_004

**Fig 2:**
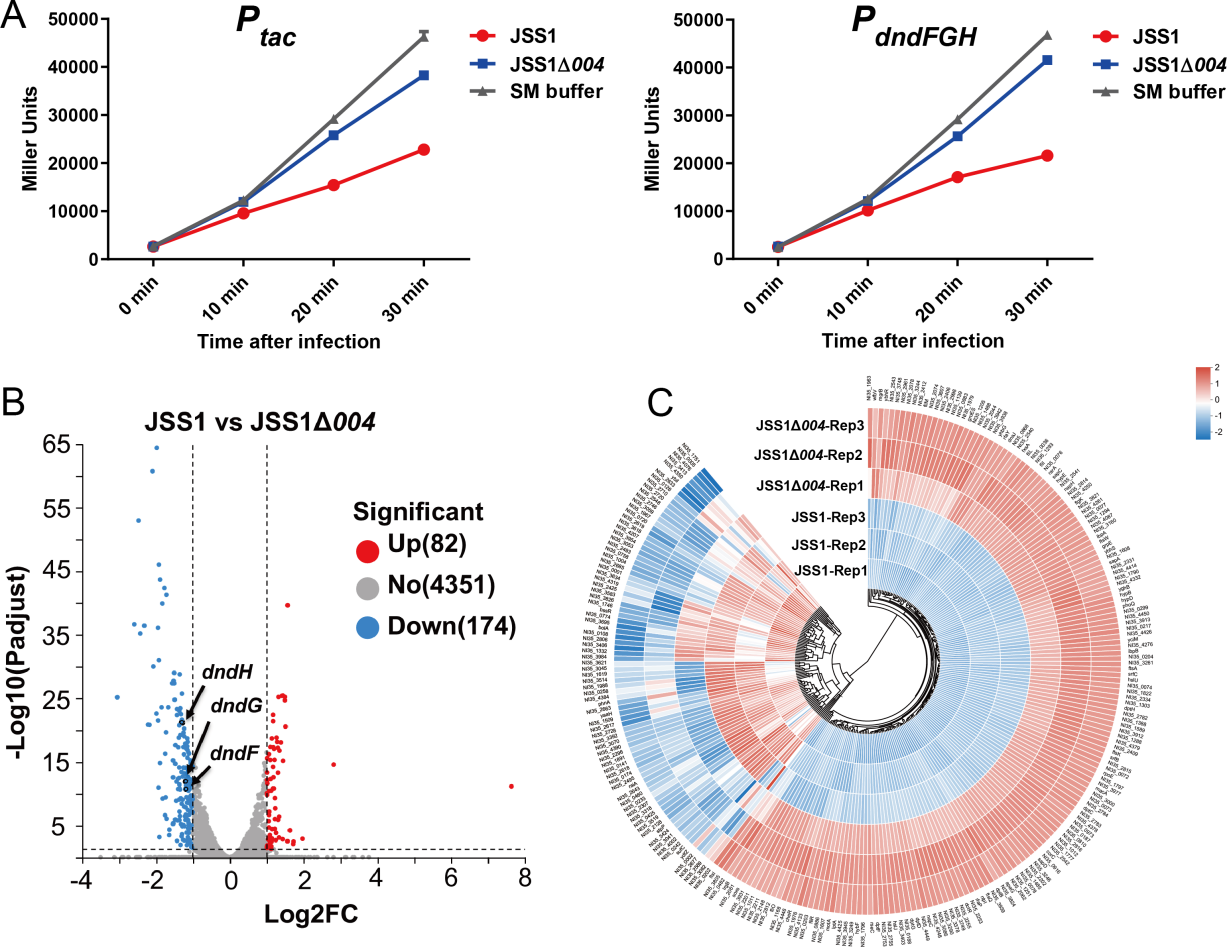
JSS1_004 regulated the transcription of host genes. (**A**) Induction of β-galactosidase activity (reported in Miller units) in Cerro 87 cells, in which the expression of a *lacZ* reporter cassette was under the control of native *P_dndFGH_* promoter (right) or *P_tac_* promoter (left) during phage infection. Cerro 87 cells were incubated at 28°C until the OD_600_ reached 0.4. Then, 1 mM IPTG was added to initiate the transcription of *lacZ*. After induction for 15 s, phages were added at an MOI of 5, and aliquots of the mixture were withdrawn at the indicated time points to perform the β-galactosidase assay. Data represent the mean ± SD from three independent experiments. (**B**) Volcano plot analysis of transcriptional changes induced by JSS1_004. Genes with absolute log_2_ fold change (log_2_FC) >1 were highlighted in red (upregulated) or blue (downregulated), whereas genes within the *dndFGH* cluster are marked with black circles. (**C**) Hierarchical clustering of differentially expressed genes from panel **B**. Color intensity reflects normalized expression levels, demonstrating consistent expression patterns across biological replicates within experimental groups. Heatmap rows represent genes, and columns represent samples (JSS1-infected vs. JSS1Δ*004*-infected).

To examine the potential repression of JSS1_004 on *dndBCDE-FGH* immunity, we first measured the expression level of the *dndFGH* operon in phage-infected cells via β-galactosidase assays. Apparent repression of β-galactosidase synthesis, driven by either the native *P_dndFGH_* promoter or the *P_tac_* promoter, was observed in phage JSS1-infected Cerro 87 cells (Fig. 2A). In contrast, cells challenged with phage JSS1Δ*004* showed a negligible decrease in their ability to synthesize β-galactosidase (Fig. 2A). The results confirmed that JSS1_004 helped suppress the host RNAP-catalyzed transcription of *dndFGH* during phage infection. To further validate these findings and investigate the transcriptional regulatory pattern of JSS1_004, we performed transcriptome profiling. Cerro 87 strains were infected with JSS1 or JSS1Δ*004* at an MOI of 10. Then, the infected bacteria were collected and subjected to transcriptome sequencing analysis. Analysis of RNA-seq data confirmed that JSS1_004-mediated transcriptional repression of both the *dndFGH* and *dndBCDE* gene clusters. Among the 4,450 host genes analyzed, JSS1_004 expression induced significant downregulation of 174 genes and upregulation of 82 genes (Fig. 2B; Data S1). The significantly differentially expressed genes exhibited consistency between groups, thereby validating the reliability of the data (Fig. 2C). Structural perturbations in bacterial periplasmic proteins and alterations in outer membrane lipopolysaccharide (LPS) composition activated the σ²⁴/σ³²-mediated stress response, inducing expression of genes encoding heat-shock proteins (e.g., molecular chaperones for protein folding, proteases for misfolded protein degradation) (31–33). Overexpression of σ²⁴ also triggered lethal cell lysis during log-phase growth (34). Notably, phage infection induced LPS injury and cytoplasmic protein misfolding, thereby activating these sigma factors (35). Transcriptional profiling revealed JSS1_004-mediated suppression of σ²⁴/σ³² and their downstream effectors, including heat-shock chaperones (*dnaK, dnaJ, ibpA, ibpB, groES, hslU, and hslV*) and membrane integrity maintenance genes (*waaY, waaZ, waaP, waaI, waaC, waaB,* and *lapB* for LPS biosynthesis; *murC, murD, murF, and murG* for peptidoglycan synthesis) (Data S1). This coordinated repression likely disabled bacterial protein quality control to prioritize phage protein synthesis and compromised membrane repair systems to facilitate progeny virion release (36–40). JSS1_004 additionally suppressed the transcription of *spoT* and *relA* (Data S1), which encoded (p)ppGpp synthetases critical for stringent response activation, thereby blocking (p)ppGpp biosynthesis (41). This disruption prevented the initiation of the bacterial stringent response even under conditions of rapid consumption of intracellular substances triggered by phage infection (35, 42). Consequently, the characteristic transcriptional shutdown of growth-related genes and ribosomal RNA operons failed to occur, enabling uninterrupted phage replication by maintaining host translational capacity despite nutrient stress (43). Surprisingly, we identified numerous ribosomal subunit genes among the transcriptionally upregulated genes, including those encoding SSU-S1, S2, S6, and S15 and LSU-L3, L5, L9, L7/L12, L21, L25, L30, and L34 subunits. This suggested that JSS1_004 regulated the protein translation pathway to better facilitate the protein synthesis requirements of the JSS1 phage (Data S1). Functional clustering revealed that differentially expressed genes spanned multiple biological processes, including cellular processes, human diseases, genetic information processing, environmental information processes, organismal systems, and metabolism regulation, with no exclusive pathway preference (Fig. S3). This broad-spectrum transcriptional reprogramming primarily served to destabilize host defense systems and suppress stress response pathways. Collectively, these results demonstrate that JSS1_004 orchestrated a dual strategy: (i) inhibition of DndBCDE-FGH system regeneration through *dnd* operon suppression and (ii) global remodeling of host physiology to optimize intracellular conditions for viral propagation.

### ChIP-seq analysis reveals JSS1_004 binding preference for protein synthesis machinery genes

**Fig 3:**
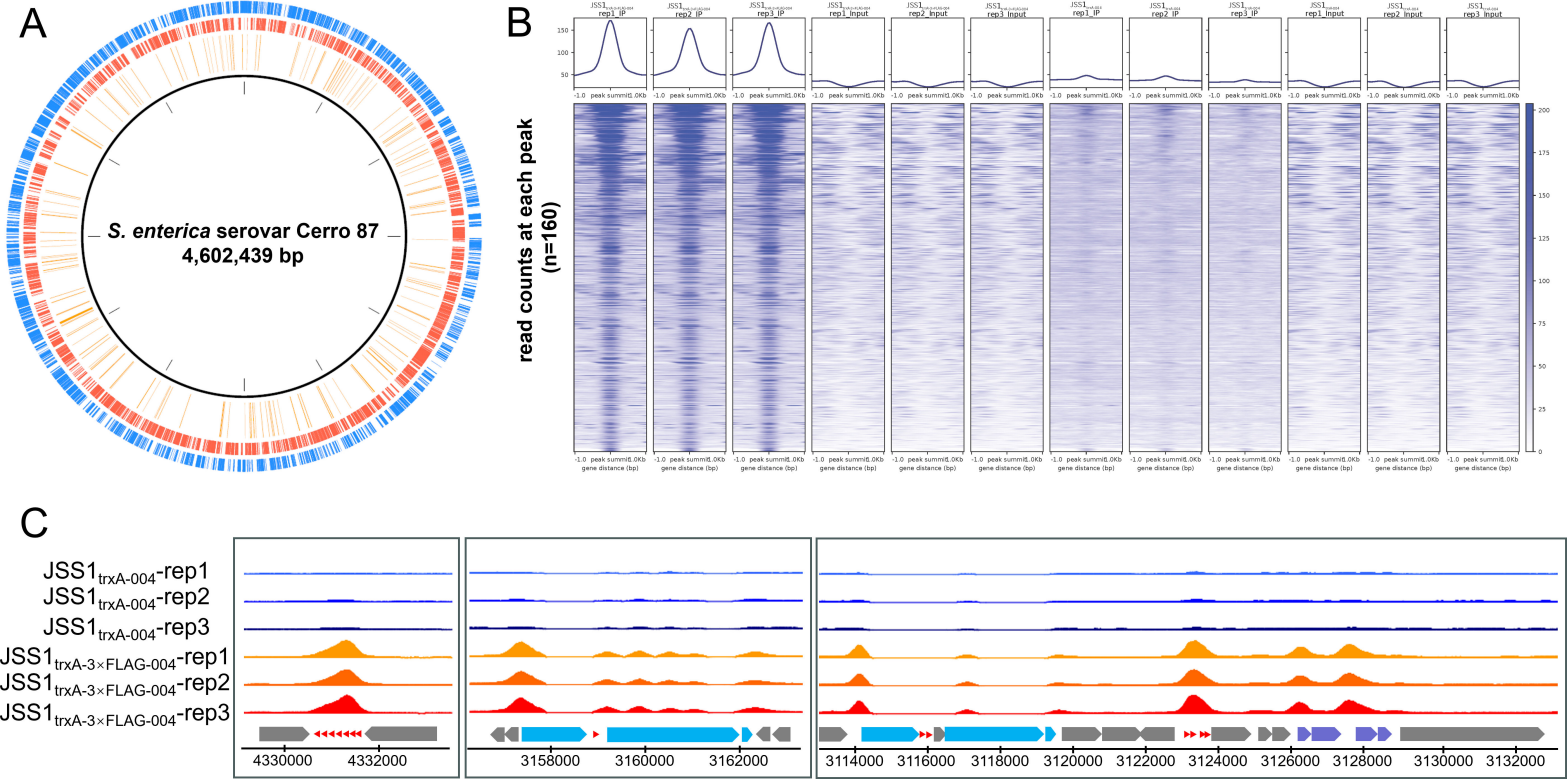
Genomic binding profile of JSS1_004 in Cerro 87. (**A**) Circos plot representation of JSS1_004 binding sites (inner to outer): ChIP-seq-identified binding loci (circle 1, orange), reverse-strand genes (circle 2, red), and forward-strand genes (circle 3, blue). (**B**) Metagene profile of sequencing read density centered on JSS1_004 peak summits. (**C**) Genome browser tracks displaying JSS1_004 ChIP-seq signal intensity at representative genomic loci. tRNA, rRNA, and large ribosomal subunit genes are highlighted in red, blue, and purple, respectively.

Although the N-terminal kinase domain of JSS1_004 was readily purified in our previous study, repeated attempts to isolate full-length JSS1_004 or its C-terminal shutoff domain were unsuccessful. To further characterize the nucleic acid-binding properties of JSS1_004, we generated two JSS1 variants—JSS1_trxA-3×FLAG-004_ (N-terminally 3× FLAG-tagged JSS1_004) and untagged JSS1_trxA-004_—using *trxA*-based homologous recombination strategy (21). Prior to ChIP-seq, Cerro 87 was incubated with either JSS1_trxA-3×FLAG-004_ or JSS1_trxA-004_ at an MOI of 5 for 5 min at 28°C. As anticipated, genome-wide ChIP-seq profiling revealed extensive, non-specific binding of JSS1_004, with 160 binding sites detected across the Cerro 87 genome (Fig. 3A). Analysis of the sequencing output reads showed pronounced binding peaks at these 160 core regions (Fig. 3B). Strikingly, JSS1_004 exhibited pronounced binding preference for genes encoding core protein synthesis machinery components, including tRNAs, rRNAs, and both large (LSU) and small (SSU) ribosomal subunits (Data S2). Genomic analysis identified 84 tRNA, 22 rRNA, 32 LSU, and 25 SSU genes in Cerro 87. Notably, ChIP-seq demonstrated JSS1_004 binding to the majority of these loci, occupying 71/84 tRNA, 15/22 rRNA, 13/32 LSU, and 4/25 SSU genes. This observation exhibited a strong correlation with the upregulated expression of large/small ribosomal subunit (LSU/SSU) genes identified in transcriptomic profiling, further supporting the hypothesis that JSS1_004 reprogrammed host translational machinery to prioritize phage protein synthesis. In genomic regions enriched with tRNA genes, strong binding signals were consistently observed across clustered tRNA loci. For example, a broad peak spanning seven tRNA genes was detected within the region 4,330,700–4,331,500 (Fig. 3C). Similarly, in rRNA gene clusters distributed across the Cerro 87 genome, continuous binding peaks were detected in both promoter regions and gene bodies. A representative example is the locus spanning 3,156,600–3,162,800, which contains three rRNA genes and one tRNA gene (Fig. 3C). Notably, in the genomic region spanning 3,1134,000–3,130,000, which harbors genes encoding tRNAs, rRNAs, and LSU ribosomal proteins, strong binding signals were detected in the promoters of rRNA and LSU genes, as well as across four adjacent tRNA genes (Fig. 3C). Interestingly, this region also harbors genes encoding a translation elongation factor (GW13_PRO3116), a transcription antitermination protein (GW13_PRO3118), and a DNA-direct RNA polymerase β subunit (GW13_PRO3123), suggesting that JSS1_004 may play a role in modulating bacterial RNA and protein processing. Besides, other genes involved in multiple cellular metabolic pathways were also found to be the target of JSS1_004 (Data S2).

ChIP-seq analysis failed to detect direct binding of JSS1_004 to promoter regions or coding sequences of the *dnd* gene cluster, indicating its transcriptional suppression operates through indirect mechanisms. The binding to the promoter region of DNA-directed RNA polymerase β subunit and phosphorylation of the β subunit of RNA polymerase and transcription initiation factors IF2/IF3 by JSS1_004 potentially explained the broad transcriptional repression observed in RNA-seq data despite the absence of promoter-specific binding (29). Notably, ChIP-seq revealed JSS1_004 occupancy at the promoter of σ³² (*rpoH*) genes (Data S2), which exhibited significant transcriptional downregulation in the transcriptome (Data S1), suggesting targeted suppression of heat shock response pathways to prevent the activation of chaperone-mediated protein quality control systems (38). This speculation was further corroborated by JSS1_004 binding to the promoter regions of *hslU* (*GW13_PRO3078*), which encoded key components of the heat shock response machinery. Additionally, JSS1_004 is bound to the promoters of *recA* and *lexA*, key regulators of the SOS pathway responsible for DNA damage repair. Transcriptome data also confirmed the downregulation of the *recA* gene, indicating strategic transcriptional suppression of the SOS response. By repressing *recA* and *lexA*, JSS1_004 prevented the activation of error-prone DNA repair systems and cell cycle arrest, thereby maintaining a replication-permissive intracellular environment for phage propagation (44). These strategic inhibitions likely preserved translational resources for phage structural protein synthesis while blocking stress-induced bacterial defense mechanisms that could impede viral replication. Overall, these findings demonstrated the nucleic acid-binding capacity of JSS1_004 and suggested its potential role in hijacking the host’s protein synthesis machinery.

### Engineering attenuated-cytotoxicity JSS1_004 mutants with antagonistic activity against DndBCDE-FGH restriction system

**Fig 4:**
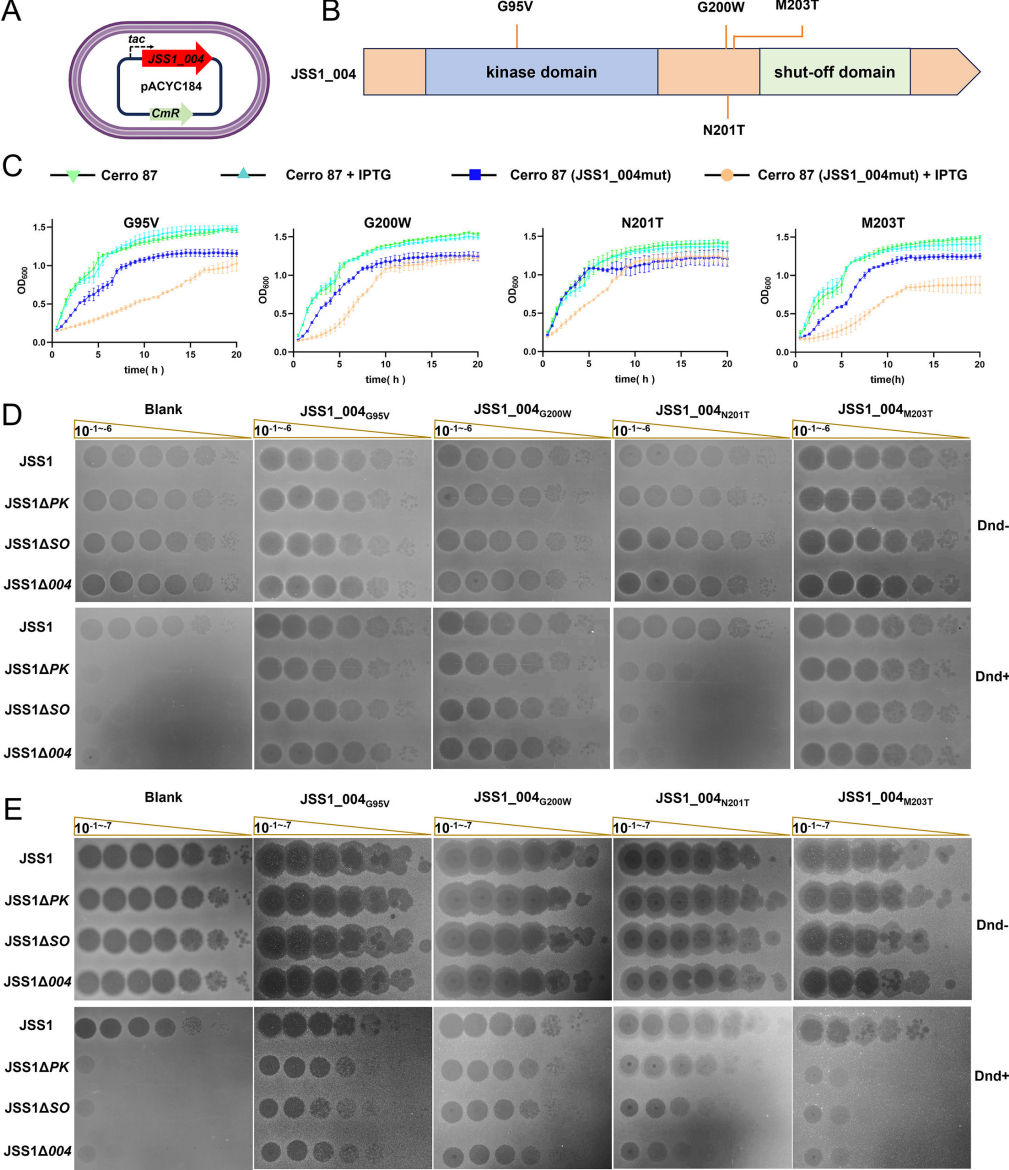
Attenuated JSS1_004 mutants showed reduced cytotoxicity while retaining resistance to the DndBCDE-FGH restriction system. (**A**) Schematic representation of *JSS1_004* gene cloning strategy. (**B**) Domain organization of JSS1_004 protein with sequencing-confined mutation sites (orange dashes) mapped. (**C**) Growth profiles of Cerro 87 and derivatives carrying JSS1_004 mutant under IPTG-inducible or basal expression conditions. (**D** and **E**) Phage plaque formation assays on wild-type Cerro 87 and restriction-deficient mutant XTG103 carrying either empty pACYC184 vector or pACYC184 expressing JSS1_004 mutants. Strains were repressed with 1% (wt/vol) glucose in panel **D** or induced with 0.1 mM IPTG in panel **E**.

Owing to its potent inhibitory efficacy and broad-spectrum antagonistic properties, JSS1_004 represents a promising functional element for engineered phage applications (e.g., phage therapy). To prevent the severe cytotoxicity of JSS1_004, demonstrated by previous studies (29), we choose to clone *JSS1_004* into the pACYC184 plasmid under the *tac* promoter, leveraging low-copy plasmid architecture and *lac* operator repression. Transformant plates were supplemented with 1% (wt/vol) glucose to further suppress basal expression (Fig. 4A). However, we were unable to obtain transformants containing the correct recombinant plasmid. DNA sequencing analysis identified multiple genetic alterations, including insertions or deletions that caused frameshift mutations, as well as base substitutions that generated premature termination codons or missense mutations (e.g., G95V, G200W, N201T, and M203T) (Fig. 4B). These findings highlight the cytotoxic nature of wild-type JSS1_004. Although stability assays confirmed that single point mutations (JSS1_004_G95V_, JSS1_004_G200W_, JSS1_004_N201T_, and JSS1_004_M203T_) alleviated bacterial stress—as evidenced by the absence of new mutations during propagation (Data S1)—IPTG induction at 0.1 mM resulted in observable cell growth inhibition. This effect was particularly pronounced in strains carrying the JSS1_004_M203T_ variant (Fig. 4C).

Furthermore, we assessed the anti-DndFGH activity of these attenuated-cytotoxicity mutants. In stark contrast to the robust protection provided by DndFGH in cells lacking JSS1_004 variants, the restriction activity of DndFGH was significantly compromised in Cerro 87 strains expressing JSS1_004_G95V_, JSS1_004_G200W_, JSS1_004_N201T_, and JSS1_004_M203T_, respectively (Fig. 4D and E). The comparable EOP values observed for JSS1, JSS1Δ*PK*, JSS1Δ*SO,* and JSS1Δ*004* mutants in Cerro 87 strains expressing JSS1_004_G95V_, JSS1_004_G200W_, JSS1_004_N201T_, or JSS1_004_M203T_ suggested that these single amino acid substitutions in JSS1_004 did not affect its DndFGH antagonistic function (Tables 1 and 2). Remarkably, despite the presence of 1% of glucose, Cerro 87 strains carrying JSS1_004_G95V_, JSS1_004_G200W_, or JSS1_004_M203T_ completely abolished DndFGH-dependent phage restriction, demonstrating that even minimal expression of these variants suffices to neutralize DndFGH function (Fig. 4D). Consistent with prior observations, JSS1_004_M203T_ expression under 0.1 mM IPTG induction exerted such potent cytotoxicity in Cerro 87 that it prevented bacterial lawn formation, consequently obscuring plaques from JSS1Δ*PK*, JSS1Δ*SO,* and JSS1Δ*004* mutants (Fig. 4E). These results demonstrated successful engineering of attenuated JSS1_004 mutants via spontaneous attenuation mutations emerging under host tolerance constraints, a process driven by selective pressure imposed during plasmid maintenance in *E. coli*. The JSS1_004 mutants specifically uncoupled cytotoxicity from defense antagonism, providing a template for developing phage-based counter-defense tools with enhanced biocompatibility.

**TABLE 1:** The EOP of phage JSS1 and its derivatives infecting Cerro 87 and the Cerro 87 harboring JSS1_004 mutant strains under repressed environment

Phages	EOP				
	Cerro 87 (pACYC184)	Cerro 87 (G95V)	Cerro 87 (G200W)	Cerro 87 (N201T)	Cerro 87 (M203T)
JSS1	0.64 ± 0.14	0.78 ± 0.05	0.76 ± 0.07	0.76 ± 0.06	0.87 ± 0.05
JSS1Δ*PK*	<10^−5^	0.80 ± 0.11	0.74 ± 0.05	<10^−2^	0.83 ± 0.09
JSS1Δ*SO*	<10^−5^	1.06 ± 0.06	0.78 ± 0.04	<10^−3^	1.01 ± 0.20
JSS1Δ*004*	<10^−5^	0.75 ± 0.12	0.65 ± 0.13	<10^−3^	0.87 ± 0.11

**TABLE 2:** The EOP of phage JSS1 and its derivatives infecting Cerro 87 and the Cerro 87 with JSS1_004 mutant strains under induced condition

Phages	EOP				
	Cerro 87 (pACYC184)	Cerro 87 (G95V)	Cerro 87 (G200W)	Cerro 87 (N201T)	Cerro 87 (M203T)
JSS1	0.85 ± 0.14	1.57 ± 0.48	0.84 ± 0.33	0.76 ± 0.39	0.26 ± 0.07
JSS1Δ*PK*	<10^−5^	0.65 ± 0.23	0.43 ± 0.07	0.69 ± 0.25	<10^−3^
JSS1Δ*SO*	<10^−5^	0.45 ± 0.05	0.37 ± 0.14	0.36 ± 0.08	<10^−3^
JSS1Δ*004*	<10^−5^	1.00 ± 0.43	0.57 ± 0.16	0.79 ± 0.24	<10^−3^

### Attenuated-cytotoxicity JSS1_004 mutants confer M13 phages resistance to DndBCDE-FGH

**Fig 5:**
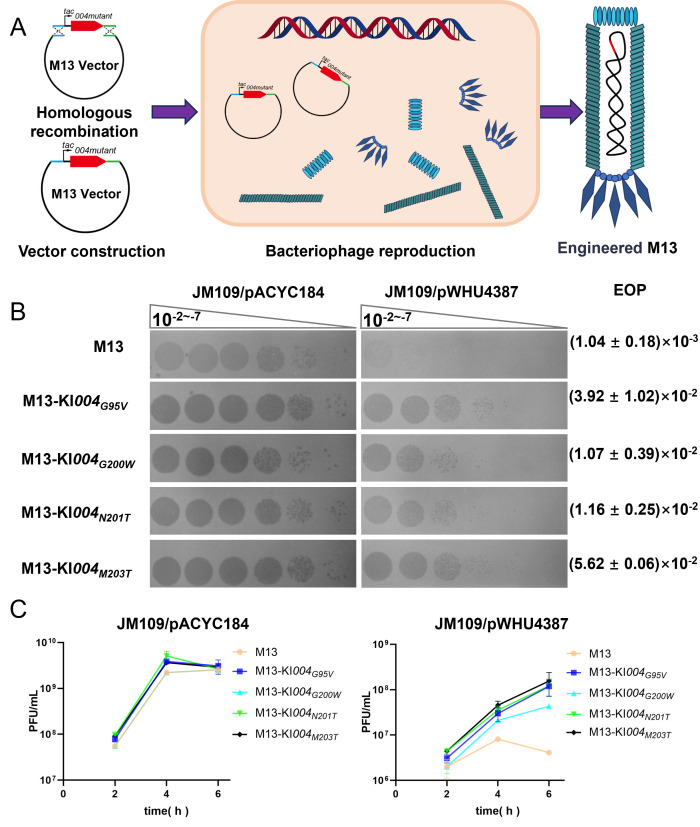
Attenuated JSS1_004 mutants confer DndBCDE-FGH system resistance to engineered M13 phage. (**A**) Schematic illustration of engineered M13 phage construction. (**B**) Plaque formation assay of JSS1_004 mutant-derived M13 derivatives on *E. coli* JM109 strains harboring pACYC184 (empty vector) or pWHU4387 (pACYC184 derivative, carrying *dndBCDE-FGH* from *E. coli* B7A). (**C**) Growth curve analysis of engineered M13 phage in JM109 strains carrying pACYC184 or pWHU4387. Engineered variants exhibit enhanced replication capacity under DndBCDE-FGH restriction pressure, demonstrating acquired resistance to the defense system.

Following the successful construction of JSS1_004 mutants, which retained functionality while exhibiting attenuated cytotoxicity, we integrated them into viral genomes to assess whether this modification could enable heterologous phages to evade the DndBCDE-FGH defense system. We selected M13 filamentous phages as our viral chassis due to their well-established use as versatile building blocks and scaffolds in diverse applications (45). Using *in vitro* homologous recombination, we inserted *tac* promoter-driven JSS1_004 mutant cassettes into the M13 genome between *gene II* and the origin of replication (ori) region. Recombinant constructs were transformed into *E. coli* JM109 for phage propagation, with sequencing confirming stable genomic integration and maintenance of exogenous sequences in progeny virions (Fig. 5A). Functional assessment revealed that engineered phages exhibited enhanced resistance to PT-mediated restriction. In JM109 harboring pWHU4387, which is a pACYC184-derived plasmid carrying the *E. coli* B7A-encoded DndBCDE-FGH (46), wild-type M13 phage exhibited a 1,000-fold decrease in plaque-forming efficiency, producing faint and indistinct plaques. In contrast, engineered variants demonstrated significant resistance: M13-KI*004_G200W_* and M13-KI*004_N201T_* exhibited 10.33-fold ± 3.75-fold and 11.13-fold ± 2.44-fold increased resistance, respectively, whereas M13-KI*004_G95V_* and M13-KI*004_M203T_* severally showed 37.68-fold ± 9.81-fold and 54.09-fold ± 6.01-fold enhancement. Notably, all engineered M13 phage variants formed single, clear plaques even in the presence of DndFGH, indicating functional JSS1_004 mutant expression during host infection (Fig. 5B). Phage replication kinetics further validated this phenotype. In JM109/pACYC184 lacking the DndBCDE-FGH system, wild-type and engineered M13 phages displayed a comparable replication rate, reaching titers of 10^9^ PFU/mL within 4 h. However, under PT-mediated restriction in JM109/pWHU4387, wild-type M13 achieved only 4 × 10^6^ PFU/mL after 6 h. Engineered phages exhibited superior replication: M13-KI*004_G200W_* reached 4 × 10^7^ PFU/mL, whereas the other three mutants surpassed 1.2 × 10^8^ PFU/mL (Fig. 5C). The successful application of attenuated JSS1_004 mutants prompted us to engineer M13 phage by integrating the wild-type *JSS1_004* under the control of the *tac* promoter into its genome, aiming to enhance its capacity to antagonize the DndBCDE-FGH system. However, consistent with prior findings, we were unable to clone the wild-type JSS1_004 into the viral vector. The integrated *JSS1_004* gene acquired multiple mutations, including frameshifts caused by indels (insertions/deletions), premature translational termination, and missense mutations induced by nucleotide substitution (Data Files S2). These data collectively demonstrate that attenuated JSS1_004 mutants serve as effective anti-defense modules, enabling engineered M13 phages to bypass PT-based restriction while maintaining replication fitness. This proof-of-concept study establishes a framework for augmenting therapeutic phages with counter-defense elements to overcome bacterial epigenetic immunity systems.

## DISCUSSION

In this study, we demonstrate that the antagonistic activity of JSS1_004 against DndFGH requires coordinated functions of both its kinase domain and shutoff domain. Although the isolated kinase domain significantly inhibits DndFGH nicking activity through extensive phosphorylation *in vitro*, the JSS1Δ*SO* phage nearly completely loses resistance to DndFGH *in vivo*. This observed discrepancy suggests that phosphorylation alone is insufficient to counteract the DndBCDE-FGH restriction system within the complex cellular environment. Moreover, persistent host-driven transcription/translation of new DndFGH complexes could present a secondary challenge to this antagonistic protein kinase, requiring additional transcriptional repression of the *dndFGH* operon to effectively neutralize restriction activity. Besides, membrane disruption and rapid depletion of intracellular resources by phage infection trigger host stress responses, which activate bacterial self-defense mechanisms. These mechanisms prioritize cellular survival over proliferation, thereby diverting host resources away from processes essential for phage propagation (47, 48). To optimize progeny virion production, phages must strategically rewire host metabolic networks, redirecting energy and precursors toward viral structural protein synthesis and genome replication. We confirmed that JSS1_004-mediated both indirect and direct transcriptional repression of the host genes to support this mechanism. Transcriptomic profiling, together with ChIP-seq, revealed that JSS1_004 broadly regulated host gene expression, creating a cellular state conducive to phage propagation, which includes disrupting membrane repair mechanisms to facilitate progeny release, suppressing stress-responsive pathways (e.g., σ³²-mediated heat-shock response) to attenuate the degradation of misfolded and aberrant proteins to preserve phage structural components, repressing *recA and lexA* expression to block SOS response activation and upregulating transcription of LSU/SSU ribosomal genes to optimize translational capacity for phage structural protein synthesis. ChIP-seq analysis further revealed that JSS1_004 can directly bind to the coding regions or promoter regions of genes related to protein synthesis, indicating its potential to hijack the host’s protein synthesis machinery. This dual-action mechanism explains why individual domains exhibit partial antagonistic activity, whereas their synergistic combination achieves complete suppression by simultaneously inactivating existing DndFGH complexes and blocking *de novo* synthesis.

The potent anti-restriction capacity of JSS1_004 highlights its potential as a universal counter-defense module for phage engineering. Current phage evasion strategies face significant limitations: DNA modification systems (e.g., T4 phage) require complex multi-gene clusters incompatible with modular engineering, whereas DNA mimicry proteins (e.g., T7 phage) only target DNA-binding effectors (49, 50). In contrast, JSS1_004 employs two generalizable mechanisms: non-specific kinase-mediated effector phosphorylation and global transcriptional regulation, providing broad-spectrum anti-defense capability. However, the strong cellular toxicity of wild-type JSS1_004 precludes its cloning in expression vectors, necessitating engineered variants with attenuated cytotoxicity. Notably, phylogenetic analysis reveals an exclusive distribution pattern of JSS1_004 homologs among *Autographiviridae* members, underscoring their co-evolutionary adaptation (29). This taxonomic specificity poses significant challenges for the functional transfer of this anti-defense module to phylogenetically distant phage systems. In this study, we successfully isolated four toxicity-reduced mutants retaining potent anti-DndBCDE-FGH activity. Subsequent successful genomic integration of these variants into M13, a representative filamentous phage, led to markedly increased replication capacity when challenged with PT-based host defense, validating their potential for practical implementation in phage engineering.

Collectively, these findings establish that JSS1_004 employs an essential dual-domain mechanism for DndBCDE-FGH system antagonism, with the shutoff domain playing a predominant role through transcriptional regulation of host genes. Notably, this study advances our understanding of phage counter-defense strategies by (i) elucidating a novel two-pronged anti-restriction mechanism combining post-translational modification and transcriptional regulation, and (ii) establishing a prototype for engineering phage therapeutics with enhanced host adaptability (Fig. S4). These insights expand the repertoire of anti-defense modules available for synthetic phage development and provide new paradigms for overcoming bacterial immunity systems.

## MATERIALS AND METHODS

### Bacterial strains, bacteriophages, and plasmids

All of the strains, bacteriophages, and plasmids used in this study are listed in Table S1. The primers used in this study are listed in Table S2. The *S. enterica* serovar Cerro 87 strain and its derivatives were cultured at 28°C, whereas all *E. coli* strains were grown at 37°C in LB broth or on LB agar (LA) plates. When necessary, antibiotics of appropriate concentrations (25 µg mL^−1^ chloramphenicol and/or 100 µg mL^−1^ ampicillin) were added to the culture medium.

### Plasmid construction

To generate plasmids containing JSS1_004 mutant, the *JSS1_004* gene fragment (1.1 kb) was amplified from JSS1 phage genomic DNA using primers 004 F/R. Simultaneously, the pACYC184-*tac-lac*Z vector backbone (5,541 bp) was amplified with primers 184-Reverse-F/R. The purified gene fragment and linearized vector were recombined using the ClonExpress Ultra One Step Cloning Kit (Vazyme), followed by transformation into *E. coli* DH5α competent cells. Transformants were recovered in LB medium supplemented with 1% (wt/vol) glucose to suppress leaky expression and plated on LB agar containing 25 µg/mL chloramphenicol and 1% glucose. Single colonies were inoculated into glucose-supplemented LB broth for overnight culture. Plasmids were extracted for Sanger sequencing to confirm the presence of desired mutations. Mutant plasmids showing stable genetic profiles were selected for downstream functional assays.

### Construction of mutants of JSS1

JSS1 was serially diluted and spotted onto LB agar plates containing XTG103 strains carrying specific spacers and homologous sequences. After overnight incubation at 28°C, a single plaque was selected and serially diluted with SM buffer. Three single-plaque isolation cycles were performed by mixing the appropriate diluted phage stock with XTG103 in LB broth supplemented with 0.75% agar and overlaid on a preprepared LB agar (1.5%) plate. The mutants were checked by PCR and DNA sequencing.

### Phage plaque assays and efficiency of plating (EOP) calculations

Single colonies of *E. coli* or *S. enterica* strains were inoculated into LB broth overnight at their optimal growth temperatures. Overnight cultures were subcultured (1:100 dilution) in fresh LB medium and grown to mid-log phase (OD_600_ ≈ 0.6). For plaque assays, 300 µL of bacterial suspension was mixed with molten top agar (0.75% agar in LB, maintained at 60°C) and immediately overlaid onto pre-solidified Luria agar (LA) plates. Phage stocks were serially diluted (10-fold gradients) in SM buffer, and aliquots (5 µL) of each dilution were spotted on the agar surface. Plates were incubated overnight under strain-specific temperature conditions. EOP assays were applied to quantify the phage resistance conferred by the defense systems. Briefly, serially diluted phages were mixed with 300 µL of cell culture in LB broth containing 0.75% agar, and the mixture was overlaid on a preprepared LB agar (1.5%) plate. EOP was calculated as the ratio of plaque-forming units (PFUs) on tested cells divided by the number of PFUs on control cells.

### Bacterial growth curve assay

Overnight cultures of *S. enterica* strains were subcultured (1:100 dilution) in LB medium supplemented with 25 µg/mL chloramphenicol and grown to early log phase (OD_600_ ≈ 0.2). For cytotoxicity assessment, 120 µL of log-phase cultures were aliquoted into 96-well plates with experimental groups receiving a final concentration of 0.1 mM IPTG, whereas controls were supplemented with an equivalent volume of ultrapure water. Bacterial growth was monitored by measuring OD_600_ absorbance at 30-min intervals over 20 h using a microplate spectrophotometer (Synergy H1MF, BioTek) maintained at 28°C. For phage infection kinetics, log-phase cultures were co-cultured with phages at multiplicities of infection (MOIs) of 0, 0.1, 0.5, and 1 in 96-well plates to track the growth profiles via OD_600_ measurements for 12 h.

### Construction of engineered M13

Primers were used to amplify the M13 phage backbone and *JSS1_004* gene carrying certain point mutations, followed by homologous recombination using ClonExpress Ultra One Step Cloning Kit (Vazyme, China). The resulting construct was transferred into JM109 for further phage amplification and assembly. Individual phage plaques were isolated and subjected to three rounds of plaque purification. The engineered phage genome was subsequently verified by DNA sequencing.

### Phage growth curve assay

Overnight cultures of JM109/pACYC184 and JM109/pWHU4387 were subcultured (1:100 dilution) in fresh LB medium and grown to mid-log phase (OD_600_ ≈ 0.6). M13 phage was added at an MOI of 0.1, and the mixture was incubated statically at 37°C. At designated time points, aliquots were collected and centrifuged at 10,000 × *g* for 1 min, and the supernatant was filtered through a 0.22 µm membrane (Millipore). To determine phage titer, the filtered supernatant was serially diluted, mixed with 300 µL of JM109 culture in molten LB soft agar (0.75%), and overlaid onto pre-poured LB agar (1.5%) plates. Plaques were calculated as plaque-forming units (PFU/mL) after overnight incubation at 37°C.

### Transcriptome and data analysis

Overnight cultures of *S. enterica* serovar Cerro 87 were subcultured at a ratio of 1:100 in LB medium supplemented with 1 mM MgCl₂ and CaCl₂, then grown to mid-exponential phase (OD_600_ ≈ 0.5). Phages JSS1 or JSS1Δ*004* were introduced at an MOI of 10 and incubated statically at 28°C for 10 min to maximize adsorption efficiency. Bacterial-phage mixtures were pelleted by centrifugation (10,000 × *g*, 2 min), flash-frozen in liquid nitrogen, and stored at −80°C until processing, with triplicate biological replicates per condition. Samples were submitted to Majorbio Bio-Pharm Technology Co., Ltd. (Shanghai, China) for RNA sequencing. Ribosomal RNA (rRNA) was depleted from mixed bacterial RNA samples using the RiboCop rRNA Depletion Kit (Lexogen), optimized for prokaryotic transcriptome profiling. Stranded mRNA libraries were constructed with the Illumina Stranded mRNA Prep Kit following the manufacturer’s protocol and then subjected to the Illumina NovaSeq 6000 platform to generate approximately 9 Gb of raw data per sample. Differential gene expression analysis was performed via the Majorbio Cloud Platform (v2.0) using DESeq2.

### ChIP-seq and data analysis

Chromatin immunoprecipitation followed by high-throughput sequencing (ChIP-seq) using commercial anti-FLAG antibody was performed as previously described (51), with minor modifications. Briefly, overnight cultured Cerro 87 were subcultured at a 1:100 ratio in LB medium, then grown to mid-exponential phase (OD_600_ = 0.5). JSS _trxA-004_ and JSS_trxA-3×FLAG-004_, either expressing wild-type or 3× FLAG-tagged of JSS1_004 at the N-terminus, were added at an MOI of 5, respectively, and the mixture was incubated at 28°C for 5 min. Cross-linking was performed by adding 1% (vol/vol) formaldehyde to the culture and incubating for 20 min at room temperature. The reaction was quenched by adding 500 mM glycine and incubating for an additional 10 min. Then, the cells were harvested by centrifuging at 6,000 × *g* for 5 min at 4°C. The pellet was resolved in 500 µL lysis buffer (50 mM HEPES-KOH [pH 7.5], 150 mM NaCl, 1 mM EDTA, 1% Triton X-100, 0.1% sodium deoxycholate and 0.1% SDS), and the genome was sheared by sonication at 95% amplitude for 10 min, setting with 10 s pulses and 20 s cooling intervals (Qsonica Q800R sonicator) to generate DNA fragments of 200–1,000 bp. Cleared lysates were obtained by centrifugation at 13,000 × *g* for 30 min at 4°C. For each immunoprecipitation reaction, 50 µL lysate was diluted with 450 µL lysis buffer, and 2 µg anti-FLAG antibody (Servicebio) was added and incubated at room temperature for 1.5 h with rotation. Then, 25 µL pre-washed protein A beads (Cytiva, Cat#10296774) were added with an additional 1.5 h of incubation. The protein-antibody-beads complex was transformed to a spin-X centrifuge tube filter and sequentially washed twice with lysis buffer, once with high-salt FA buffer (50 mM HEPES-KOH [pH 7.5], 500 mM NaCl, 1 mM EDTA, 1% Triton X-100, 0.1% sodium deoxycholate, and 0.1% SDS), once with LiCl ChIP wash buffer (10 mM Tris-HCl [pH 8.0], 250 mM LiCl, 1 mM EDTA, 0.5% Igepal CA-630, and 0.5% sodium deoxycholate) and once with TE buffer (10 mM Tris-HCl [pH 8.0], and 1 mM EDTA). Then, the sample was eluted with 100 µL ChIP elution buffer (50 mM Tris-HCl [pH 7.5], 10 mM EDTA, and 1% SDS) at 65°C for 1 h. Eluates were incubated at 65°C overnight to reverse crosslinks. DNA was recovered using the QIAgen PCR clean-up kit, and the immunoprecipitated DNA was sent to Novogene (Novogene Co., Ltd., China) for DNA library preparation and subsequent sequencing.

FastQC was used to assess the quality of raw sequencing reads. Adapter trimming and quality filtering were performed using Fastp to generate high-quality clean reads, which were subsequently re-evaluated with FastQC. Clean reads were aligned to the Cerro 87 reference genome (GenBank accession: CP008925.1) using Burrows-Wheeler Aligner (BWA). BAM files were converted to BigWig (.bw) format for visualization in the Integrated Genome Browser (IGB). Peak calling was conducted using MACS2 (*q*-value ≤ 0.05). Differential binding analysis was performed with DiffBind, applying thresholds of FDR < 0.05 and |log_2_ fold change| > 0.585 to identify statistically significant ChIP-enriched regions compared with the negative control. For peak annotation, promoter regions were defined as ±300 bp surrounding the start codon of each gene.

## Data Availability

The RNA-seq data and ChIP-seq data have been deposited in the Sequence Read Archive under accession number PRJNA1254868 and PRJNA1254703, respectively. Data underlying the findings of this study will be available from the corresponding authors.

## SUPPLEMENTAL MATERIAL

Fig. S1 mbio.01386-25-s0001.docx Verification of JSS1ΔPK and JSS1ΔSO mutants.

Fig. S2 mbio.01386-25-s0002.docx Cerro 87 strains that survived the JSS1 phage infection developed resistance to subsequent infections by the JSS1 phage.

Fig. S3 mbio.01386-25-s0003.docx KEGG pathway analysis of differentially expressed genes in Cerro 87 following JSS1/JSS1Δ004 phage infection.

Fig. S4 mbio.01386-25-s0004.docx Mechanism model of JSS1_004 antagonizing DndFGH and its application.

Table S1 mbio.01386-25-s0005.docx Strains, phages, and plasmids used in this study.

Table S2 mbio.01386-25-s0006.docx Primers used in this study.

File S1 mbio.01386-25-s0007.pdf Sequencing files for plasmid stability test of JSS1_004 mutant strain.

File S2 mbio.01386-25-s0008.pdf Sequencing results of recombinant phages after wild-type JSS1_004 was integrated into the M13 genome.

Data S1 mbio.01386-25-s0009.xlsx RNA-seq data.

Data S2 mbio.01386-25-s0010.xlsx ChIP-seq data.

ASM does not own the copyrights to Supplemental Material that may be linked to, or accessed through, an article. The authors have granted ASM a non-exclusive, world-wide license to publish the Supplemental Material files. Please contact the corresponding author directly for reuse.
