# Supplementary material for: Mechanistic insights into JSS1_004-mediated antagonism of the DndBCDE-FGH restriction system and engineering applications
Source: mBio. 2025 Jul 14;16(8):e01386-25. doi: 10.1128/mbio.01386-25 (PMC12345140; doi:10.1128/mbio.01386-25)
Supplement: Fig. S1 — Verification of JSS1ΔPK and JSS1ΔSO mutants. [file mbio.01386-25-s0001.docx]

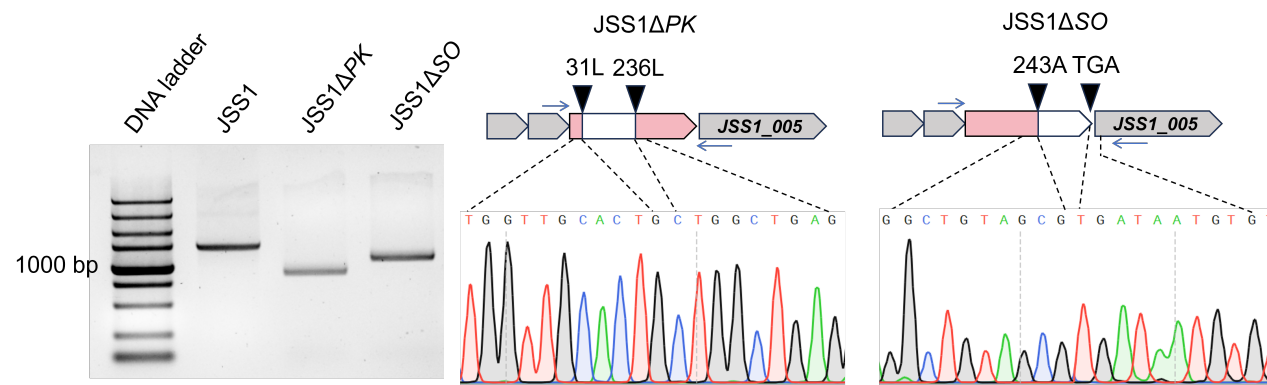


**Fig. S1 Verification of JSS1Δ*PK* and JSS1Δ*SO* mutants.** Validation of kinase and shut-off domain knockouts in the genome of phage JSS1 via PCR amplification and Sanger sequencing using the 004KO-R/F primer pairs.
