# Supplementary material for: Mechanistic insights into JSS1_004-mediated antagonism of the DndBCDE-FGH restriction system and engineering applications
Source: mBio. 2025 Jul 14;16(8):e01386-25. doi: 10.1128/mbio.01386-25 (PMC12345140; doi:10.1128/mbio.01386-25)
Supplement: Fig. S2 — Cerro 87 strains that survived the JSS1 phage infection developed resistance to subsequent infections by the JSS1 phage. [file mbio.01386-25-s0002.docx]

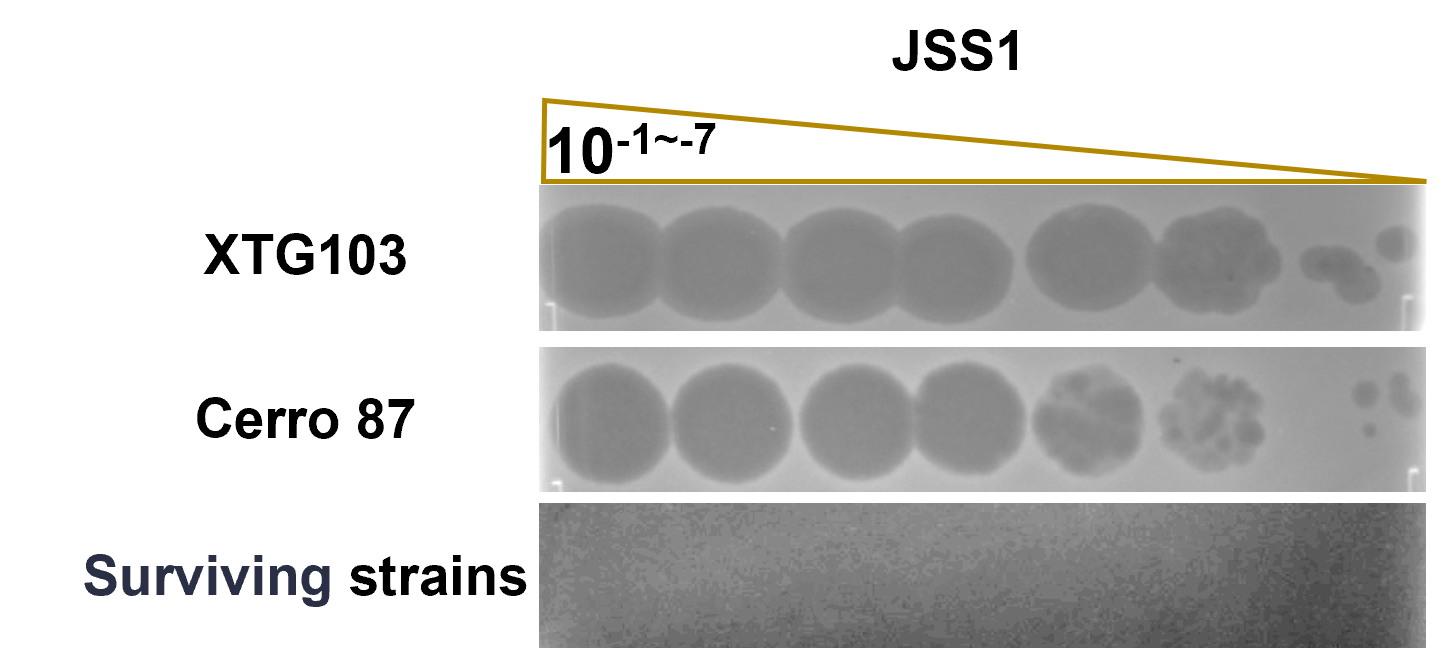


**Fig. S2 Cerro 87 strains that survived the JSS1 phage infection developed resistance to subsequent infections by the JSS1 phage.**
