# Supplementary material for: Mechanistic insights into JSS1_004-mediated antagonism of the DndBCDE-FGH restriction system and engineering applications
Source: mBio. 2025 Jul 14;16(8):e01386-25. doi: 10.1128/mbio.01386-25 (PMC12345140; doi:10.1128/mbio.01386-25)
Supplement: Fig. S3 — KEGG pathway analysis of differentially expressed genes in Cerro 87 following JSS1/JSS1Δ004 phage infection. [file mbio.01386-25-s0003.docx]

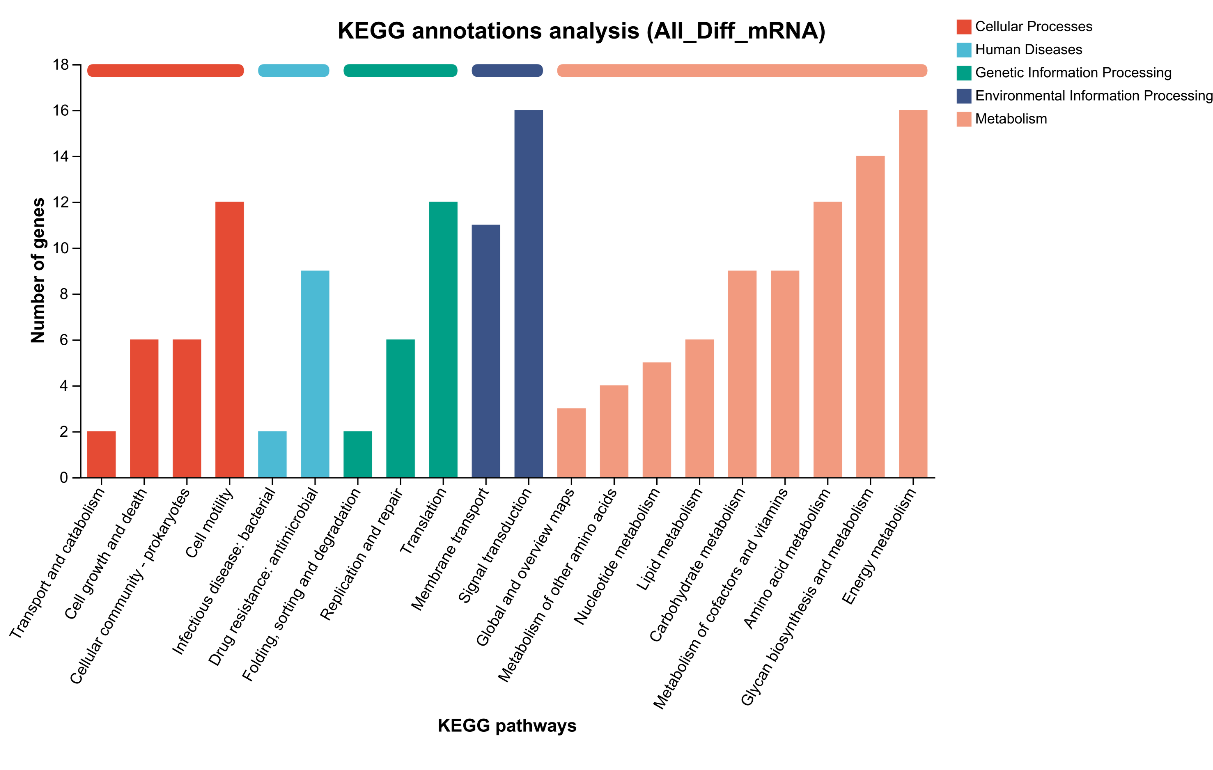


**Fig. S3 KEGG pathway analysis of differentially expressed genes in Cerro 87 following JSS1/JSS1Δ*004* phage infection.** The differentially expressed genes were distributed across five functions, with no significant bias observed.
