# Supplementary material for: Mechanistic insights into JSS1_004-mediated antagonism of the DndBCDE-FGH restriction system and engineering applications
Source: mBio. 2025 Jul 14;16(8):e01386-25. doi: 10.1128/mbio.01386-25 (PMC12345140; doi:10.1128/mbio.01386-25)
Supplement: Fig. S4 — Mechanism model of JSS1_004 antagonizing DndFGH and its application. [file mbio.01386-25-s0004.docx]

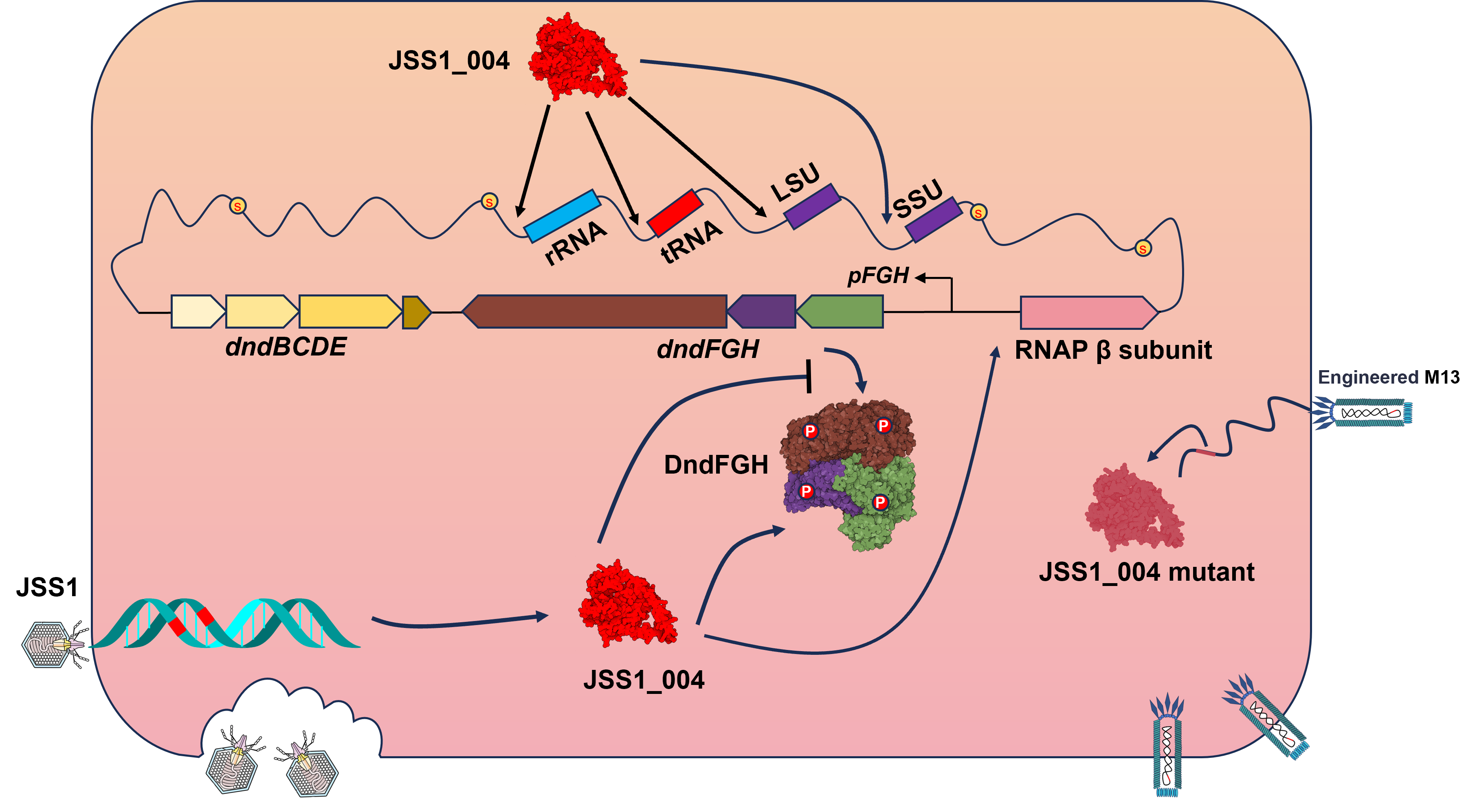


**Fig. S4 Mechanism model of JSS1_004 antagonizing DndFGH and its application.** JSS1_004 antagonizes the DndBCDE-FGH system through kinase-mediated phosphorylation and transcriptional regulation of host genes via its shut-off domain. Phosphorylation directly inhibits the activity of DndFGH, while the shut-off domain suppresses the sustained production of DndFGH. The attenuated JSS1_004 mutants can be employed to engineer the M13 phage, conferring resistance to the DndBCDE-FGH system.
