# Supplementary material for: Mechanistic insights into JSS1_004-mediated antagonism of the DndBCDE-FGH restriction system and engineering applications
Source: mBio. 2025 Jul 14;16(8):e01386-25. doi: 10.1128/mbio.01386-25 (PMC12345140; doi:10.1128/mbio.01386-25)
Supplement: Table S1 — Strains, phages, and plasmids used in this study. [file mbio.01386-25-s0005.docx]

**TABLE S1. Strains, phages and plasmids used in this study.**

| **Name** | **Characteristics** | **Source or reference** |
| --- | --- | --- |
| **Strains** |  |  |
| ***E. coli*** |  |  |
| JM109 | *endA1 glnV44 thi-1 relA1 gyrA96 recA1 mcrB^+^ Δ (lac-proAB) e14- [F' traD36 proAB^+^ lacI^q^ lacZΔM15] hsdR17 (r_K_^-^m_K_^+^)* | Sangon Biotech |
| DH5α | *F^-^ endA1 glnV44 thi-1 recA1 relA1 gyrA96 deoR nupG purB20 φ80dlacZΔM15 Δ (lacZYA-argF) U169, hsdR17 (rK–mK+), λ^-^* | Sangon Biotech |
| ***S. enterica*** |  |  |
| *S. enterica* serovar Cerro 87 | Wild type, *dndB-H*, d(G_PS_A)/d(G_PS_T) | (1) |
| XTG103 | Cerro 87 derivative, *dndB-H* deletion mutant | (1) |
| JSS101 | Cerro 87 derivative, trxA deletion mutant | (2) |
| **Phages** |  |  |
| JSS1 | Cerro 87 phage, *Podoviridae*, lytic | (2) |
| JSS1Δ*004* | JSS1 derivative, *JSS1_004* deficient strain | (2) |
| JSS1Δ*PK* | JSS1 derivative, the kinase domain of *JSS1_004* deficient strain | This study |
| JSS1ΔSO | JSS1 derivative, the shut-off domain of *JSS1_004* deficient strain | This study |
| JSS1_trxA-3×FLAG-004_ | JSS1 derivative, carrying *trxA* and N-terminal 3 × Flag tagged *JSS1_004* | This study |
| JSS1_trxA-004_ | JSS1 derivative, carrying *trxA* | This study |
| PT1 | *S. enterica* phages, Myoviridae, lytic | (3) |
| M13 | *E. coli* phage, filamentous phage | ATCC |
| M13-KI*004_G95V_* | M13 derivative, knocked in the *JSS1_004_G95V_* gene with *tac* promoter | This study |
| M13-KI*004_G200W_* | M13 derivative, knocked in the *JSS1_004 _G200W_* gene with *tac* promoter | This study |
| M13-KI*004_N201T_* | M13 derivative, knocked in the *JSS1_004 _N201T_* gene with *tac* promoter | This study |
| M13-KI*004_M203T_* | M13 derivative, knocked in the *JSS1_004 _M203T_* gene with *tac* promoter | This study |
| **Plasmids** |  |  |
| pBluescript II SK (+) | Cloning vector, Amp^r^. | (4) |
| pWHU5000 | pBluescript II SK (+) derivative harboring the homologous arm for deletion of the kinase domain in JSS1_004. | This study |
| pWHU5001 | pBluescript II SK (+) derivative harboring the homologous arm for deletion of the shut-off domain in JSS1_004. | This study |
| pACYC184 | Cloning vector, Cm^r^. | (5) |
| pWHU4387 | pACYC184 derivative carrying *dndBCDE-FGH* with the native promoter from *E. coli* B7A | (6) |
| pWHU5002 | pACYC184 derivative carrying *JSS1_004 _G95V_* gene with *tac* promoter | This study |
| pWHU5003 | pACYC184 derivative carrying*JSS1_004 _G200W_* gene with *tac* promoter | This study |
| pWHU5004 | pACYC184 derivative carrying *JSS1_004 _N201T_* gene with *tac* promoter | This study |
| pWHU5005 | pACYC184 derivative carrying *JSS1_004 _M203T_* gene with *tac* promoter | This study |
| pWHU5006 | pACYC184 derivative carrying *SpCas9* from *S. pyogenes* SF370 and spacer targeting the kinase domain of *JSS1_004* gene | This study |
| pWHU5007 | pACYC184 derivative carrying *SpCas9* from *S. pyogenes* SF370 and spacer targeting the shut-off domain of *JSS1_004* gene | This study |
| pWHU5008 | pBluescript II SK (+) derivative harboring the homologous arm for knocked in *trxA* and N-terminal 3 × Flag tagged *JSS1_004* | This study |
| pWHU5009 | pBluescript II SK (+) derivative harboring the homologous arm for knocked in *trxA* | This study |
| pWHU5010 | pACYC184 derivative carrying *lacZ* fused with *P_tac_* promoter | This study |
| pWHU5011 | pACYC184 derivative carrying *lacZ* fused with the native promoter of *dndFGH* from Cerro 87 | This study |

**References**

1. Xu T, Yao F, Zhou X, Deng Z, You D. 2010. A novel host-specific restriction system associated with DNA backbone S-modification in Salmonella. Nucleic Acids Res 38:7133-41.

2. Jiang S, Chen C, Huang W, He Y, Du X, Wang Y, Ou H, Deng Z, Xu C, Jiang L, Wang L, Chen S. 2024. A widespread phage-encoded kinase enables evasion of multiple host antiphage defence systems. Nat Microbiol 9:3226-3239.

3. Tang Y, Wu D, Zhang Y, Liu X, Chu H, Tan Q, Jiang L, Chen S, Wu G, Wang L. 2024. Molecular basis of the phosphorothioation-sensing antiphage defense system IscS-DndBCDE-DndI. Nucleic Acids Res 52:13594-13604.

4. Alting-Mees MA, Short JM. 1989. pBluescript II: gene mapping vectors. Nucleic Acids Res 17:9494.

5. Rose RE. 1988. The nucleotide sequence of pACYC184. Nucleic Acids Res 16:355.

6. Jiang S, Chen K, Wang Y, Zhang Y, Tang Y, Huang W, Xiong X, Chen S, Chen C, Wang L. 2023. A DNA phosphorothioation-based Dnd defense system provides resistance against various phages and is compatible with the Ssp defense system. 14:e00933-23.
