# Supplementary material for: Mechanistic insights into JSS1_004-mediated antagonism of the DndBCDE-FGH restriction system and engineering applications
Source: mBio. 2025 Jul 14;16(8):e01386-25. doi: 10.1128/mbio.01386-25 (PMC12345140; doi:10.1128/mbio.01386-25)
Supplement: Table S2 — Primers used in this study. [file mbio.01386-25-s0006.docx]

**TABLE S2. Primers used in this study.**

| **Primers** | **Sequence (5’-3’)** |
| --- | --- |
| **Construction of pWHU5000** | |
| PK-1 | GTCGACGGTATCGATAAGCTTAGGATCTATGAACTACACCGA |
| PK-2 | CGCTACAGCCTCGACCTCAGCCAGCAGTGCAACCAGAAGAGGCTGGC |
| PK-3 | GCCAGCCTCTTCTGGTTGCACTGCTGGCTGAGGTCGAGGCTGTAGCG |
| PK-4 | CGCTCTAGAACTAGTGGATCCTGCGGTTAACCTGAAAGGTTCC |
| **Construction of pWHU5001** | |
| SO-1 | GTCGACGGTATCGATAAGCTTGTACATCACTGACCCGGTTAGTT |
| SO-2 | GACCTTGAAGATACACATTATCACGCTACAGCCTCGACCTCAGCCA |
| SO-3 | TGGCTGAGGTCGAGGCTGTAGCGTGATAATGTGTATCTTCAAGGTC |
| SO-4 | CGCTCTAGAACTAGTGGATCCTGGTGCGTTGATTACGTTAGT |
| **Construction of pWHU5002 to pWHU5005** | |
| 004-F | CTAAAGAGGAGAAAGGATCTATGAACTACACCGACATTCA |
| 004-R | GCGTCCGGCGTAGAGGATCCTCAGCCCATCAGGTGAGCGT |
| 184-Reverse-F | GGATCCTCTACGCCGGACGCATC |
| 184-Reverse-R | AGATCCTTTCTCCTCTTTAGATC |
| **Construction of pWHU5006** | |
| PK-SP-1 | AAACGTGAACGGTGAGACAAGCGAG |
| PK-SP-2 | AAAACTCGCTTGTCTCACCGTTCAC |
| **Construction of pWHU5007** | |
| SO-SP-1 | AAACCATGACTTCTGGTTACGCAAG |
| SO-SP-2 | AAAACTTGCGTAACCAGAAGTCATG |
| **Construction of pWHU5008** | |
| 5008-1 | GTCGACGGTATCGATAAGCTTCGCTGAAGAAAGGCTACAGGTCT |
| 5008-2 | TTATTTCACCGGATACGCTTTCGTTAAGAAAGAGCGTTCAATGTAT |
| 5008-3 | ATACATTGAACGCTCTTTCTTAACGAAAGCGTATCCGGTGAAATAA |
| 5008-4 | AAGAGCGTTCAATGTATTGGTTATTACGCCAGATTGGCGTCGAGAA |
| 5008-5 | TTCTCGACGCCAATCTGGCGTAATAACCAATACATTGAACGCTCTT |
| 5008-6 | TCACCGTCATGGTCTTTGTAGTCCATTAGATTGTGTCCTATTTAAG |
| 5008-7 | CTTAAATAGGACACAATCTAATGGACTACAAAGACCATGACGGTGA |
| 5008-8 | GCCTGAATGTCGGTGTAGTTCATGGAACCACCGCCGCCGCTACCAC |
| 5008-9 | GTGGTAGCGGCGGCGGTGGTTCCATGAACTACACCGACATTCAGGC |
| 5008-10 | CGCTCTAGAACTAGTGGATCCTCACGCATCAGTGCACCCAATGT |
| **Construction of pWHU5009** | |
| 5009-1 | GTCGACGGTATCGATAAGCTTCGCTGAAGAAAGGCTACAGGTCT |
| 5009-2 | TTATTTCACCGGATACGCTTTCGTTAAGAAAGAGCGTTCAATGTAT |
| 5009-3 | ATACATTGAACGCTCTTTCTTAACGAAAGCGTATCCGGTGAAATAA |
| 5009-4 | AAGAGCGTTCAATGTATTGGTTATTACGCCAGATTGGCGTCGAGAA |
| 5009-5 | TTCTCGACGCCAATCTGGCGTAATAACCAATACATTGAACGCTCTT |
| 5009-6 | CGCTCTAGAACTAGTGGATCCTCACGCATCAGTGCACCCAATGT |
| **Construction of pWHU5010** | |
| 5010-1 | TTTGACAGCTTATCATCGATAAGCTTGCTAACTTACATTAATTGCGTT |
| 5010-2 | AGAAGCATTGGTGCACCGTGCAGCAAAACCTTTCGCGGTATGGC |
| 5010-3 | GCCATACCGCGAAAGGTTTTGCTGCACGGTGCACCAATGCTTCT |
| 5010-4 | GTGAATCCGTAATCATGGTCATAGATCCTTTCTCCTCTTTAGAT |
| 5010-5 | ATCTAAAGAGGAGAAAGGATCTATGACCATGATTACGGATTCAC |
| 5010-6 | CACGATGCGTCCGGCGTAGAGGATCCTTATTTTTGACACCAGACCAAC |
| **Construction of pWHU5011** | |
| 5011-1 | TTTGACAGCTTATCATCGATAAGCTTGCTAACTTACATTAATTGCGTT |
| 5011-2 | CATTTACGTAACTTTCATGAAATGCAAAACCTTTCGCGGTATGGC |
| 5011-3 | GCCATACCGCGAAAGGTTTTGCATTTCATGAAAGTTACGTAAATG |
| 5011-4 | GGAATTGTTATCCGCTCACAATTCCAACTTCATGTTCTCTAAAGTAAG |
| 5011-5 | CTTACTTTAGAGAACATGAAGTTGGAATTGTGAGCGGATAACAATTCC |
| 5011-6 | GTGAATCCGTAATCATGGTCATGGTATATCTCCTTCTTAAAG |
| 5011-7 | CTTTAAGAAGGAGATATACCATGACCATGATTACGGATTCAC |
| 5011-8 | CACGATGCGTCCGGCGTAGAGGATCCTTATTTTTGACACCAGACCAAC |
| **Construction of Engineered M13 phage** | |
| 004mut-F | CAATCAGCTGTTGCCCGTCTCTGGATAATGTTTTTTGCGCCGACAT |
| 004mut-R | GTTAAATTTTTGTTCAGCCCATCAGGTGAGCGTCAAGTACCTTGTCGATG |
| M13-Reverse-F | TTGACGCTCACCTGATGGGCTGAACAAAAATTTAACGCGAATTTTAACA |
| M13-Reverse-R | CATTATCCAGAGACGGGCAACAGCTGATTGCCCTTCACCGCCTG |
| **Verification of Engineered M13 phage** | |
| M13KI-F | AACTGGAACAACACTCAACCCTATC |
| M13KI-R | AGCATGTCAATCATATGTACCCCGG |
| **Verification of JSS1 mutants** | |
| 004KO-R | TGAAGAAAGGCTACAGGTCTA |
| 004KO-F | CTTCGTGTTCTAACTCAAGCT |
