# Supplementary material for: Mechanistic insights into JSS1_004-mediated antagonism of the DndBCDE-FGH restriction system and engineering applications
Source: mBio. 2025 Jul 14;16(8):e01386-25. doi: 10.1128/mbio.01386-25 (PMC12345140; doi:10.1128/mbio.01386-25)
Supplement: File S1 — Sequencing files for plasmid stability test of JSS1_004 mutant strain. [file mbio.01386-25-s0007.pdf]

5′ gaattccggatgagcattcatcaggcgggcaagaatgtgaataaaggccggataaaacttgtgcttatttttctttacggtcttt 85
3′ cttaaggcctactcgtaagtagtccgcccgttcttacacttatttccggcctattttgaacacgaataaaaagaaatgccagaaa

70 65 60 55 50
F E P H A N M L R A L I H I F A P Y F K H K N K K V T K
CmR

aaaaaggccgtaatatccagctgaacggtctggttataggtacattgagcaactgactgaaatgcctcaaaatgttctttacgat 170
tttttccggcattataggtcgacttgccagaccaatatccatgtaactcgttgactgactttacggagttttacaagaaatgcta

45 40 35 30 25 20
L F A T I D L Q V T Q N Y T C Q A V S Q F A E F H E K R H
CmR

gccattgggatatatcaacggtggtatatccagtgatttttttctccatttagcttccttagctcctgaaaatctcgataactc 255
cggtaaccctatatagttgccaccatataggtcactaaaaaaagaggtaaatcgaaggaatcgaggacttttagagctattgag

15 10 5 1
W Q S I D V T T Y G T I K K E M
CmR
cat promoter

aaaaaatacgcccggtagtgatcttatttcattatggtgaaagttggaacctcttacgtgccgatcaacgtctcattttcgccaa 340
tttttatgcgggccatcactagaataaagtaataccactttcaaccttggagaatgcacggctagttgcagagtaaaagcggtt

cat promoter

aagttggcccagggcttcccggtatcaacagggacaccaggatttatttattctgcgaagtgatcttccgtcacaggtatttatt 425
ttcaaccgggtcccgaagggccatagttgtccctgtggtcctaaataaataagacgcttcactagaaggcagtgtccataaataa

cggcgcaaagtgcgtcgggtgatgctgccaacttactgatttagtgtatgatggtgtttttgaggtgctccagtggcttctgttt 510
gccgcgtttcacgcagcccactacgacggttgaatgactaaatcacatactaccacaaaaactccacgaggtcaccgaagacaaa

ctatcagctgtccctcctgttcagctactgacggggtggtgcgtaacggcaaaagcaccgccggacatcagcgctagcggagtgt 595
gatagtcgacagggaggacaagtcgatgactgccccaccacgcattgccgttttcgtggcggcctgtagtcgcgatcgcctcaca

atactggcttactatgttggcactgatgagggtgtcagtgaagtgcttcatgtggcaggagaaaaaaggctgcaccggtgcgtca 680
tatgaccgaatgatacaaccgtgactactcccacagtcacttcacgaagtacaccgtcctcttttttccgacgtggccacgcagt

gcagaatatgtgatacaggatatattccgcttcctcgctcactgactcgctacgctcggtcgttcgactgcggcgagcggaaatg 765
cgtcttatacactatgtcctatataaggcgaaggagcgagtgactgagcgatgcgagccagcaagctgacgccgctcgcctttac

gcttacgaacggggcggagatttcctggaagatgccaggaagatacttaacagggaagtgagagggccgcggcaaagccgttttt 850
cgaatgcttgccccgcctctaaaggaccttctacggtccttctatgaattgtcccttcactctcccggcgccgtttcggcaaaaa

p15A ori

ccataggctccgcccccctgacaagcatcacgaaatctgacgctcaaatcagtggtggcgaaacccgacaggactataaagatac 935
ggtatccgaggcggggggactgttcgtagtgctttagactgcgagtttagtcaccaccgctttgggctgtcctgatatttctatg

p15A ori

caggcgtttccccctggcggctccctcgtgcgctctcctgttcctgcctttcggtttaccggtgtcattccgctgttatggccgc 1020
gtccgcaaagggggaccgccgagggagcacgcgagaggacaaggacggaaagccaaatggccacagtaaggcgacaataccggcg

p15A ori

gtttgtctcattccacgcctgacactcagttccgggtaggcagttcgctccaagctggactgtatgcacgaacccccgttcagt 1105
caaacagagtaaggtgcggactgtgagtcaaggcccatccgtcaagcgaggttcgacctgacatacgtgcttgggggcaagtca

p15A ori

```
ccgaccgctgcgccttatccggtaactatcgtcttgagtccaacccggaaagacatgcaaaagcaccactggcagcagccactgg   1190
ggctggcgacgcggaataggccattgatagcagaactcaggttgggcctttctgtacgttttcgtggtgaccgtcgtcggtgacc
< p15A ori

taattgatttagaggagttagtcttgaagtcatgcgccggttaaggctaaactgaaaggacaagttttggtgactgcgctcctcc   1275
attaactaaatctcctcaatcagaacttcagtacgcggccaattccgatttgactttcctgttcaaaaccactgacgcgaggagg
< p15A ori

aagccagttacctcggttcaaagagttggtagctcagagaaccttcgaaaaaccgccctgcaaggcggttttttcgttttcagag   1360
ttcggtcaatggagccaagtttctcaaccatcgagtctcttggaagctttttggcgggacgttccgccaaaaaagcaaaagtctc
< p15A ori

caagagattacgcgcagaccaaaacgatctcaagaagatcatcttattaatcagataaaatatttctagatttcagtgcaattta   1445
gttctctaatgcgcgtctggttttgctagagttcttctagtagaataattagtctattttataaagatctaaagtcacgttaaat
< p15A ori

tctcttcaaatgtagcacctgaagtcagccccatacgatataagttgtaattctcatgtttgacagcttatcatcgataagcttC   1530
agagaagtttacatcgtggacttcagtcggggtatgctatattcaacattaagagtacaaactgtcgaatagtagctattcgaaG

TGCCGCACAGCTCCATAGGCCGCTTTCCTGGCTTTGCTTCCAGATGTATGCTCTTCTGCTCCCGAACGCCAGCAAGACGTAGCCC   1615
ACGGCGTGTCGAGGTATCCGGCGAAAGGACCGAAACGAAGGTCTACATACGAGAAGACGAGGGCTTGCGGTCGTTCTGCATCGGG

AGCGCGTCGGCCAGCTTGCAATTCGCGCTAACTTACATTAATTGCGTTGCGCTCACTGCCCGCTTTCCAGTCGGGAAACCTGTCG   1700
TCGCGCAGCCGGTCGAACGTTAAGCGCGATTGAATGTAATTAACGCAACGCGAGTGACGGGCGAAAGGTCAGCCCTTTGGACAGC
                                                              360       355
                                                             * Q G S E L R S V Q R
                                                             < lacI

TGCCAGCTGCATTAATGAATCGGCCAACGCGCGGGGAGAGGCGGTTTGCGTATTGGGCGCCAGGGTGGTTTTTCTTTTCACCAGT   1785
ACGGTCGACGTAATTACTTAGCCGGTTGCGCGCCCCTCTCCGCCAAACGCATAACCCGCGGTCCCACCAAAAAGAAAAGTGGTCA
350       345       340       335       330       325
 A L Q M L S D A L A R P S A T Q T N P A L T T K R K V L
< lacI

GAGACGGGCAACAGCTGATTGCCCTTCACCGCCTGGCCCTGAGAGAGTTGCAGCAAGCGGTCCACGCTGGTTTGCCCCAGCAGGC   1870
CTCTGCCCGTTGTCGACTAACGGGAAGTGGCGGACCGGGACTCTCTCAACGTCGTTCGCCAGGTGCGACCAAACGGGGTCGTCCG
  320       315       310       305       300       295
S V P L L Q N G K V A Q G Q S L Q L L R D V S T Q G L L R
< lacI

GAAAATCCTGTTTGATGGTGGTTAACGGCGGGATATAACATGAGCTGTCTTCGGTATCGTCGTATCCCACTACCGAGATATCCGC   1955
CTTTTAGGACAAACTACCACCAATTGCCGCCCTATATTGTACTCGACAGAAGCCATAGCAGCATAGGGTGATGGCTCTATAGGCG
    290       285       280       275       270
 F D Q K I T T L P P I Y C S S D E T D D Y G V V S I D A
< lacI

ACCAACGCGCAGCCCGGACTCGGTAATGGCGCGCATTGCGCCCAGCGCCATCTGATCGTTGGCAACCAGCATCGCAGTGGGAACG   2040
TGGTTGCGCGTCGGGCCTGAGCCATTACCGCGCGTAACGCGGGTCGCGGTAGACTAGCAACCGTTGGTCGTAGCGTCACCCTTGC
265       260       255       250       245       240
 G V R L G S E T I A R M A G L A M Q D N A V L M A T P V
< lacI

ATGCCCTCATTCAGCATTTGCATGGTTTGTTGAAAACCGGACATGGCACTCCAGTCGCCTTCCCGTTCCGCTATCGGCTGAATTT   2125
TACGGGAGTAAGTCGTAAACGTACCAAACAACTTTTGGCCTGTACCGTGAGGTCAGCGGAAGGGCAAGGCGATAGCCGACTTAAA
  235       230       225       220       215       210
I G E N L M Q M T Q Q F G S M A S W D G E R E A I P Q I Q
< lacI
```

```
GATTGCGAGTGAGATATTTATGCCAGCCAGCCAGACGCAGACGCGCCGAGACAGAACTTAATGGGCCCGCTAACAGCGCGATTTG   2210
CTAACGCTCACTCTATAAATACGGTCGGTCGGTCTGCGTCTGCGCGGCTCTGTCTTGAATTACCCGGGCGATTGTCGCGCTAAAC
     205        200        195        190        185
 N  R  T  L  Y  K  H  W  G  A  L  R  L  R  A  S  V  S  S  L  P  G  A  L  L  A  I  Q
< lacI

CTGGTGACCCAATGCGACCAGATGCTCCACGCCCAGTCGCGTACCGTCTTCATGGGAGAAAATAATACTGTTGATGGGTGTCTGG   2295
GACCACTGGGTTACGCTGGTCTACGAGGTGCGGGTCAGCGCATGGCAGAAGTACCCTCTTTTATTATGACAACTACCCACAGACC
180        175        170        165        160        155
 Q  H  G  L  A  V  L  H  E  V  G  L  R  T  G  D  E  H  S  F  I  I  S  N  I  P  T  Q
< lacI

TCAGAGACATCAAGAAATAACGCCGGAACATTAGTGCAGGCAGCTTCCACAGCAATGGCATCCTGGTCATCCAGCGGATAGTTAA   2380
AGTCTCTGTAGTTCTTTATTGCGGCCTTGTAATCACGTCCGTCGAAGGTGTCGTTACCGTAGGACCAGTAGGTCGCCTATCAATT
     150        145        140        135        130        125
 D  S  V  D  L  F  L  A  P  V  N  T  C  A  A  E  V  A  I  A  D  Q  D  D  L  P  Y  N  I
< lacI

TGATCAGCCCACTGACGCGTTGCGCGAGAAGATTGTGCACCGCCGCTTTACAGGCTTCGACGCCGCTTCGTTCTACCATCGACAC   2465
ACTAGTCGGGTGACTGCGCAACGCGCTCTTCTAACACGTGGCGGCGAAATGTCCGAAGCTGCGGCGAAGCAAGATGGTAGCTGTG
     120        115        110        105        100
 I  L  G  S  V  R  Q  A  L  L  N  H  V  A  A  K  C  A  E  V  G  S  R  E  V  M  S  V
< lacI

CACCACGCTGGCACCCAGTTGATCGGCGCGAGATTTAATCGCCGCGACAATTTGCGACGGCGCGTGCAGGGCCAGACTGGAGGTG   2550
GTGGTGCGACCGTGGGTCAACTAGCCGCGCTCTAAATTAGCGGCGCTGTTAAACGCTGCCGCGCACGTCCCGGTCTGACCTCCAC
95         90         85         80         75         70
 V  V  S  A  G  L  Q  D  A  R  S  K  I  A  A  V  I  Q  S  P  A  H  L  A  L  S  S  T
< lacI

GCAACGCCAATCAGCAACGACTGTTTGCCCGCCAGTTGTTGTGCCACGCGGTTGGGAATGTAATTCAGCTCCGCCATCGCCGCTT   2635
CGTTGCGGTTAGTCGTTGCTGACAAACGGGCGGTCAACAACACGGTGCGCCAACCCTTACATTAAGTCGAGGCGGTAGCGGCGAA
     65         60         55         50         45         40
 A  V  G  I  L  L  S  Q  K  G  A  L  Q  Q  A  V  R  N  P  I  Y  N  L  E  A  M  A  A  E
< lacI

CCACTTTTTCCCGCGTTTTCGCAGAAACGTGGCTGGCCTGGTTCACCACGCGGGAAACGGTCTGATAAGAGACACCGGCATACTC   2720
GGTGAAAAAGGGCGCAAAAGCGTCTTTGCACCGACCGGACCAAGTGGTGCGCCCTTTGCCAGACTATTCTCTGTGGCCGTATGAG
     35         30         25         20         15
 V  K  E  R  T  K  A  S  V  H  S  A  Q  N  V  V  R  S  V  T  Q  Y  S  V  G  A  Y  E
< lacI

TGCGACATCGTATAACGTTACTGGTTTCACATTCACCACCCTGAATTGACTCTCTTCCGGGCGCTATCATGCCATACCGCGAAAG   2805
ACGCTGTAGCATATTGCAATGACCAAAGTGTAAGTGGTGGGACTTAACTGAGAGAAGGCCCGCGATAGTACGGTATGGCGCTTTC
10          5        1
 A  V  D  Y  L  T  V  P  K  M
< lacI            < lacIq promoter

GTTTTGCACCATTCGATGGTGTCAACGTAAATGCCGCTTCGCCTTCGCGCGCGAATTGCAAGCTGATCCGGGCTTATCGACTGCA   2890
CAAAACGTGGTAAGCTACCACAGTTGCATTTACGGCGAAGCGGAAGCGCGCGCTTAACGTTCGACTAGGCCCGAATAGCTGACGT
< lacIq promoter

CGGTGCACCAATGCTTCTGGCGTCAGGCAGCCATCGGAAGCTGTGGTATGGCTGTGCAGGTCGTAAATCACTGCATAATTCGTGT   2975
GCCACGTGGTTACGAAGACCGCAGTCCGTCGGTAGCCTTCGACACCATACCGACACGTCCAGCATTTAGTGACGTATTAAGCACA

CGCTCAAGGCGCACTCCCGTTCTGGATAATGTTTTTTGCGCCGACATCATAACGGTTCTGGCAAATATTCTGAAATGAGCTGTTG   3060
GCGAGTTCCGCGTGAGGGCAAGACCTATTACAAAAAACGCGGCTGTAGTATTGCCAAGACCGTTTATAAGACTTTACTCGACAAC
                                                                                 -35 >
                                                                          tac promoter
```

```
ACAATTAATCATCGGCTCGTATAATGTGTGGAATTGTGAGCGGATAACAATTTCACACAGGAAACAgagcacatcagcAGGaGAT   3145
TGTTAATTAGTAGCCGAGCATATTACACACCTTAACACTCGCCTATTGTTAAAGTGTGTCCTTTGTctcgtgtagtcgTCCtCTA
-35 | -10   lac operator
tac promoter

CTAAAGAGGAGAAAGGATCTatgaactacaccgacattcaggcacgtctggcaatcatcaagtcactcccgattagcgaactgga   3230
GATTTCTCCTCTTTCCTAGAtacttgatgtggctgtaagtccgtgcagaccgttagtagttcagtgagggctaatcgcttgacct
1 5 10 15 20
M N Y T D I Q A R L A I I K S L P I S E L D
RBS   JSS1_004

caagcgccagcctcttctggttgcactggctgctgacatcgtgaacggtgagacaagcgatggcaacgatactgatggctcaaat   3315
gttcgcggtcggagaagaccaacgtgaccgacgactgtagcacttgccactctgttcgctaccgttgctatgactaccgagttta
25 30 35 40 45 50
K R Q P L L V A L A A D I V N G E T S D G N D T D G S N
JSS1_004

ggcttggagtatcaagactggtggcacacattgggtgcactgatgcgtgatgcaggtttccgcatgttgggtaatggtcacttta   3400
ccgaacctcatagttctgaccaccgtgtgtaacccacgtgactacgcactacgtccaaaggcgtacaacccattaccagtgaaat
55 60 65 70 75
G L E Y Q D W W H T L G A L M R D A G F R M L G N G H F
JSS1_004

gtgcggcctactcgcatgaactgttaccgggtcgagtcatcaaggttggctttaagaaagaggattcaggcgctgcgtacactgc   3485
cacgccggatgagcgtacttgacaatggcccagctcagtagttccaaccgaaattctttctcctaagtccgcgacgcatgtgacg
80 85 90 95 100 105
S A A Y S H E L L P G R V I K V G F K K E D S G A A Y T A
JSS1_004

gttctgtaggatgcaccaaggccgagcgggtatccctaacgtctatcacgtagcacgacatgcaggttgctatacggtcgtactg   3570
caagacatcctacgtggttccggctcgcccatagggattgcagatagtgcatcgtgctgtacgtccaacgatatgccagcatgac
110 115 120 125 130 135
F C R M H Q G R A G I P N V Y H V A R H A G C Y T V V L
JSS1_004

gaccgcttacaaagctgtgaccgctatgctaacgatgtgcacgcaaagtatgcaagtgctgcgcaggagttcattgagtgcactg   3655
ctggcgaatgtttcgacactggcgatacgattgctacacgtgcgtttcatacgttcacgacgcgtcctcaagtaactcacgtgac
140 145 150 155 160
D R L Q S C D R Y A N D V H A K Y A S A A Q E F I E C T
JSS1_004

gaggtgatgctgattggtacgcacagtatgttagcaatgagttcattgagacgtgcaagatgattcgtgagttcttccacggtat   3740
ctccactacgactaaccatgcgtgtcatacaatcgttactcaagtaactctgcacgttctactaagcactcaagaaggtgccata
165 170 175 180 185 190
G G D A D W Y A Q Y V S N E F I E T C K M I R E F F H G I
JSS1_004

tgcatctttcgacatgcacagcgggaacattatgttcgatgataacgatgtcccgtacatcactgacccggttagtttctcacat   3825
acgtagaaagctgtacgtgtcgcccttgtaatacaagctactattgctacagggcatgtagtgactgggccaatcaaagagtgta
195 200 205 210 215 220
A S F D M H S G N I M F D D N D V P Y I T D P V S F S H
JSS1_004

gaccgtgaacgtgaggacggttttcctctggaccctgaagcactgctggctgaggtcgaggctgtagcgcaagaacgcatgattg   3910
ctggcacttgcactcctgccaaaaggagacctgggacttcgtgacgaccgactccagctccgacatcgcgttcttgcgtactaac
225 230 235 240 245
D R E R E D G F P L D P E A L L A E V E A V A Q E R M I
JSS1_004
```

```
aacggtgccgcaaccgcaaggccaagtgtgaccctaacggaacctttcaggttaaccgcaaggccgcaatgaaacgccgtaagcg  3995
ttgccacggcgttggcgttccggttcacactgggattgccttggaaagtccaattggcgttccggcgttactttgcggcattcgc
  250            255            260            265            270            275
E  R  C  R  N  R  K  A  K  C  D  P  N  G  T  F  Q  V  N  R  K  A  A  M  K  R  R  K  R
JSS1_004

caaccgcaagttacgcgccaaggtagctgaacgtgaccgcttacacttcatggcaatccgtagggaacgcggtgtgattgagcgt  4080
gttggcgttcaatgcgcggttccatcgacttgcactggcgaatgtgaagtaccgttaggcatcccttgcgccacactaactcgca
     280            285            290            295            300            305
 N  R  K  L  R  A  K  V  A  E  R  D  R  L  H  F  M  A  I  R  R  E  R  G  V  I  E  R
JSS1_004

aacgaacgccgtgctgagatgctcatggggtctgcatggcatgacttctggttacgcaatggtaacgcaacggttcgcaagattg  4165
ttgcttgcggcacgactctacgagtaccccagacgtaccgtactgaagaccaatgcgttaccattgcgttgccaagcgttctaac
          310            315            320            325            330
 N  E  R  R  A  E  M  L  M  G  S  A  W  H  D  F  W  L  R  N  G  N  A  T  V  R  K  I
JSS1_004

accaagtgaacggccttaagtggcaactaggggaccgcctcgcaatacaagcgggtctccctttgaacatcgacaaggtacttga  4250
tggttcacttgccggaattcaccgttgatcccctggcggagcgttatgttcgcccagagggaaacttgtagctgttccatgaact
  335            340            345            350            355            360
D  Q  V  N  G  L  K  W  Q  L  G  D  R  L  A  I  Q  A  G  L  P  L  N  I  D  K  V  L  D
JSS1_004

cgctcacctgatgggctaggatcctctacgccggacgcatcgtggccggcatcaccggcgccacaggtgcggttgctggcgccta  4335
gcgagtggactacccgatcctaggagatgcggcctgcgtagcaccggccgtagtggccgcggtgtccacgccaacgaccgcggat
     365
 A  H  L  M  G  *
JSS1_004

tatcgccgacatcaccgatggggaagatcgggctcgccacttcgggctcatgagcgcttgtttcggcgtgggtatggtggcaggc  4420
atagcggctgtagtggctaccccttctagcccgagcggtgaagcccgagtactcgcgaacaaagccgcacccataccaccgtccg

cccgtggccgggggactgttgggcgccatctccttgcatgcaccattccttgcggcggcggtgctcaacggcctcaacctactac  4505
gggcaccggccccctgacaacccgcggtagaggaacgtacgtggtaaggaacgccgccgccacgagttgccggagttggatgatg

tgggctgcttcctaatgcaggagtcgcataagggagagcgtcgaccgatgcccttgagagccttcaacccagtcagctccttccg  4590
acccgacgaaggattacgtcctcagcgtattccctctcgcagctggctacgggaactctcggaagttgggtcagtcgaggaaggc

gtgggcgcggggcatgactatcgtcgccgcacttatgactgtcttctttatcatgcaactcgtaggacaggtgccggcagcgctc  4675
cacccgcgccccgtactgatagcagcggcgtgaatactgacagaagaaatagtacgttgagcatcctgtccacggccgtcgcgag

tgggtcattttcggcgaggaccgctttcgctggagcgcgacgatgatcggcctgtcgcttgcggtattcggaatcttgcacgccc  4760
acccagtaaaagccgctcctggcgaaagcgacctcgcgctgctactagccggacagcgaacgccataagccttagaacgtgcggg

tcgctcaagccttcgtcactggtcccgccaccaaacgtttcggcgagaagcaggccattatcgccggcatggcggccgacgcgct  4845
agcgagttcggaagcagtgaccagggcggtggtttgcaaagccgctcttcgtccggtaatagcggccgtaccgccggctgcgcga

gggctacgtcttgctggcgttcgcgacgcgaggctggatggccttccccattatgattcttctcgcttccggcggcatcgggatg  4930
cccgatgcagaacgaccgcaagcgctgcgctccgacctaccggaaggggtaatactaagaagagcgaaggccgccgtagccctac

cccgcgttgcaggccatgctgtccaggcaggtagatgacgaccatcagggacagcttcaaggatcgctcgcggctcttaccagcc  5015
gggcgcaacgtccggtacgacaggtccgtccatctactgctggtagtccctgtcgaagttcctagcgagcgccgagaatggtcgg

taacttcgatcactggaccgctgatcgtcacggcgatttatgccgcctcggcgagcacatggaacgggttggcatggattgtagg  5100
attgaagctagtgacctggcgactagcagtgccgctaaatacggcggagccgctcgtgtaccttgcccaaccgtacctaacatcc
```

```
cgccgccctataccttgtctgcctccccgcgttgcgtcgcggtgcatggagccgggccacctcgacctgaatggaagccggcggc
                                                                                           5185
gcggcgggatatggaacagacggaggggcgcaacgcagcgccacgtacctcggcccggtggagctggacttaccttcggccgccg

acctcgctaacggattcaccactccaagaattggagccaatcaattcttgcggagaactgtgaatgcgcaaaccaacccttggca
                                                                                           5270
tggagcgattgcctaagtggtgaggttcttaacctcggttagttaagaacgcctcttgacacttacgcgtttggttgggaaccgt

gaacatatccatcgcgtccgccatctccagcagccgcacgcggcgcatctcgggcagcgttgggtcctggccacgggtgcgcatg
                                                                                           5355
cttgtataggtagcgcaggcggtagaggtcgtcggcgtgcgccgcgtagagcccgtcgcaacccaggaccggtgcccacgcgtac

atcgtgctcctgtcgttgaggacccggctaggctggcggggttgccttactggttagcagaatgaatcaccgatacgcgagcgaa
                                                                                           5440
tagcacgaggacagcaactcctgggccgatccgaccgccccaacggaatgaccaatcgtcttacttagtggctatgcgctcgctt

cgtgaagcgactgctgctgcaaaacgtctgcgacctgagcaacaacatgaatggtcttcggtttccgtgtttcgtaaagtctgga
                                                                                           5525
gcacttcgctgacgacgacgttttgcagacgctggactcgttgttgtacttaccagaagccaaaggcacaaagcatttcagacct

aacgcggaagtcccctacgtgctgctgaagttgcccgcaacagagagtggaaccaaccggtgataccacgatactatgactgaga
                                                                                           5610
ttgcgccttcaggggatgcacgacgacttcaacgggcgttgtctctcaccttggttggccactatggtgctatgatactgactct

gtcaacgccatgagcggcctcatttcttattctgagttacaacagtccgcaccgctgtccggtagctccttccggtgggcgcggg
                                                                                           5695
cagttgcggtactcgccggagtaaagaataagactcaatgttgtcaggcgtggcgacaggccatcgaggaaggccacccgcgccc

gcatgactatcgtcgccgcacttatgactgtcttctttatcatgcaactcgtaggacaggtgccggcagcgcccaacagtccccc
                                                                                           5780
cgtactgatagcagcggcgtgaatactgacagaagaaatagtacgttgagcatcctgtccacggccgtcgcgggttgtcaggggg

ggccacggggcctgccaccatacccacgccgaaacaagcgccctgcaccattatgttccggatctgcatcgcaggatgctgctgg
                                                                                           5865
ccggtgccccggacggtggtatgggtgcggctttgttcgcgggacgtggtaatacaaggcctagacgtagcgtcctacgacgacc

ctaccctgtggaacacctacatctgtattaacgaagcgctaaccgtttttatcaggctctgggaggcagaataaatgatcatatc
                                                                                           5950
gatgggacaccttgtggatgtagacataattgcttcgcgattggcaaaaatagtccgagaccctccgtcttatttactagtatag

gtcaattattacctccacggggagagcctgagcaaactggcctcaggcatttgagaagcacacggtcacactgcttccggtagtc
                                                                                           6035
cagttaataatggaggtgcccctctcggactcgtttgaccggagtccgtaaactcttcgtgtgccagtgtgacgaaggccatcag

aataaaccggtaaaccagcaatagacataagcggctatttaacgaccctgccctgaaccgacgaccgggtcgaatttgctttcga
                                                                                           6120
ttatttggccatttggtcgttatctgtattcgccgataaattgctgggacgggacttggctgctggcccagcttaaacgaaagct

atttctgccattcatccgcttattatcacttattcaggcgtagcaccaggcgtttaagggcaccaataactgccttaaaaaaatt
                                                                                           6205
taaagacggtaagtaggcgaataatagtgaataagtccgcatcgtggtccgcaaattcccgtggttattgacggaattttttttaa
                                                                                    220
                                                                                     *
                                                                                   CmR

acgccccgccctgccactcatcgcagtactgttgtaattcattaagcattctgccgacatggaagccatcacagacggcatgatg
                                                                                           6290
tgcggggcgggacggtgagtagcgtcatgacaacattaagtaattcgtaagacggctgtaccttcggtagtgtctgccgtactac
           215              210              205              200              195
  A  G  G  Q  W  E  D  C  Y  Q  Q  L  E  N  L  M  R  G  V  H  F  G  D  C  V  A  H  H
<                                       CmR

aacctgaatcgccagcggcatcagcaccttgtcgccttgcgtataatatttgcccatggtgaaaacgggggcgaagaagttgtcc
                                                                                           6375
ttggacttagcggtcgccgtagtcgtggaacagcggaacgcatattataaacgggtaccacttttgcccccgcttcttcaacagg
   190              185              180              175              170              165
 V  Q  I  A  L  P  M  L  V  K  D  G  Q  T  Y  Y  K  G  M  T  F  V  P  A  F  F  N  D
<                                       CmR
```

```
atattggccacgtttaaatcaaaactggtgaaactcacccagggattggctgagacgaaaaacatattctcaataaaccctttag
                                                                                      6460
tataaccggtgcaaatttagttttgaccactttgagtgggtccctaaccgactctgctttttgtataagagttatttgggaaatc
    160        155        150        145        140        135
M  N  A  V  N  L  D  F  S  T  F  S  V  W  P  N  A  S  V  F  F  M  N  E  I  F  G  K  P
<                                  CmR

ggaaataggccaggttttcaccgtaacacgccacatcttgcgaatatatgtgtagaaactgccggaaatcgtcgtggtattcact
                                                                                      6545
cctttatccggtccaaaagtggcattgtgcggtgtagaacgcttatatacacatctttgacggcctttagcagcaccataagtga
         130        125        120        115        110
 F  Y  A  L  N  E  G  Y  C  A  V  D  Q  S  Y  I  H  L  F  Q  R  F  D  D  H  Y  E  S
<                                  CmR

ccagagcgatgaaaacgtttcagtttgctcatggaaaacggtgtaacaagggtgaacactatcccatatcaccagctcaccgtct
                                                                                      6630
ggtctcgctactttgcaaagtcaaacgagtaccttttgccacattgttcccacttgtgatagggtatagtggtcgagtggcaga
  105        100        95        90        85        80
W  L  S  S  F  T  E  T  Q  E  H  F  V  T  Y  C  P  H  V  S  D  W  I  V  L  E  G  D
<                                  CmR

ttcattgccatacg     3'
               •••   6644
aagtaacggtatgc     5'
      75
K  M  A  M  R
<   CmR
```

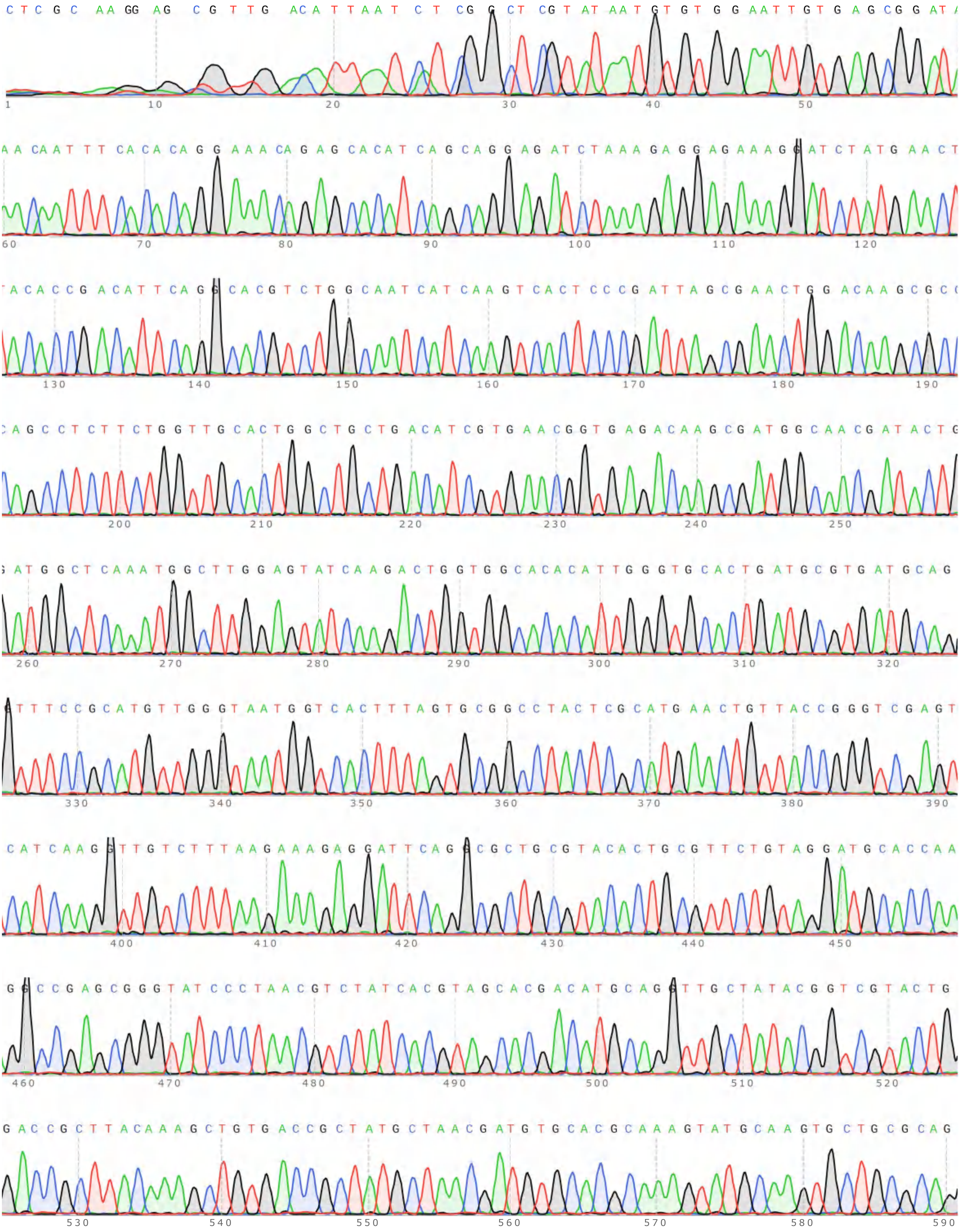

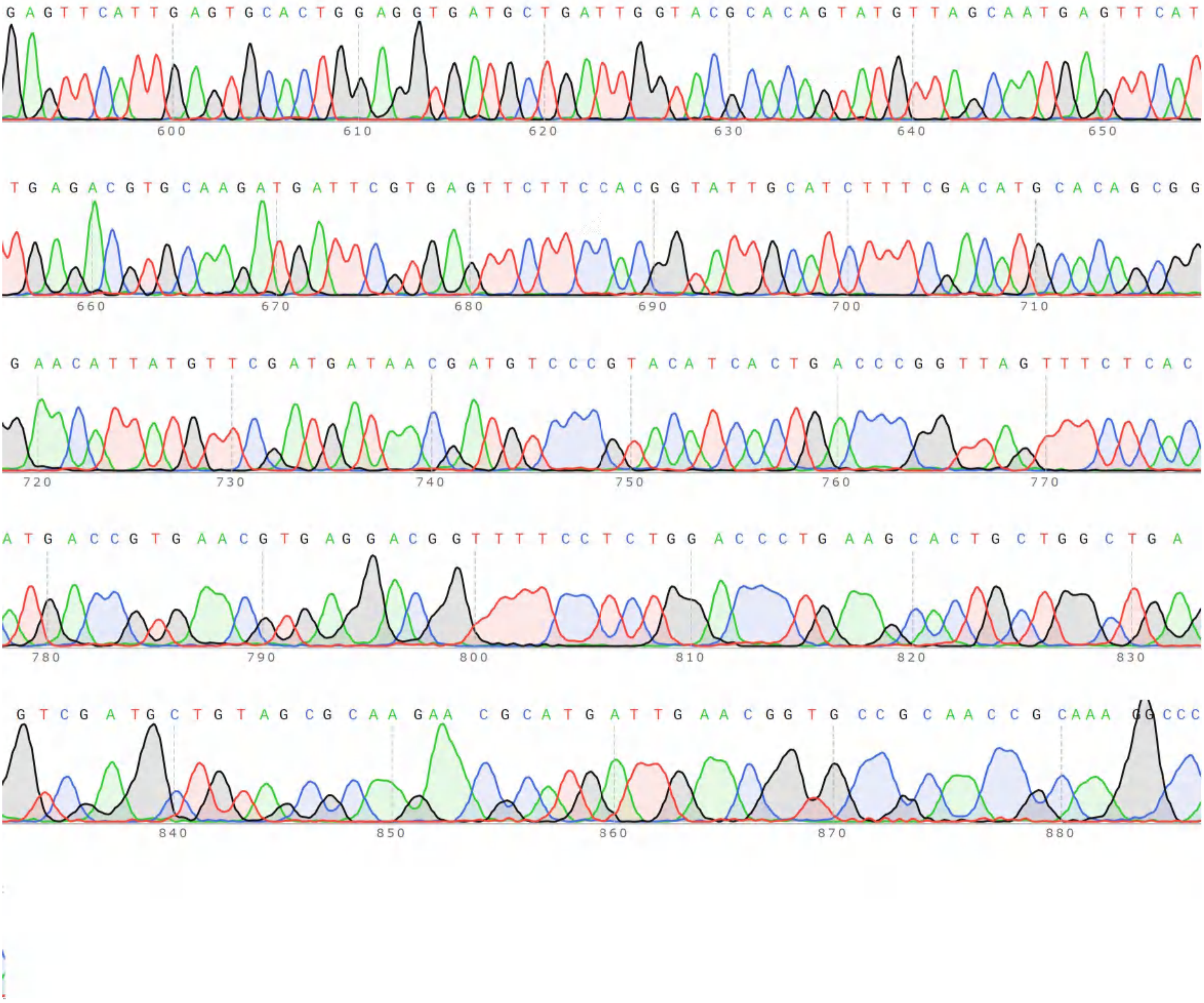
G A G T T C A T T G A G T G C A C T G G A G G T G A T G C T G A T T G G T A C G C A C A G T A T G T T A G C A A T G A G T T C A T
600 610 620 630 640 650
T G A G A C G T G C A A G A T G A T T C G T G A G T T C T T C C A C G G T A T T G C A T C T T T C G A C A T G C A C A G C G G
660 670 680 690 700 710
G A A C A T T A T G T T C G A T G A T A A C G A T G T C C C G T A C A T C A C T G A C C C G G T T A G T T T C T C A C
720 730 740 750 760 770
A T G A C C G T G A A C G T G A G G A C G G T T T T C C T C T G G A C C C T G A A G C A C T G C T G G C T G A
780 790 800 810 820 830
G T C G A T G C T G T A G C G C A A G A A C G C A T G A T T G A A C G G T G C C G C A A C C G C A A A G G C C C
840 850 860 870 880

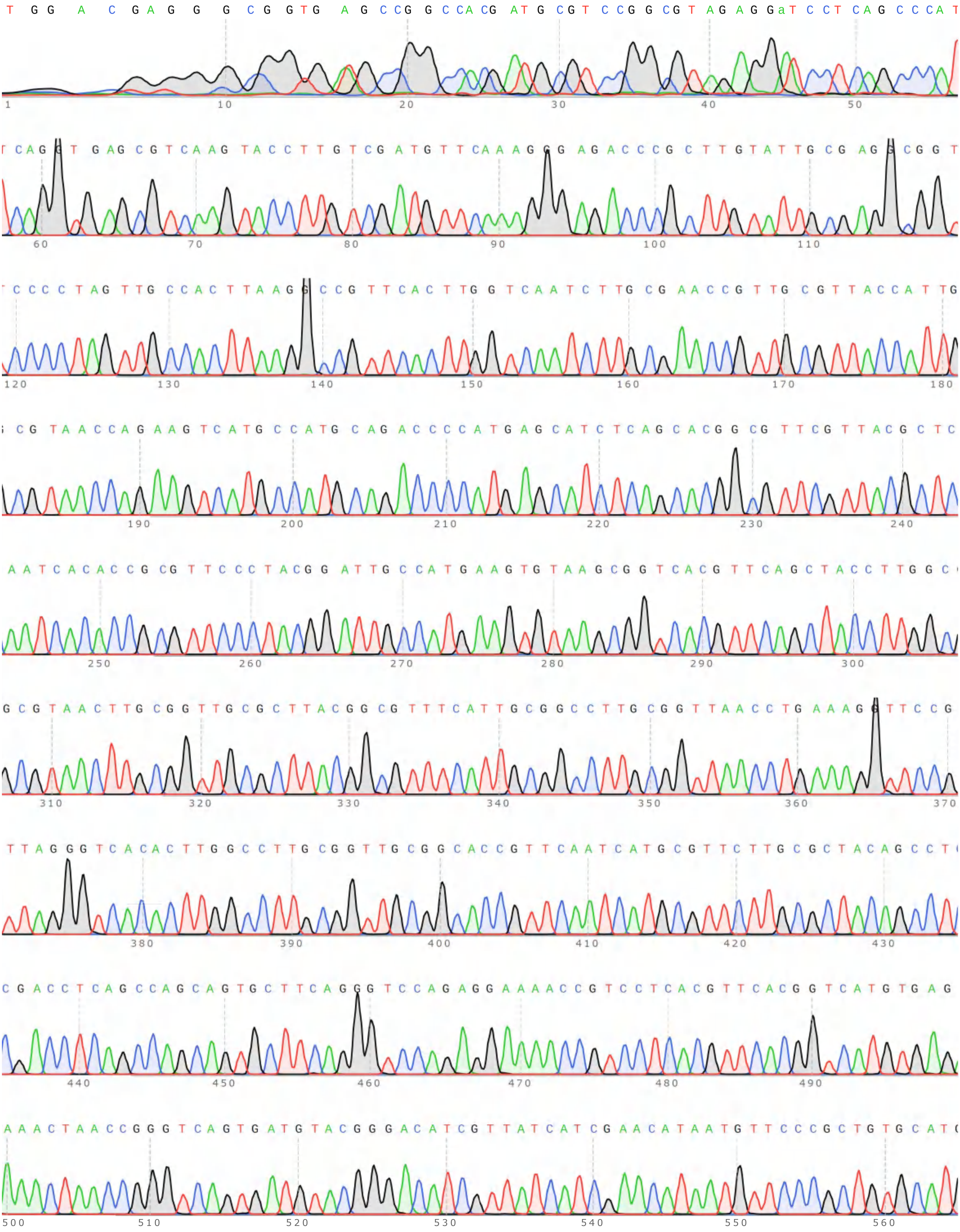
T GG A C GAG G G CG GTG AGCCG GCCACG ATG CGT CCGG CGT AGAGGaT CCT CAG CCCA T
T CAGGT GAG CGTCAAG TACCTTGTCG ATGTTCAAAGGG AGACCCG CTTGTATTG CG AGG CGGT
T CCCTAG TTG CCACTTAAGG CCG TTCACTTG G TCAATCTTG CG AACCG TTG CGTTACCATTG
CG TAACCAGAAGTCATG CCATG CAG ACCCCATGAGCATCTCAGCACGG CG TTCGTTACGCTC
AATCACACCG CG TTCCCTACGG ATTG CCATGAAGTGTAAGCGGTCACG TTCAGCTACCTTGGC
GCGTAACTTG CGGTTGCGCTTACGG CG TTTCATTGCGGCCTTG CGGTTAACCTG AAAGGTTCCG
TTAGGGTCACACTTGGCCTTGCGGTTGCGGCACCGTTCAATCATGCGTTCTTGCGCTACAGCCT
CGACCTCAGCCAGCAGTGCTTCAGGGTCCAGAGGAAAACCGTCCTCACGTTCACGGTCATGTGAG
AAACTAACCGGGTCAGTGATGTACGGGACATCGTTATCATCGAACATAATGTTCCCGCTGTGCAT

G T C G A A A G A T G C A A T A C C G T G G A A G A A C T C A C G A A T C A T C T T G C A C G T C T C A A T G A A C T C A T T G C T

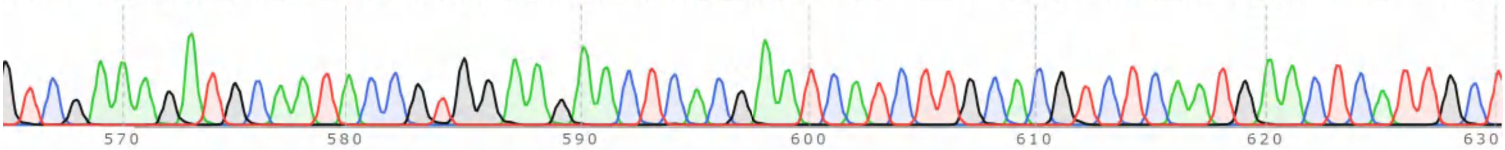

T A A C A T A C T G T G C G T A C C A A T C A G C A T C A C C T C C A G T G C A C T C A A T G A A C T C C T G C G C A G C A C T T G

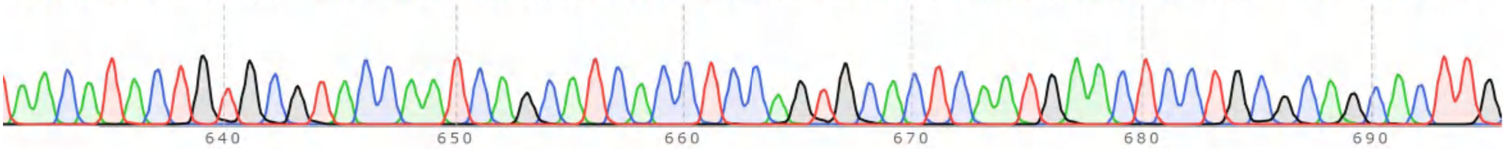

C A T A C T T T G C G T G C A C A T C G T T A G C A T A G C G G T C A C A G C T T T G T A A G C G G T C C A G T A C G A C C G T A

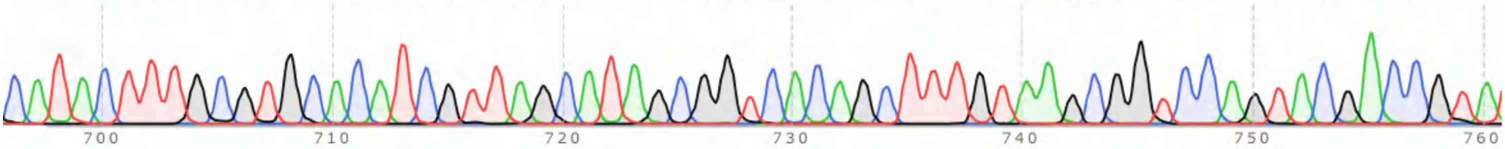

T A G C A A C C T G C A T G T C G T G C T A C G T G A T A G A C G T T A G G G A T A C C C G C T C G G C C T T G G T G C A T C C T

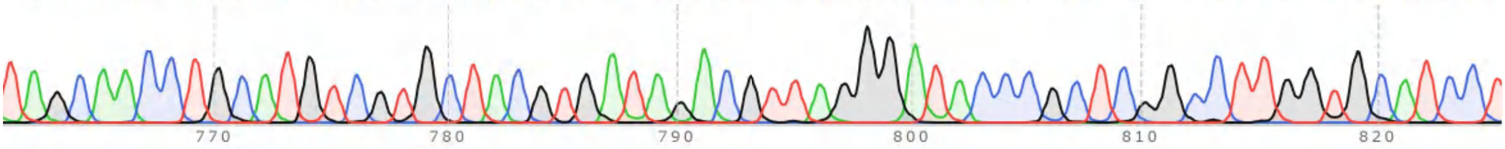

A C A G A A C G C A G T G T A C G C A G C G C C T G A A T C C T C T T T C T T A A A G A C A A C C T T G A T G A C T C G A C C C

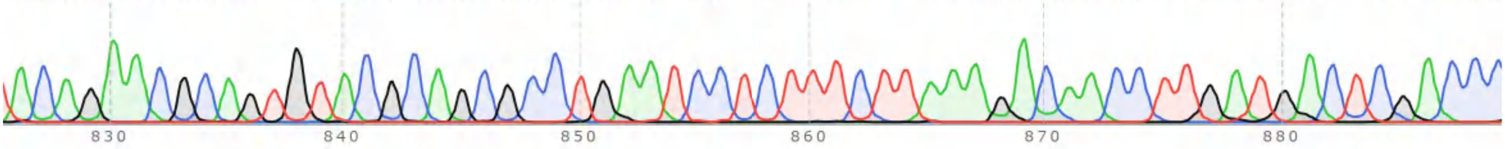

G G T A A C A G T T C A T G C G A G T A G G C C G C A C T A A A G T G A C C A T T A C C C A A C A T G C G G A A A C C T G C

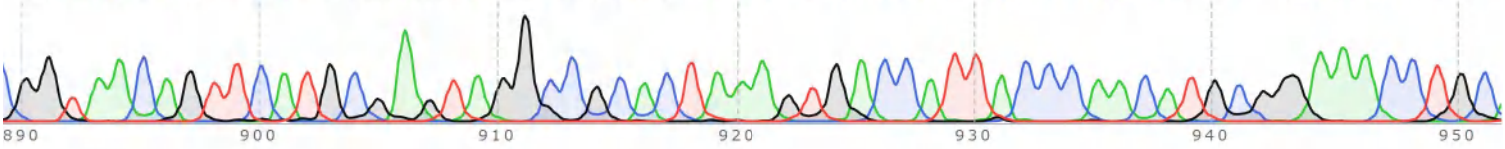

A T C A C G C A T C A G T G C A C C

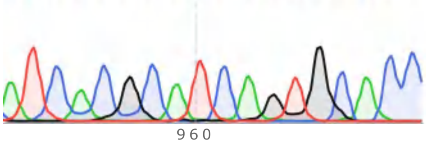

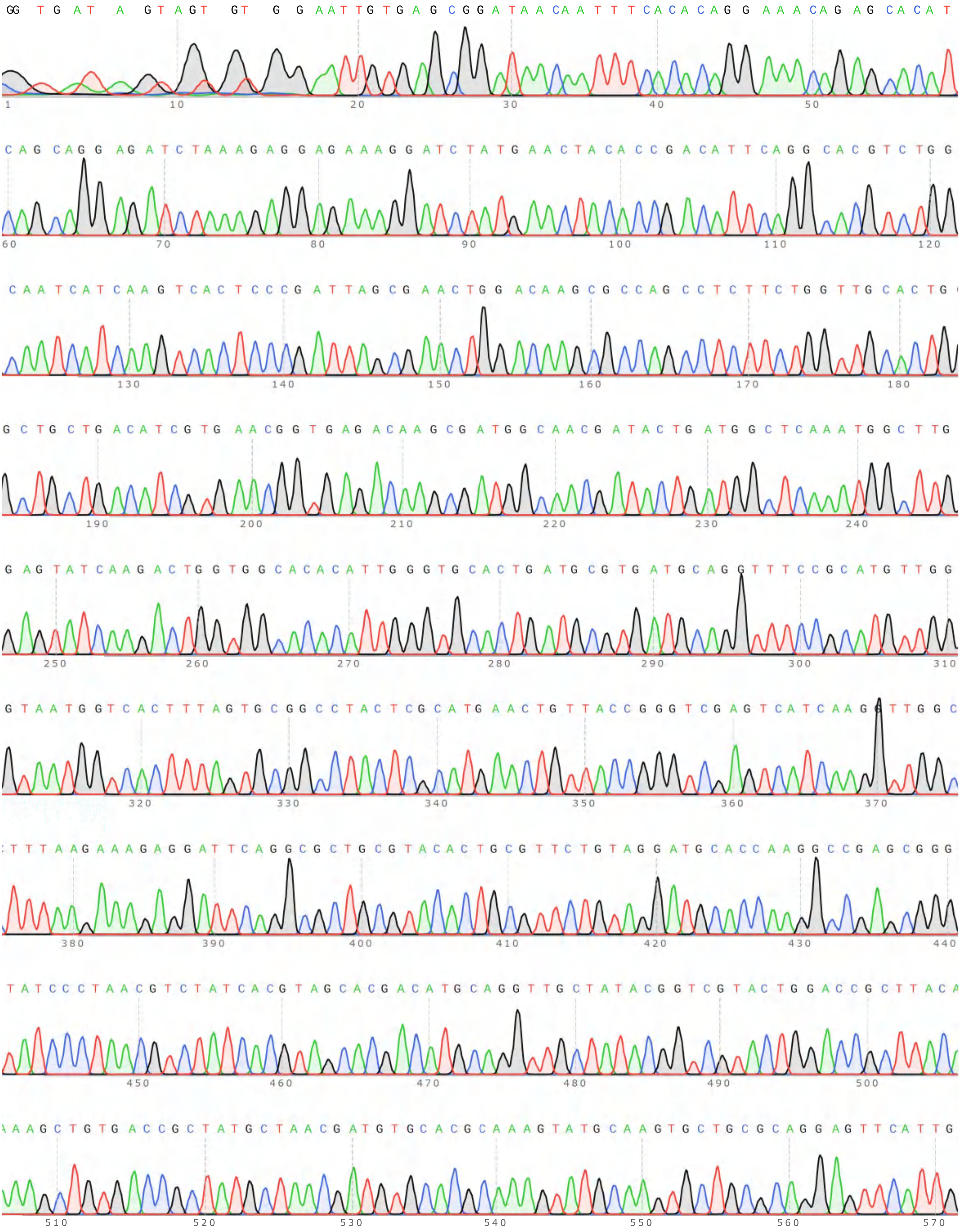
GG T G A T A G T A G T G T G G AATTGTGAGCGGATAACAATTTCACACAGG AAACAG AG CACAT
CAG CAGG AGATCTAAAGAGGAGAAAGGATCTATGAACTACACCGACATTCAGG CACGTCTGG
CAATCATCAAGTCACTCCCGATTAGCGAACTGGACAAGCGCCAGCCTCTTCTGGTTGCACTG
GCTGCTGACATCGTGAACGGTGAGACAAGCGATGGCAACGATACTGATGGCTCAAATGGCTTG
GAGTATCAAGACTGGTGGCACACATTGGGTGCACTGATGCGTGATGCAGGTTTCCGCATGTTGG
GTAATGGTCACTTTAGTGCGGCCTACTCGCATGAACTGTTACCGGGTCGAGTCATCAAGGTTGGC
TTTAAGAAAGAGGATTCAGGCGCTGCGTACACTGCGTTCTGTAGGATGCACCAAGGCCGAGCGGG
TATCCCTAACGTCTATCACGTAGCACGACATGCAGGTTGCTATACGGTCGTACTGGACCGCTTACA
AAAGCTGTGACCGCTATGCTAACGATGTGCACGCAAAGTATGCAAGTGCTGCGCAGGAGTTCATTG

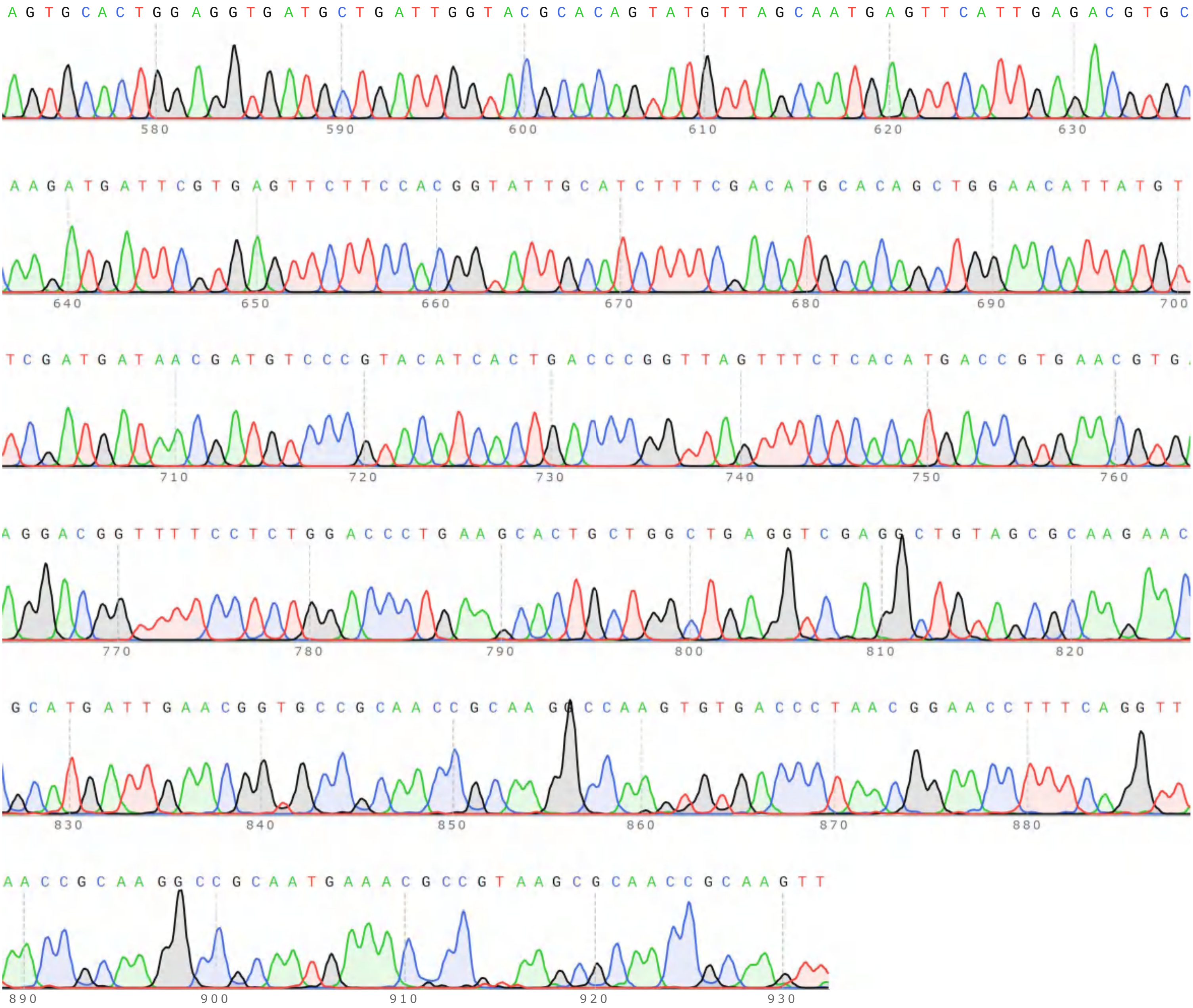
AGTGCACTGGAGGTGATGCTGATTGGTACGCACAGTATGTTAGCAATGAGTTCATTGAGACGTGC
580 590 600 610 620 630
AAGATGATTCGTGAGTTCTTCCACGGTATTGCATCTTTCGACATGCACAGCTGGAACATTATGT
640 650 660 670 680 690 700
TCGATGATAACGATGTCCCGTACATCACTGACCCGGTTAGTTTCTCACATGACCGTGAACGTG
710 720 730 740 750 760
AGGACGGTTTTCCTCTGGACCCTGAAGCACTGCTGGCTGAGGTCGAGGCTGTAGCGCAAGAAC
770 780 790 800 810 820
GCATGATTGAACGGTGCCGCAACCGCAAGGCCAAGTGTGACCCTAACGGAACCTTTCAGGTT
830 840 850 860 870 880
AACCGCAAGGCCGCAATGAAACGCCGTAAGCGCAACCGCAAGTT
890 900 910 920 930

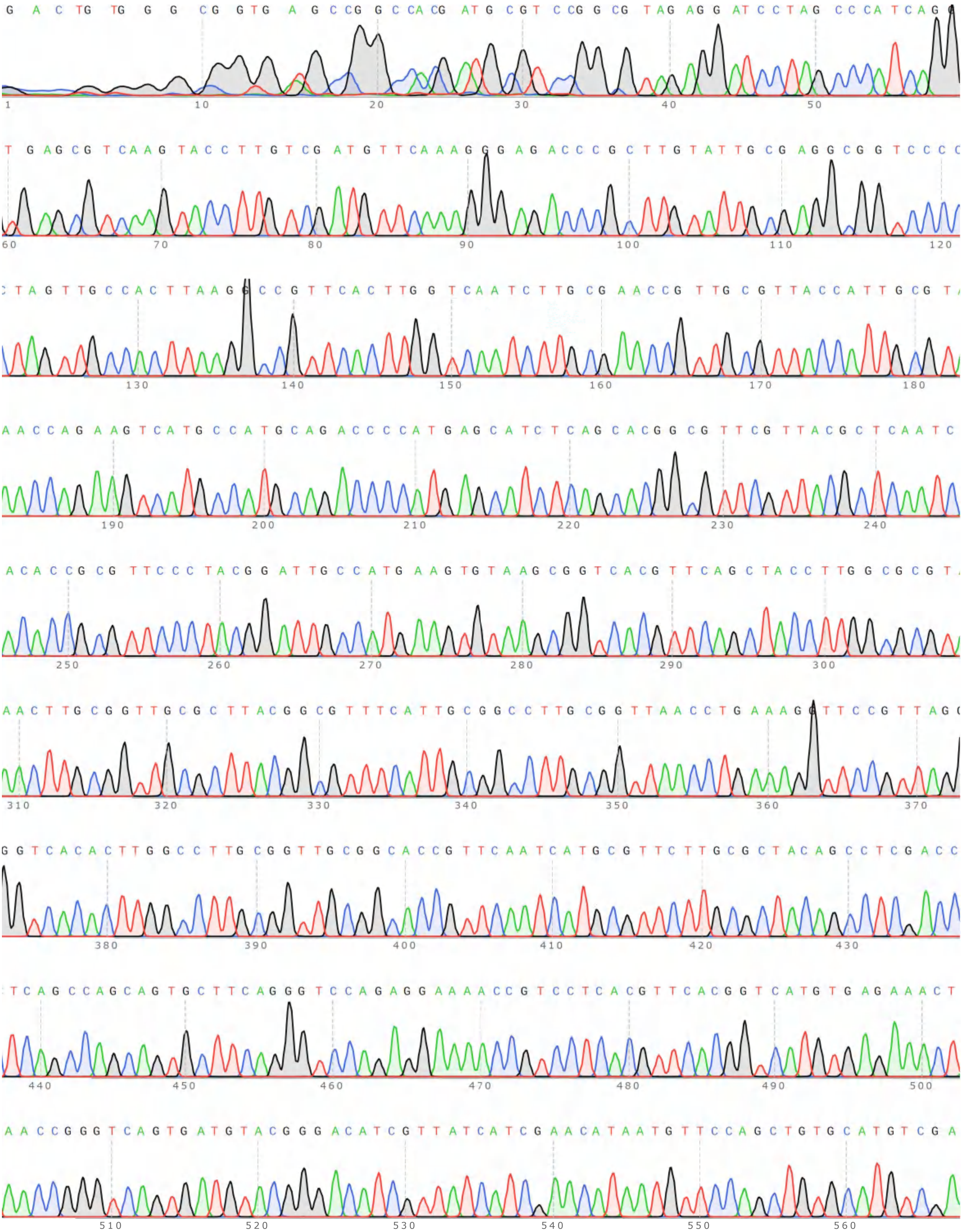

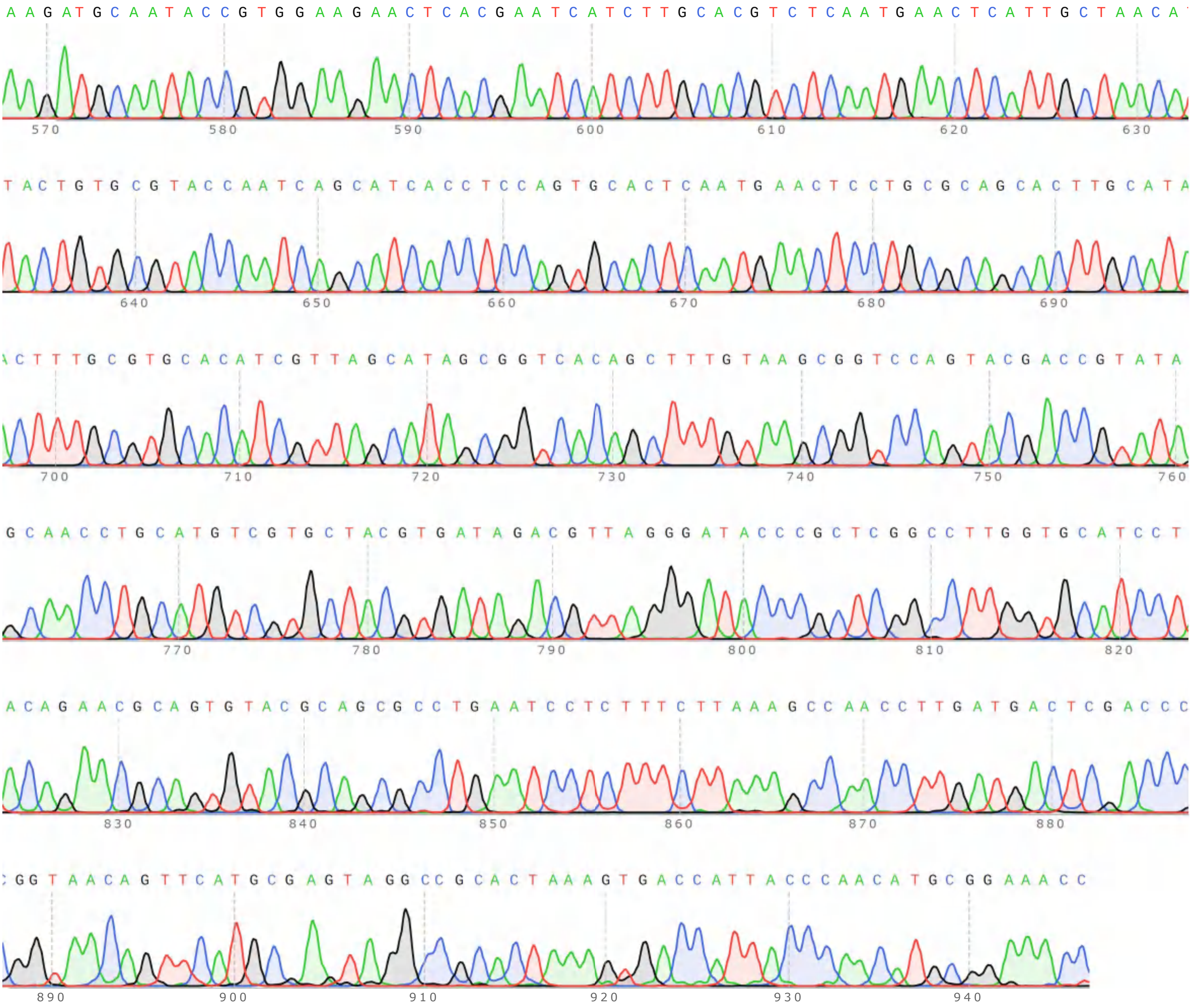
AAGATGCAATACCGTGGAAGAACTCACGAATCATCTTGCACGTCTCAATGAACTCATTGCTAACA
570 580 590 600 610 620 630
TACTGTGCGTACCAATCAGCATCACCTCCAGTGCACTCAATGAACTCCTGCGCAGCACTTGCATA
640 650 660 670 680 690
ACTTTGCGTGCACATCGTTAGCATAGCGGTCACAGCTTTGTAAGCGGTCCAGTACGACCGTATA
700 710 720 730 740 750 760
GCAACCTGCATGTCGTGCTACGTGATAGACGTTAGGGATACCCGCTCGGCCTTGGTGCATCCT
770 780 790 800 810 820
ACAGAACGCAGTGTACGCAGCGCCTGAATCCTCTTTCTTAAAGCCAACCTTGATGACTCGACCC
830 840 850 860 870 880
GGTAACAGTTCATGCGAGTAGGCCGCACTAAAGTGACCATTACCCAACATGCGGAAACC
890 900 910 920 930 940

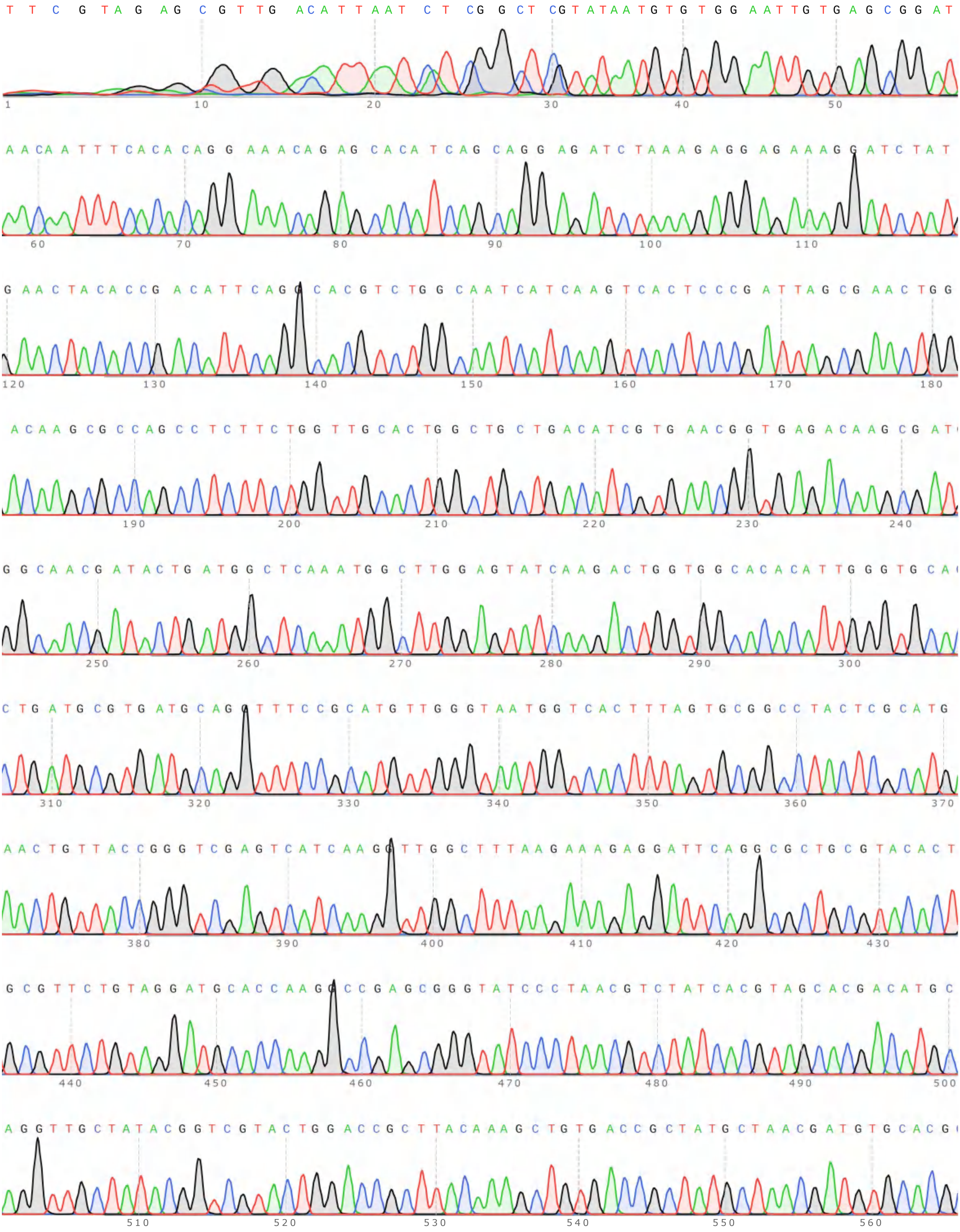
T T C G T A G A G C G T T G A C A T T A A T C T C G G C T C G T A T A A T G T G T G G A A T T G T G A G C G G A T
A A C A A T T T C A C A C A G G A A A C A G A G C A C A T C A G C A G G A G A T C T A A A G A G G A G A A A G G A T C T A T
G A A C T A C A C C G A C A T T C A G G C A C G T C T G G C A A T C A T C A A G T C A C T C C C G A T T A G C G A A C T G G
A C A A G C G C C A G C C T C T T C T G G T T G C A C T G G C T G C T G A C A T C G T G A A C G G T G A G A C A A G C G A T
G G C A A C G A T A C T G A T G G C T C A A A T G G C T T G G A G T A T C A A G A C T G G T G G C A C A C A T T G G G T G C A
C T G A T G C G T G A T G C A G G T T T C C G C A T G T T G G G T A A T G G T C A C T T T A G T G C G G C C T A C T C G C A T G
A A C T G T T A C C G G G T C G A G T C A T C A A G G T T G G C T T T A A G A A A G A G G A T T C A G G C G C T G C G T A C A C T
G C G T T C T G T A G G A T G C A C C A A G G C C G A G C G G G T A T C C C T A A C G T C T A T C A C G T A G C A C G A C A T G C
A G G T T G C T A T A C G G T C G T A C T G G A C C G C T T A C A A A G C T G T G A C C G C T A T G C T A A C G A T G T G C A C G

C A A A G T A T G C A A G T G C T G C G C A G G A G T T C A T T G A G T G C A C T G G A G G T G A T G C T G A T T G G T A C G C A C

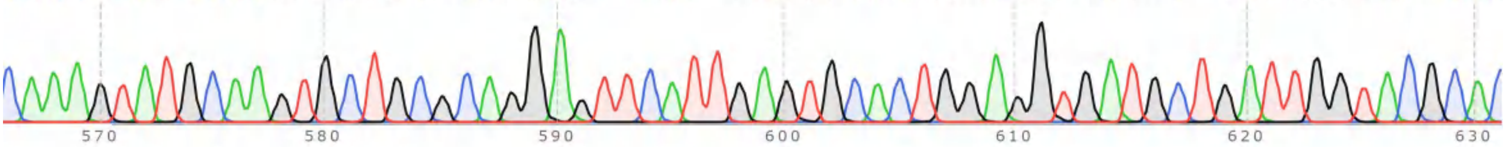

C A G T A T G T T A G C A A T G A G T T C A T T G A G A C G T G C A A G A T G A T T C G T G A G T T C T T C C A C G G T A T T G C A

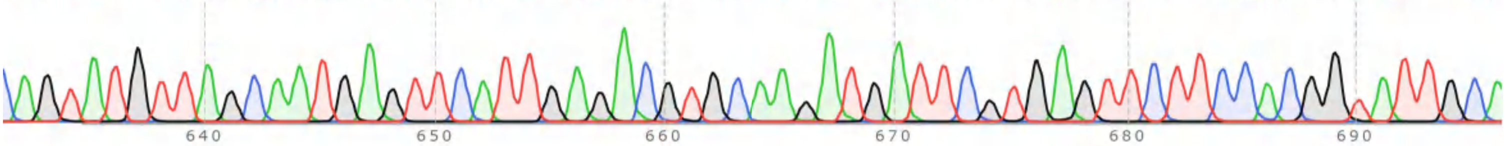

T C T T T C G A C A T G C A C A G C G G G A A C A T T A C G T T C G A T G A T A A C G A T G T C C C G T A C A T C A C T G A C C

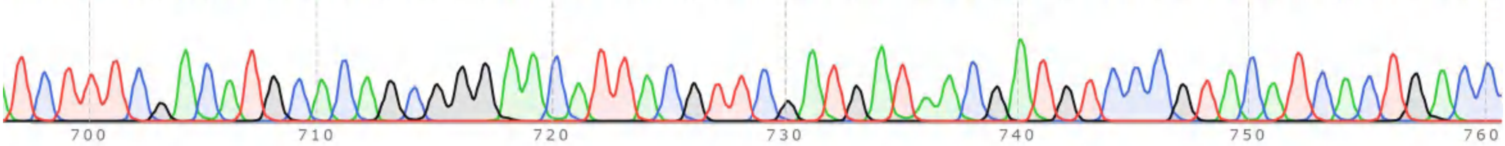

C G G T T A G T T T C T C A C A T G A C C G T G A A C G T G A G G A C G G T T T T C C T C T G G A C C C T G A A G C A C T G C T

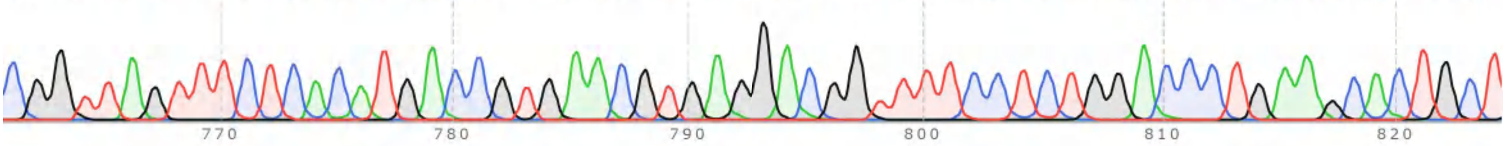

G G C T G A G G T C G A G G C T G T A G C G C A A G A A C G C A T G A T T G A A C G G T G C C G C A A C C G C A A G G C C A A

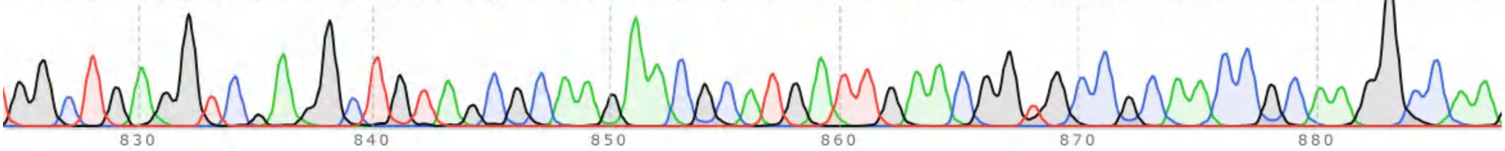

G T G T G A C C C T A A C G G A A C C T T T C A G G T T A A C C G C A A G G C C G C A A T G A A A C G C C G T A A G C G C A A C

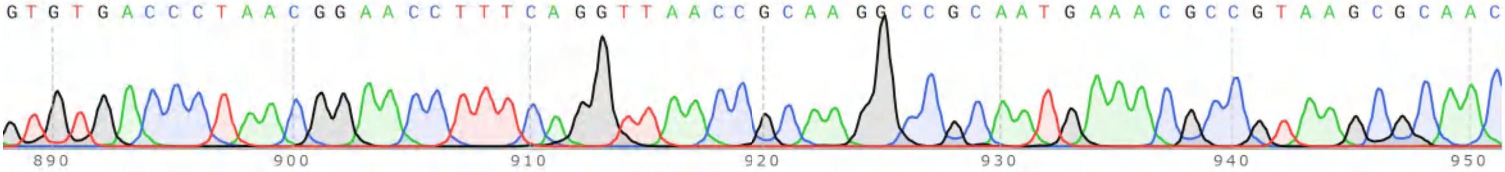

C G C A A G T T A C G C G C C A A G G T A G C T G A A C G T G A

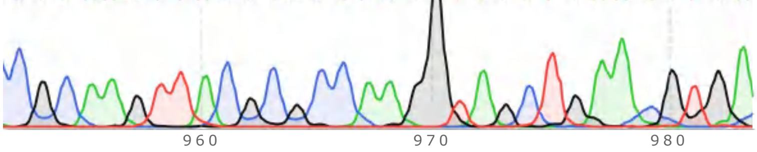

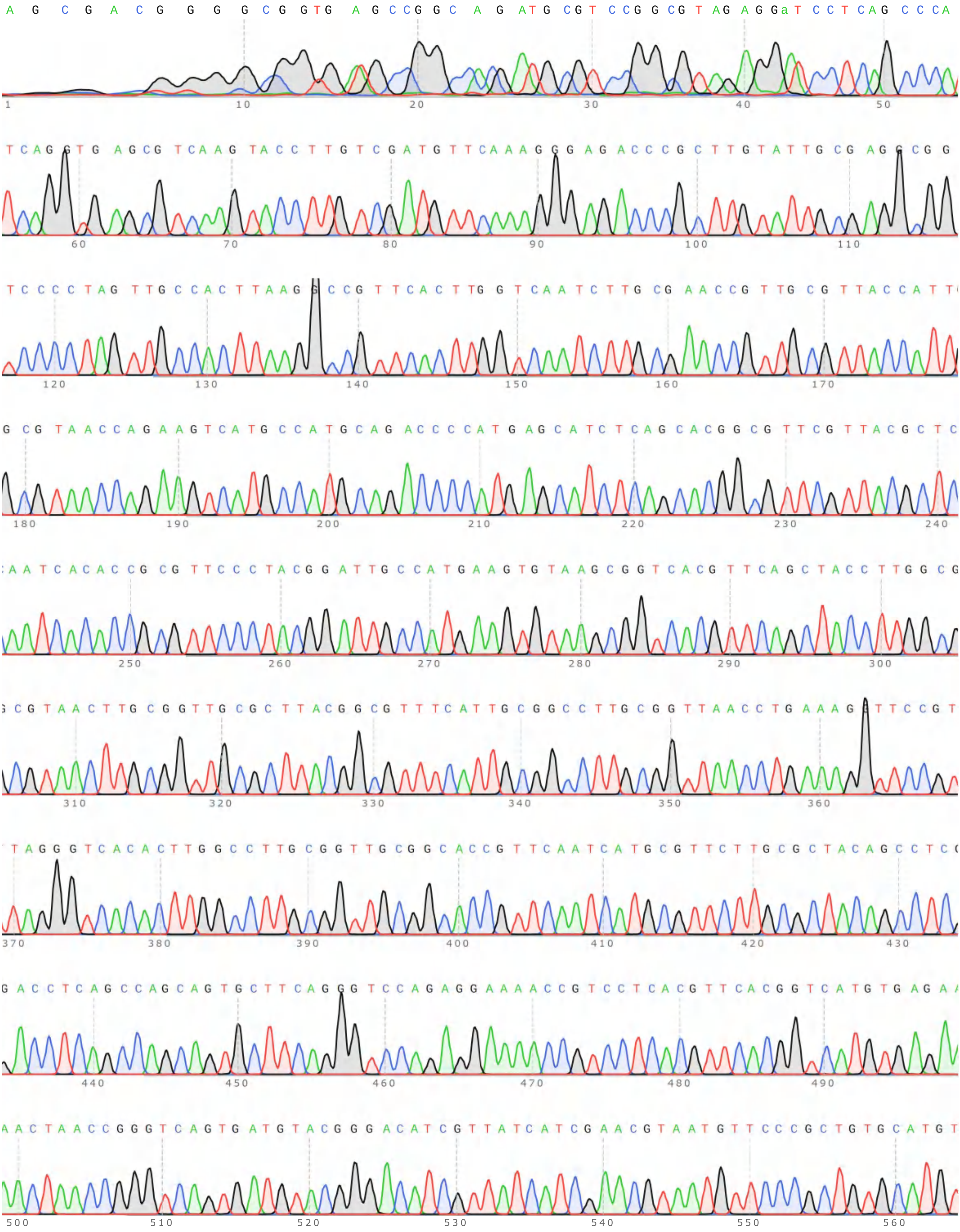
A G C G A C G G G G C G G T G A G C C G G C A G A T G C G T C C G G C G T A G A G G a T C C T C A G C C C A
1 10 20 30 40 50
T C A G G T G A G C G T C A A G T A C C T T G T C G A T G T T C A A A G G G A G A C C C G C T T G T A T T G C G A G G C G G
60 70 80 90 100 110
T C C C C T A G T T G C C A C T T A A G G C C G T T C A C T T G G T C A A T C T T G C G A A C C G T T G C G T T A C C A T T
120 130 140 150 160 170
G C G T A A C C A G A A G T C A T G C C A T G C A G A C C C C A T G A G C A T C T C A G C A C G G C G T T C G T T A C G C T C
180 190 200 210 220 230 240
A A T C A C A C C G C G T T C C C T A C G G A T T G C C A T G A A G T G T A A G C G G T C A C G T T C A G C T A C C T T G G C G
250 260 270 280 290 300
C G T A A C T T G C G G T T G C G C T T A C G G C G T T T C A T T G C G G C C T T G C G G T T A A C C T G A A A G G T T C C G T
310 320 330 340 350 360
T A G G G T C A C A C T T G G C C T T G C G G T T G C G G C A C C G T T C A A T C A T G C G T T C T T G C G C T A C A G C C T C C
370 380 390 400 410 420 430
G A C C T C A G C C A G C A G T G C T T C A G G G T C C A G A G G A A A A C C G T C C T C A C G T T C A C G G T C A T G T G A G A A
440 450 460 470 480 490
A A C T A A C C G G G T C A G T G A T G T A C G G G A C A T C G T T A T C A T C G A A C G T A A T G T T C C C G C T G T G C A T G T
500 510 520 530 540 550 560

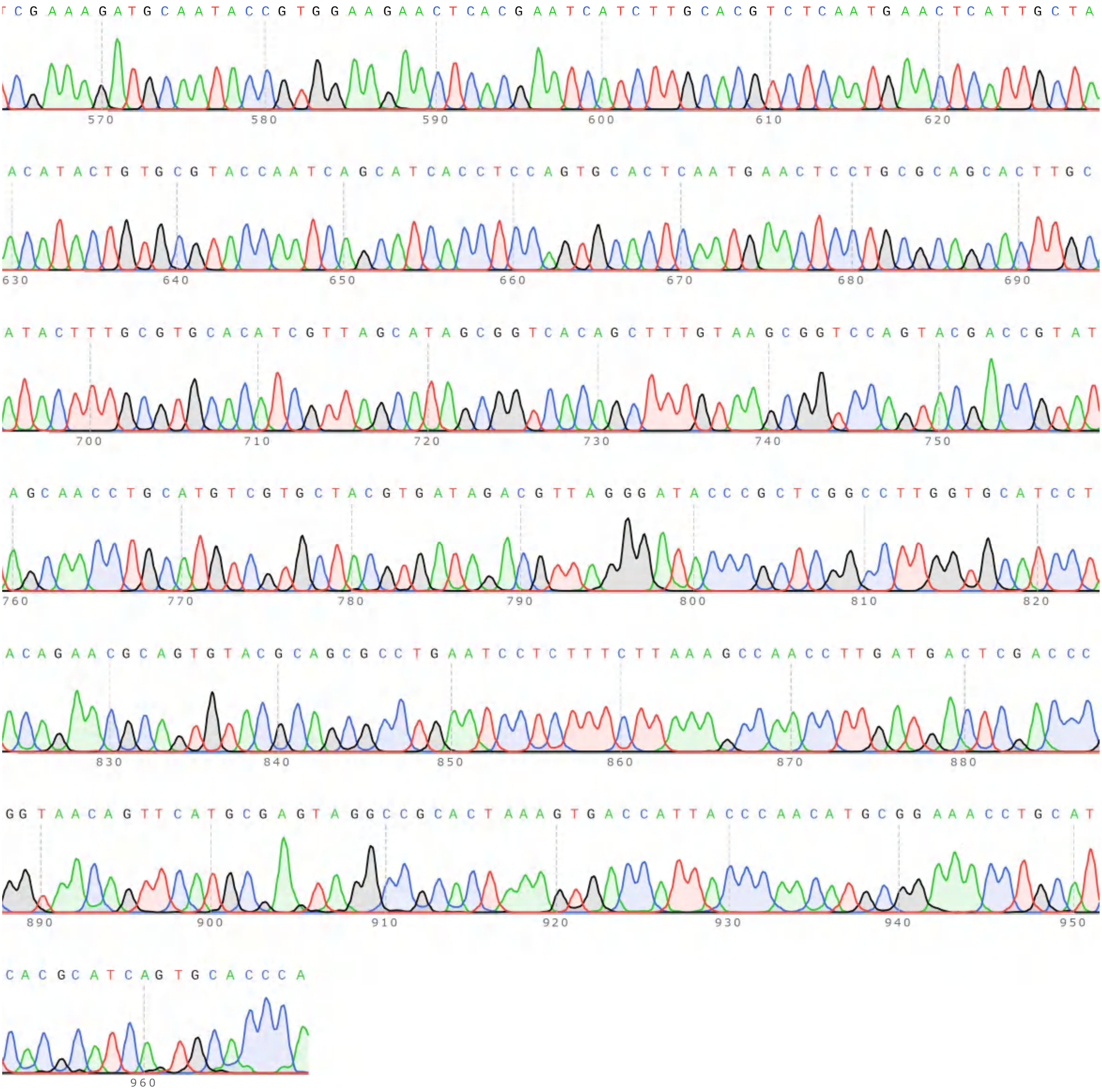
TCGAAAGATGCAATACCGTGGAAGAACTCACGAATCATCTTGCACGTCTCAATGAACTCATTGCTA
570 580 590 600 610 620
ACATACTGTGCGTACCAATCAGCATCACCTCCAGTGCACTCAATGAACTCCTGCGCAGCACTTGC
630 640 650 660 670 680 690
ATACTTTGCGTGCACATCGTTAGCATAGCGGTCACAGCTTTGTAAGCGGTCCAGTACGACCGTAT
700 710 720 730 740 750
AGCAACCTGCATGTCGTGCTACGTGATAGACGTTAGGGATACCCGCTCGGCCTTGGTGCATCCT
760 770 780 790 800 810 820
ACAGAACGCAGTGTACGCAGCGCCTGAATCCTCTTTCTTAAAGCCAACCTTGATGACTCGACCC
830 840 850 860 870 880
GGTAACAGTTCATGCGAGTAGGCCGCACTAAAGTGACCATTACCCAACATGCGGAAACCTGCAT
890 900 910 920 930 940 950
CACGCATCAGTGCACCCA
960

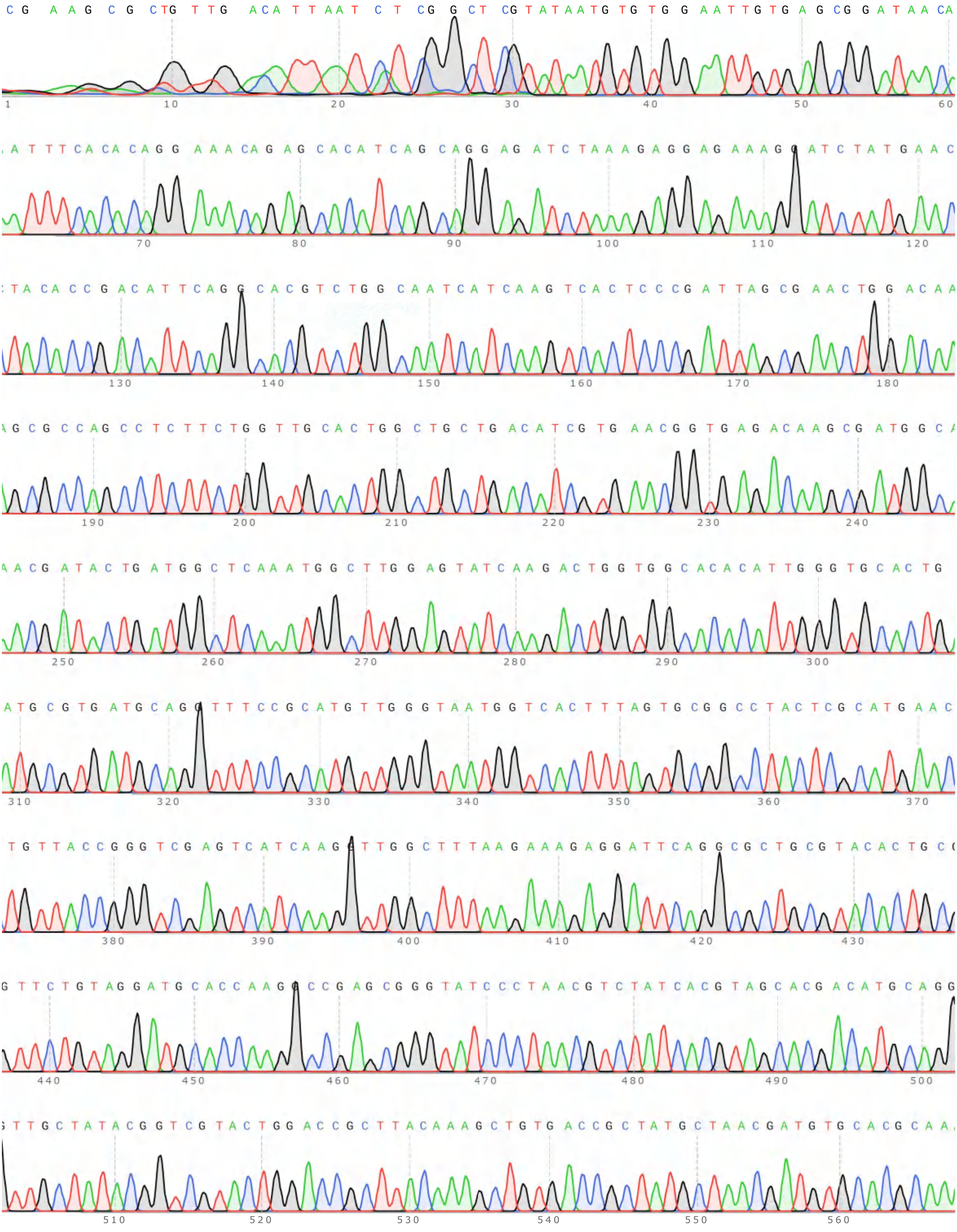

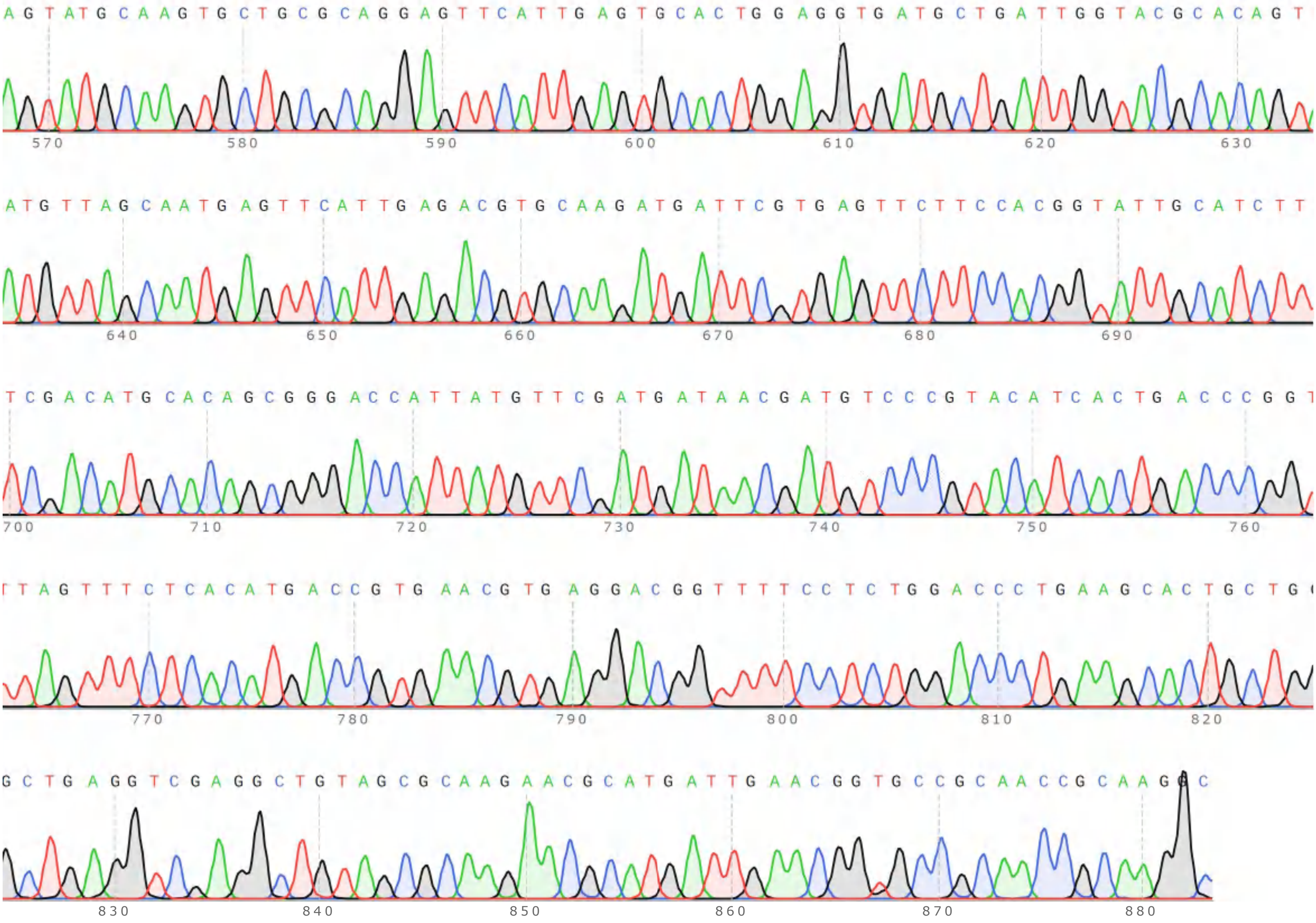
AGTATGCAAGTGCTGCGCAGGAGTTCATTGAGTGCACTGGAGGTGATGCTGATTGGTACGCACAGT
570 580 590 600 610 620 630
ATGTTAGCAATGAGTTCATTGAGACGTGCAAGATGATTCGTGAGTTCTTCCACGGTATTGCATCTT
640 650 660 670 680 690
TCGACATGCACAGCGGGACCATTATGTTCGATGATAACGATGTCCCGTACATCACTGACCCGGT
700 710 720 730 740 750 760
TTAGTTTCTCACATGACCGTGAACGTGAGGACGGTTTTCCTCTGGACCCTGAAGCACTGCTG
770 780 790 800 810 820
GCTGAGGTCGAGGCTGTAGCGCAAGAACGCATGATTGAACGGTGCCGCAACCGCAAGGC
830 840 850 860 870 880

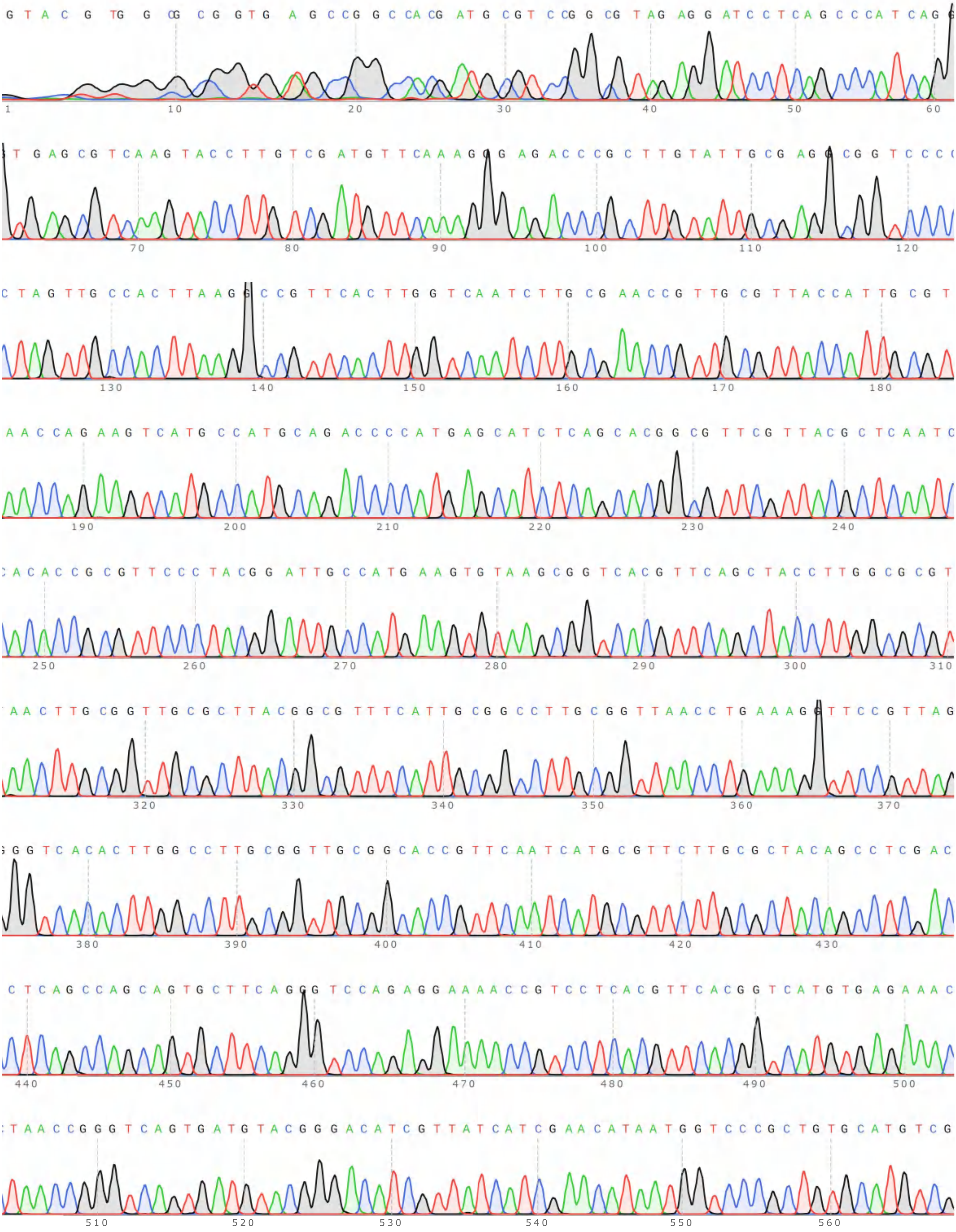

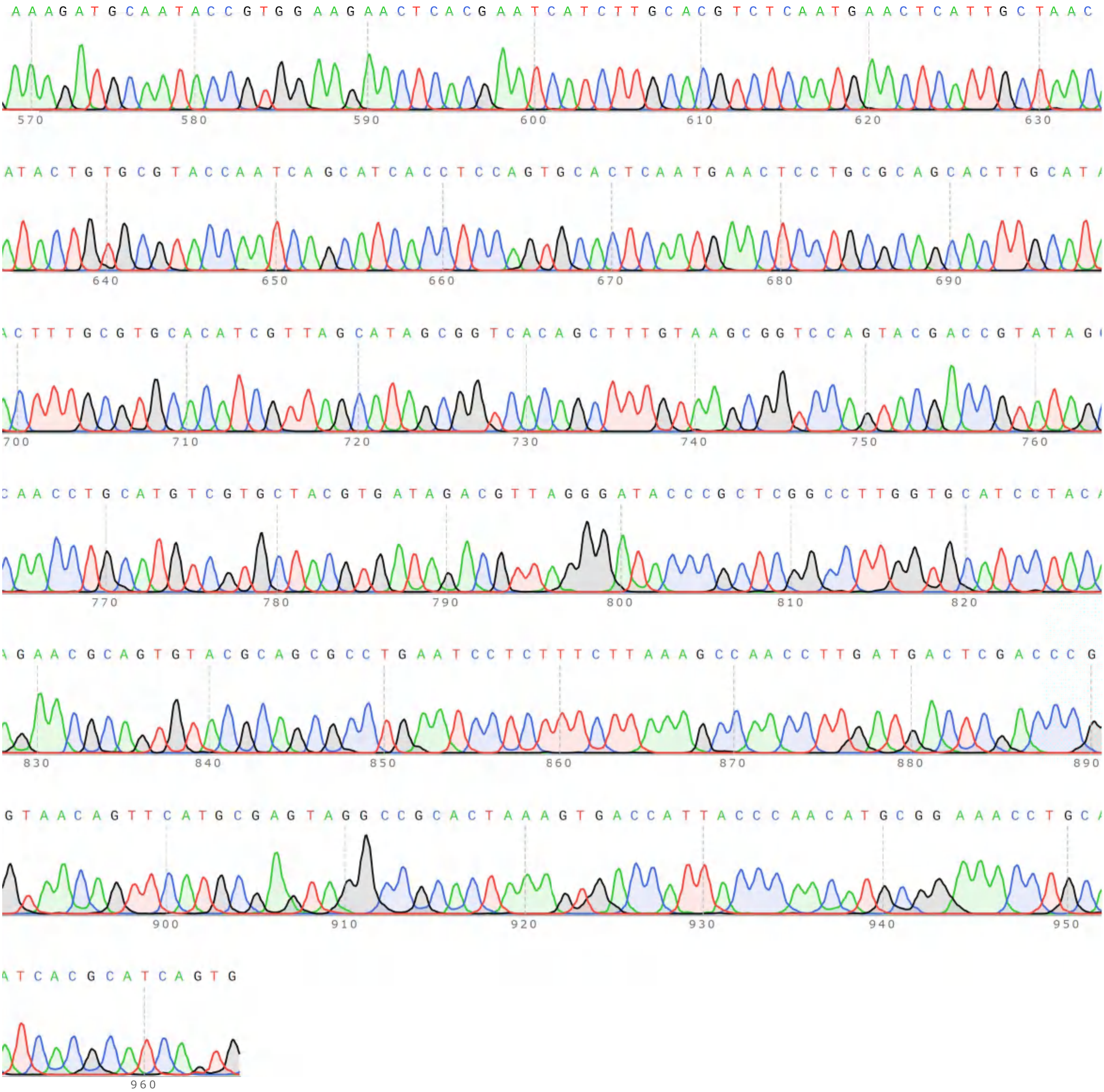

Data Files S1 The sequencing peak map of the JSS1_004 mutant strain plasmid after 5 generations of passage. To evaluate plasmid stability, *Salmonella enterica serovar* Cerro 87 (*dndBCDE-FGH*) and XTG103 (*ΔdndBCDE-FGH*) strains harboring JSS1_004 mutant plasmids were cultured at 28 °C with serial passaging (1:500 dilution every 12 hours) for five generations (G1-G5). Plasmid DNA extracted from G5 cultures underwent Sanger sequence, reconfirming the absence of secondary mutations across coding and regulatory regions and validating the genetic stability of engineered JSS1_004 variants under prolonged cultivation. The sequencing peak map of the fifth-generation JSS1_004 mutant strain plasmid and the theoretical sequence of the wild-type JSS1_004 plasmid are provided above.
