# Supplementary material for: Mechanistic insights into JSS1_004-mediated antagonism of the DndBCDE-FGH restriction system and engineering applications
Source: mBio. 2025 Jul 14;16(8):e01386-25. doi: 10.1128/mbio.01386-25 (PMC12345140; doi:10.1128/mbio.01386-25)
Supplement: File S2 — Sequencing results of recombinant phages after wild-type JSS1_004 was integrated into the M13 genome. [file mbio.01386-25-s0008.pdf]

```
AACGCTACTACTATTAGTAGAATTGATGCCACCTTTTCAGCTCGCGCCCCAAATGAAAATATAGCTAAACAGGTTATTGACCATT 85
TGCGAAATGTATCTAATGGTCAAACTAAATCTACTCGTTCGCAGAATTGGGAATCAACTGTTACATGGAATGAAACTTCCAGACA 170
CCGTACTTTAGTTGCATATTTAAAACATGTTGAGCTACAGCACCAGATTCAGCAATTAAGCTCTAAGCCATCCGCAAAAATGACC 255
TCTTATCAAAAGGAGCAATTAAAGGTACTCTCTAATCCTGACCTGTTGGAGTTTGCTTCCGGTCTGGTTCGCTTTGAAGCTCGAA 340
TTAAAACGCGATATTTGAAGTCTTTCGGGCTTCCTCTTAATCTTTTTGATGCAATCCGCTTTGCTTCTGACTATAATAGTCAGGG 425
TAAAGACCTGATTTTTGATTTATGGTCATTCTCGTTTTCTGAACTGTTTAAAGCATTTGAGGGGGATTCAATGAATATTTATGAC 510
GATTCCGCAGTATTGGACGCTATCCAGTCTAAACATTTTACTATTACCCCCTCTGGCAAAACTTCTTTTGCAAAAGCCTCTCGCT 595
ATTTTGGTTTTTATCGTCGTCTGGTAAACGAGGGTTATGATAGTGTTGCTCTTACTATGCCTCGTAATTCCTTTTGGCGTTATGT 680
ATCTGCATTAGTTGAATGTGGTATTCCTAAATCTCAACTGATGAATCTTTCTACCTGTAATAATGTTGTTCCGTTAGTTCGTTTT 765
ATTAACGTAGATTTTTCTTCCCAACGTCCTGACTGGTATAATGAGCCAGTTCTTAAAATCGCATAAGGTAATTCACAATGATTAA 850
AGTTGAAATTAAACCATCTCAAGCCCAATTTACTACTCGTTCTGGTGTTTCTCGTCAGGGCAAGCCTTATTCACTGAATGAGCAG 935
CTTTGTTACGTTGATTTGGGTAATGAATATCCGGTTCTTGTCAAGATTACTCTTGATGAAGGTCAGCCAGCCTATGCGCCTGGTC 1020
TGTACACCGTTCATCTGTCCTCTTTCAAAGTTGGTCAGTTCGGTTCCCTTATGATTGACCGTCTGCGCCTCGTTCCGGCTAAGTA 1105
ACATGGAGCAGGTCGCGGATTTCGACACAATTTATCAGGCGATGATACAAATCTCCGTTGTACTTTGTTTCGCGCTTGGTATAAT 1190
CGCTGGGGGTCAAAGATGAGTGTTTTAGTGTATTCTTTCGCCTCTTTCGTTTTAGGTTGGTGCCTTCGTAGTGGCATTACGTATT 1275
TTACCCGTTTAATGGAAACTTCCTCATGAAAAAGTCTTTAGTCCTCAAAGCCTCTGTAGCCGTTGCTACCCTCGTTCCGATGCTG 1360
TCTTTCGCTGCTGAGGGTGACGATCCCGCAAAAGCGGCCTTTAACTCCCTGCAAGCCTCAGCGACCGAATATATCGGTTATGCGT 1445
GGGCGATGGTTGTTGTCATTGTCGGCGCAACTATCGGTATCAAGCTGTTTAAGAAATTCACCTCGAAAGCAAGCTGATAAACCGA 1530
TACAATTAAAGGCTCCTTTTGGAGCCTTTTTTTTTGGAGATTTTCAACATGAAAAAATTATTATTCGCAATTCCTTTAGTTGTTC 1615
CTTTCTATTCTCACTCCGCTGAAACTGTTGAAAGTTGTTTAGCAAAACCCCATACAGAAAATTCATTTACTAACGTCTGGAAAGA 1700
CGACAAAACTTTAGATCGTTACGCTAACTATGAGGGTTGTCTGTGGAATGCTACAGGCGTTGTAGTTTGTACTGGTGACGAAACT 1785
CAGTGTTACGGTACATGGGTTCCTATTGGGCTTGCTATCCCTGAAAATGAGGGTGGTGGCTCTGAGGGTGGCGGTTCTGAGGGTG 1870
GCGGTTCTGAGGGTGGCGGTACTAAACCTCCTGAGTACGGTGATACACCTATTCCGGGCTATACTTATATCAACCCTCTCGACGG 1955
CACTTATCCGCCTGGTACTGAGCAAAACCCCGCTAATCCTAATCCTTCTCTTGAGGAGTCTCAGCCTCTTAATACTTTCATGTTT 2040
CAGAATAATAGGTTCCGAAATAGGCAGGGGGCATTAACTGTTTATACGGGCACTGTTACTCAAGGCACTGACCCCGTTAAAACTT 2125
ATTACCAGTACACTCCTGTATCATCAAAAGCCATGTATGACGCTTACTGGAACGGTAAATTCAGAGACTGCGCTTTCCATTCTGG 2210
CTTTAATGAGGATCCATTCGTTTGTGAATATCAAGGCCAATCGTCTGACCTGCCTCAACCTCCTGTCAATGCTGGCGGCGGCTCT 2295
GGTGGTGGTTCTGGTGGCGGCTCTGAGGGTGGTGGCTCTGAGGGTGGCGGTTCTGAGGGTGGCGGCTCTGAGGGAGGCGGTTCCG 2380
GTGGTGGCTCTGGTTCCGGTGATTTTGATTATGAAAAGATGGCAAACGCTAATAAGGGGGCTATGACCGAAAATGCCGATGAAAA 2465
CGCGCTACAGTCTGACGCTAAAGGCAAACTTGATTCTGTCGCTACTGATTACGGTGCTGCTATCGATGGTTTCATTGGTGACGTT 2550
TCCGGCCTTGCTAATGGTAATGGTGCTACTGGTGATTTTGCTGGCTCTAATTCCCAAATGGCTCAAGTCGGTGACGGTGATAATT 2635
CACCTTTAATGAATAATTTCCGTCAATATTTACCTTCCCTCCCTCAATCGGTTGAATGTCGCCCTTTTGTCTTTAGCGCTGGTAA 2720
ACCATATGAATTTTCTATTGATTGTGACAAAATAAACTTATTCCGTGGTGTCTTTGCGTTTCTTTTATATGTTGCCACCTTTATG 2805
TATGTATTTTCTACGTTTGCTAACATACTGCGTAATAAGGAGTCTTAATCATGCCAGTTCTTTTGGGTATTCCGTTATTATTGCG 2890
TTTCCTCGGTTTCCTTCTGGTAACTTTGTTCGGCTATCTGCTTACTTTTCTTAAAAAGGGCTTCGGTAAGATAGCTATTGCTATT 2975
TCATTGTTTCTTGCTCTTATTATTGGGCTTAACTCAATTCTTGTGGGTTATCTCTCTGATATTAGCGCTCAATTACCCTCTGACT 3060
TTGTTCAGGGTGTTCAGTTAATTCTCCCGTCTAATGCGCTTCCCTGTTTTTATGTTATTCTCTCTGTAAAGGCTGCTATTTTCAT 3145
TTTTGACGTTAAACAAAAAATCGTTTCTTATTTGGATTGGGATAAATAATATGGCTGTTTATTTTGTAACTGGCAAATTAGGCTC 3230
TGGAAAGACGCTCGTTAGCGTTGGTAAGATTCAGGATAAAATTGTAGCTGGGTGCAAAATAGCAACTAATCTTGATTTAAGGCTT 3315
CAAAACCTCCCGCAAGTCGGGAGGTTCGCTAAAACGCCTCGCGTTCTTAGAATACCGGATAAGCCTTCTATATCTGATTTGCTTG 3400
CTATTGGGCGCGGTAATGATTCCTACGATGAAAATAAAAACGGCTTGCTTGTTCTCGATGAGTGCGGTACTTGGTTTAATACCCG 3485
TTCTTGGAATGATAAGGAAAGACAGCCGATTATTGATTGGTTTCTACATGCTCGTAAATTAGGATGGGATATTATTTTTCTTGTT 3570
CAGGACTTATCTATTGTTGATAAACAGGCGCGTTCTGCATTAGCTGAACATGTTGTTTATTGTCGTCGTCTGGACAGAATTACTT 3655
TACCTTTTGTCGGTACTTTATATTCTCTTATTACTGGCTCGAAAATGCCTCTGCCTAAATTACATGTTGGCGTTGTTAAATATGG 3740
CGATTCTCAATTAAGCCCTACTGTTGAGCGTTGGCTTTATACTGGTAAGAATTTGTATAACGCATATGATACTAAACAGGCTTTT 3825
TCTAGTAATTATGATTCCGGTGTTTATTCTTATTTAACGCCTTATTTATCACACGGTCGGTATTTCAAACCATTAAATTTAGGTC 3910
AGAAGATGAAATTAACTAAAATATATTTGAAAAAGTTTTCTCGCGTTCTTTGTCTTGCGATTGGATTTGCATCAGCATTTACATA 3995
TAGTTATATAACCCAACCTAAGCCGGAGGTTAAAAAGGTAGTCTCTCAGACCTATGATTTTGATAAATTCACTATTGACTCTTCT 4080
CAGCGTCTTAATCTAAGCTATCGCTATGTTTTCAAGGATTCTAAGGGAAAATTAATTAATAGCGACGATTTACAGAAGCAAGGTT 4165
ATTCACTCACATATATTGATTTATGTACTGTTTCCATTAAAAAAGGTAATTCAAATGAAATTGTTAAATGTAATTAATTTTGTTT 4250
TCTTGATGTTTGTTTCATCATCTTCTTTTGCTCAGGTAATTGAAATGAATAATTCGCCTCTGCGCGATTTTGTAACTTGGTATTC 4335
AAAGCAATCAGGCGAATCCGTTATTGTTTCTCCCGATGTAAAAGGTACTGTTACTGTATATTCATCTGACGTTAAACCTGAAAAT 4420
CTACGCAATTTCTTTATTTCTGTTTTACGTGCTAATAATTTTGATATGGTTGGTTCAATTCCTTCCATAATTCAGAAGTATAATC 4505
CAAACAATCAGGATTATATTGATGAATTGCCATCATCTGATAATCAGGAATATGATGATAATTCCGCTCCTTCTGGTGGTTTCTT 4590
TGTTCCGCAAAATGATAATGTTACTCAAACTTTTAAAATTAATAACGTTCGGGCAAAGGATTTAATACGAGTTGTCGAATTGTTT 4675
GTAAAGTCTAATACTTCTAAATCCTCAAATGTATTATCTATTGACGGCTCTAATCTATTAGTTGTTAGTGCACCTAAAGATATTT 4760
TAGATAACCTTCCTCAATTCCTTTCTACTGTTGATTTGCCAACTGACCAGATATTGATTGAGGGTTTGATATTTGAGGTTCAGCA 4845
AGGTGATGCTTTAGATTTTTCATTTGCTGCTGGCTCTCAGCGTGGCACTGTTGCAGGCGGTGTTAATACTGACCGCCTCACCTCT 4930
GTTTTATCTTCTGCTGGTGGTTCGTTCGGTATTTTTAATGGCGATGTTTTAGGGCTATCAGTTCGCGCATTAAAGACTAATAGCC 5015
ATTCAAAAATATTGTCTGTGCCACGTATTCTTACGCTTTCAGGTCAGAAGGGTTCTATCTCTGTTGGCCAGAATGTCCCTTTTAT 5100
TACTGGTCGTGTGACTGGTGAATCTGCCAATGTAAATAATCCATTTCAGACGATTGAGCGTCAAAATGTAGGTATTTCCATGAGC 5185
GTTTTTCCTGTTGCAATGGCTGGCGGTAATATTGTTCTGGATATTACCAGCAAGGCCGATAGTTTGAGTTCTTCTACTCAGGCAA 5270
GTGATGTTATTACTAATCAAAGAAGTATTGCTACAACGGTTAATTTGCGTGATGGACAGACTCTTTTACTCGGTGGCCTCACTGA 5355
TTATAAAAACACTTCTCAAGATTCTGGCGTACCGTTCCTGTCTAAAATCCCTTTAATCGGCCTCCTGTTTAGCTCCCGCTCTGAT 5440
TCCAACGAGGAAAGCACGTTATACGTGCTCGTCAAAGCAACCATAGTACGCGCCCTGTAGCGGCGCATTAAGCGCGGCGGGTGTG 5525
GTGGTTACGCGCAGCGTGACCGCTACACTTGCCAGCGCCCTAGCGCCCGCTCCTTTCGCTTTCTTCCCTTCCTTTCTCGCCACGT 5610
TCGCCGGCTTTCCCCGTCAAGCTCTAAATCGGGGGCTCCCTTTAGGGTTCCGATTTAGTGCTTTACGGCACCTCGACCCCAAAAA 5695
ACTTGATTTGGGTGATGGTTCACGTAGTGGGCCATCGCCCTGATAGACGGTTTTTCGCCCTTTGACGTTGGAGTCCACGTTCTTT 5780
AATAGTGGACTCTTGTTCCAAACTGGAACAACACTCAACCCTATCTCGGGCTATTCTTTTGATTTATAAGGGATTTTGCCGATTT 5865
CGGCCCGTcTCTGGATAATGTTTTTTGCGCCGACATCATAACGGTTCTGGCAAATATTCTGAAATGAGCTGTTGACAATTAATCA 5950
TCGGCTCGTATAATGTGTGGAATTGTGAGCGGATAACAATTTCACACAGGAAACAgagcacatcagcAGGaGATCTAAAGAGGAG 6035
AAAGGATCTatgaactacaccgacattcaggcacgtctggcaatcatcaagtcactcccgattagcgaactggacaagcgccagc 6120
ctcttctggttgcactggctgctgacatcgtgaacggtgagacaagcgatggcaacgatactgatggctcaaatggcttggagta 6205
tcaagactggtggcacacattgggtgcactgatgcgtgatgcaggtttccgcatgttgggtaatggtcactttagtgcggcctac 6290
tcgcatgaactgttaccgggtcgagtcatcaaggttggctttaagaaagaggattcaggcgctgcgtacactgcgttctgtagga 6375
tgcaccaaggccgagcgggtatccctaacgtctatcacgtagcacgacatgcaggttgctatacggtcgtactggaccgcttaca 6460
aagctgtgaccgctatgctaacgatgtgcacgcaaagtatgcaagtgctgcgcaggagttcattgagtgcactggaggtgatgct 6545
gattggtacgcacagtatgttagcaatgagttcattgagacgtgcaagatgattcgtgagttcttccacggtattgcatctttcg 6630
acatgcacagcgggaacattatgttcgatgataacgatgtcccgtacatcactgacccggttagtttctcacatgaccgtgaacg 6715
tgaggacggtttcctctggaccctgaagcactgctggctgaggtcgaggctgtagcgcaagaacgcatgattgaacggtgccgc 6800
aaccgcaaggccaagtgtgaccctaacggaacctttcaggttaaccgcaaggccgcaatgaaacgccgtaagcgcaaccgcaagt 6885
tacgcgccaaggtagctgaacgtgaccgcttacacttcatggcaatccgtagggaacgcggtgtgattgagcgtaacgaacgccg 6970
```

```
tgctgagatgctcatggggtctgcatggcatgacttctggttacgcaatggtaacgcaacggttcgcaagattgaccaagtgaac        7055
ggccttaagtggcaactaggggaccgcctcgcaatacaagcgggtctccctttgaacatcgacaaggtacttgacgctcacctga        7140
tgggcTgAACAAAAATTTAACGCGAATTTTAACAAAATATTAACGTTTACAATTTAAATATTTGCTTATACAATCTTCCTGTTTT        7225
TGGGGCTTTTCTGATTATCAACCGGGGTACATATGATTGACATGCTAGTTTTACGATTACCGTTCATCGATTCTCTTGTTTGCTC        7310
CAGACTCTCAGGCAATGACCTGATAGCCTTTGTAGACCTCTCAAAAATAGCTACCCTCTCCGGCATGAATTTATCAGCTAGAACG        7395
GTTGAATATCATATTGATGGTGATTTGACTGTCTCCGGCCTTTCTCACCCTTTTGAATCTTTACCTACACATTACTCAGGCATTG        7480
CATTTAAAATATATGAGGGTTCTAAAAATTTTTATCCTTGCGTTGAAATAAAGGCTTCTCCCGCAAAAGTATTACAGGGTCATAA        7565
TGTTTTTGGTACAACCGATTTAGCTTTATGCTCTGAGGCTTTATTGCTTAATTTTGCTAATTCTTTGCCTTGCCTGTATGATTTA        7650
TTGGATGTT        7659
```

M13-JSS1_004-deletions-F.ab1 (1214 bases)

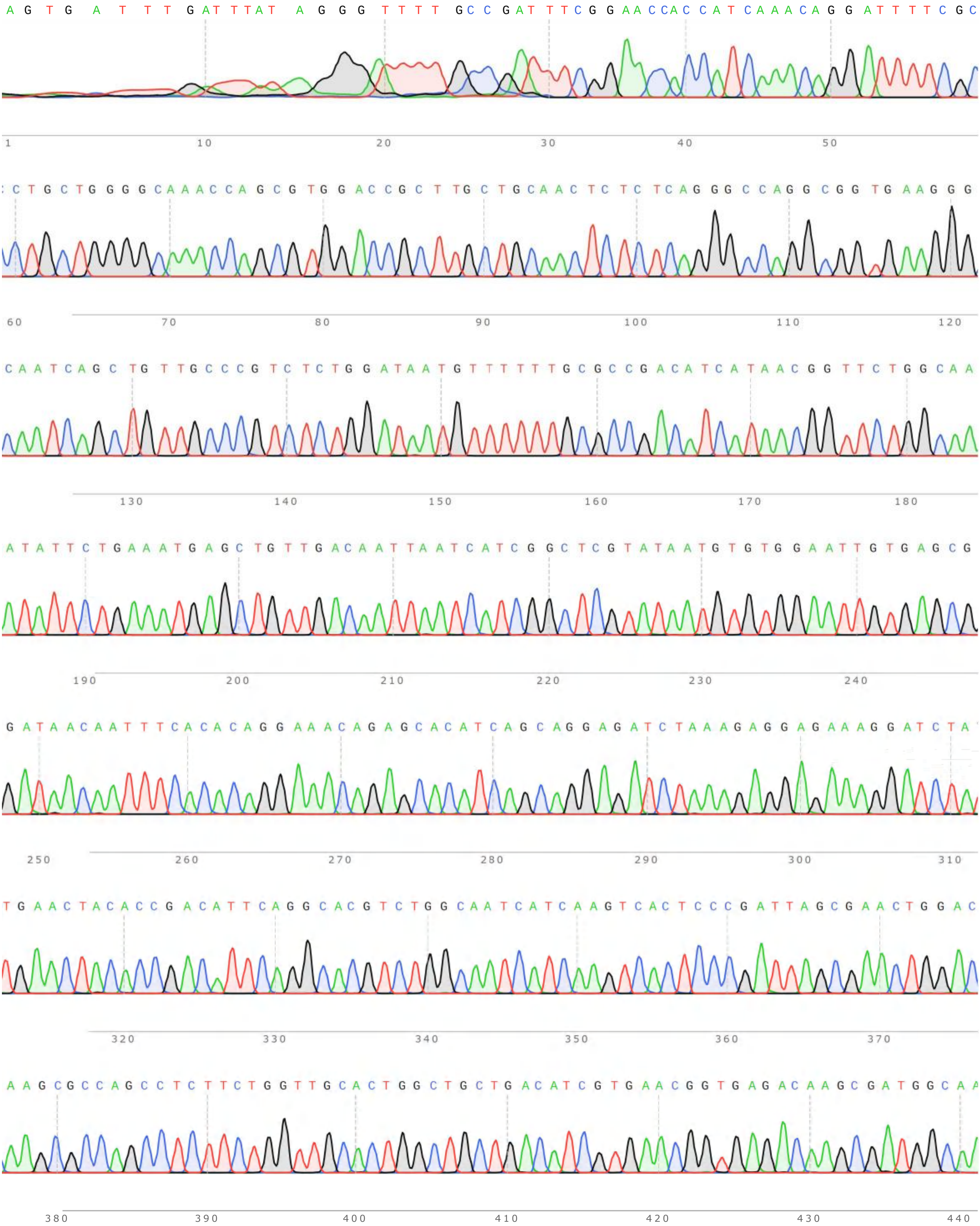

A C G A T A C T G A T G G C T C A A A T G G C T T G G A G T A T C A A G A C T G G T G G C A C A C A T T G G G T G C A C T G A T G C

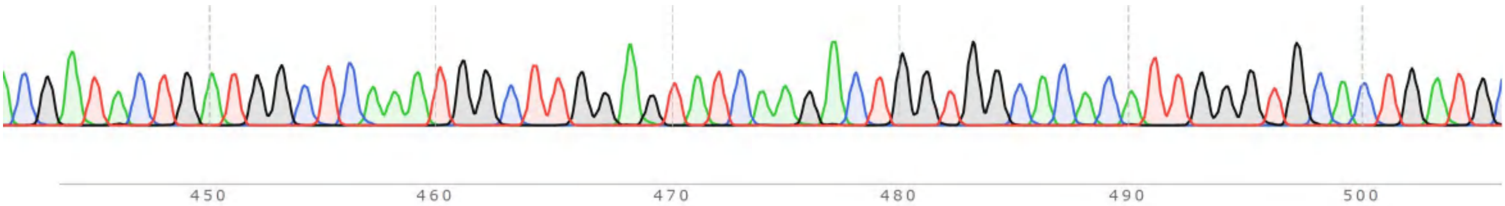

C G T G A T G C A G G T T T C C G C A T G G G T A A T G G T C A C T T T A G T G C G G C C T A C T C G C A T G A A C T G T T A C C

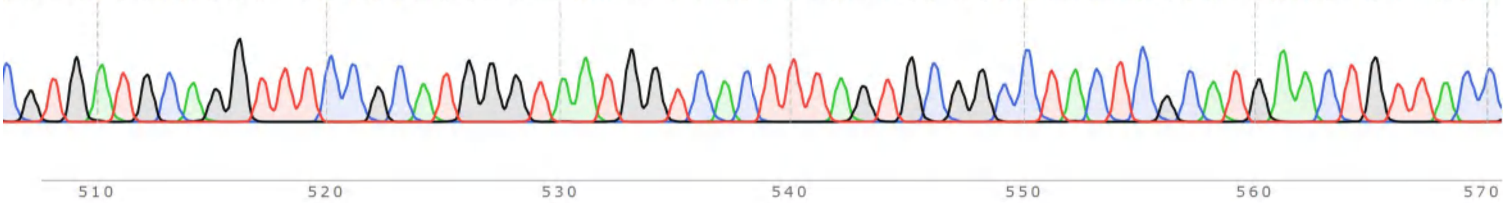

G G G T C G A G T C A T C A A G G T T G G C T T T A A G A A A G A G G A T T C A G G C G C T G C G T A C A C T G C G T T C T G T

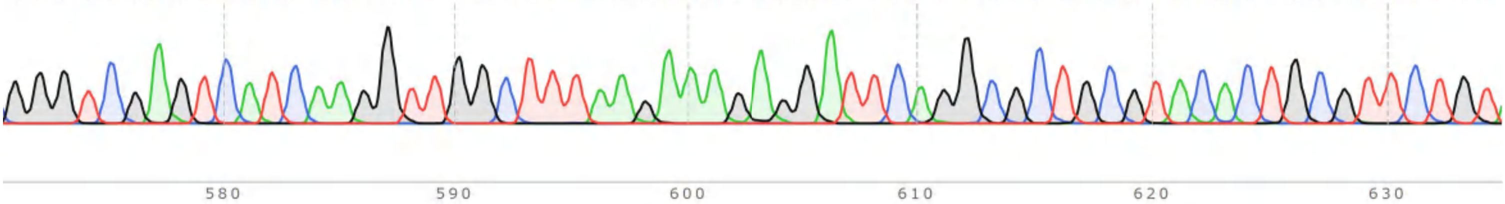

A G G A T G C A C C A A G G C C G A G C G G G T A T C C C T A A C G T C T A T C A C G T A G C A C G A C A T G C A G G T T G C T A

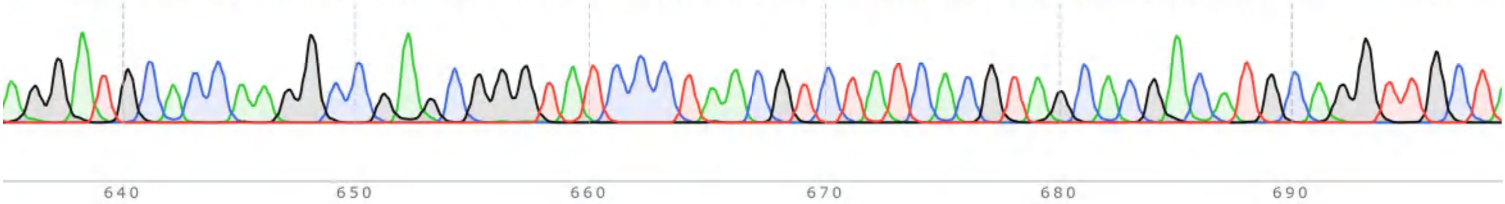

A T A C G G T C G T A C T G G A C C G C T T A C A A A G C T G T G A C C G C T A T G C T A A C G A T G T G C A C G C A A A G T A T

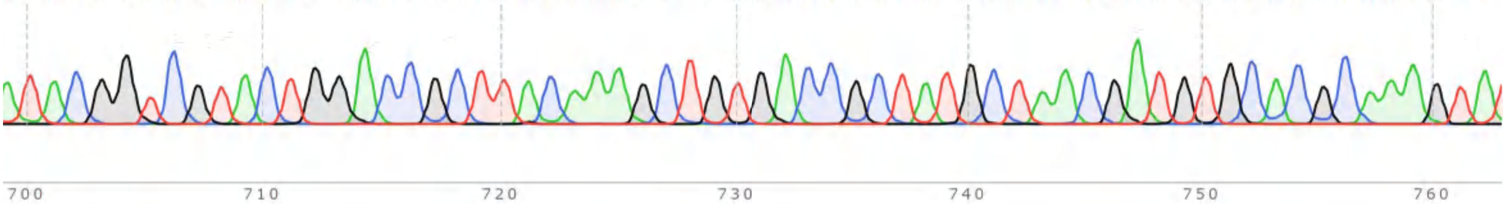

T G C A A G T G C T G C G C A G G A G T T C A T T G A G T G C A C T G G A G G T G A T G C T G A T T G G T A C G C A C A G T A T

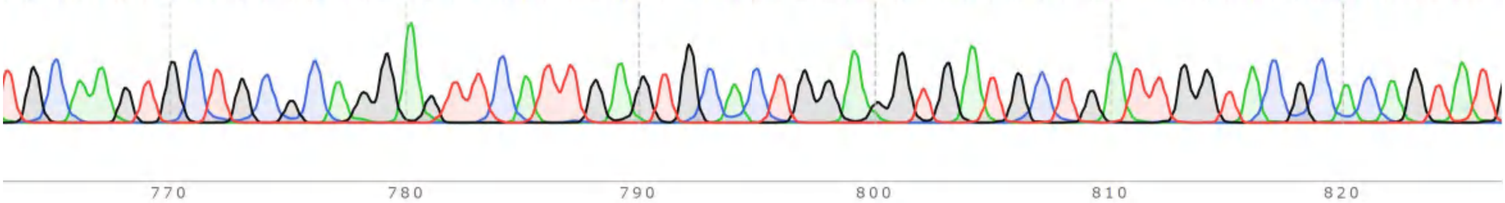

G T T A G C A A T G A G T T C A T T G A G A C G T G C A A G A T G A T T C G T G A G T T C T T C C A C G G T A T T G C A T C T T

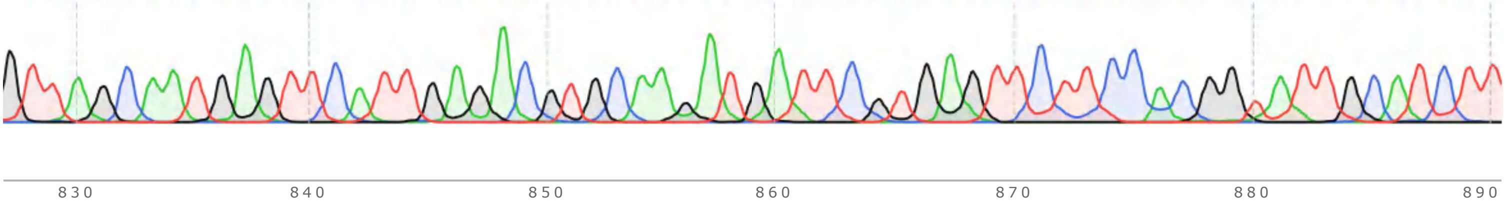

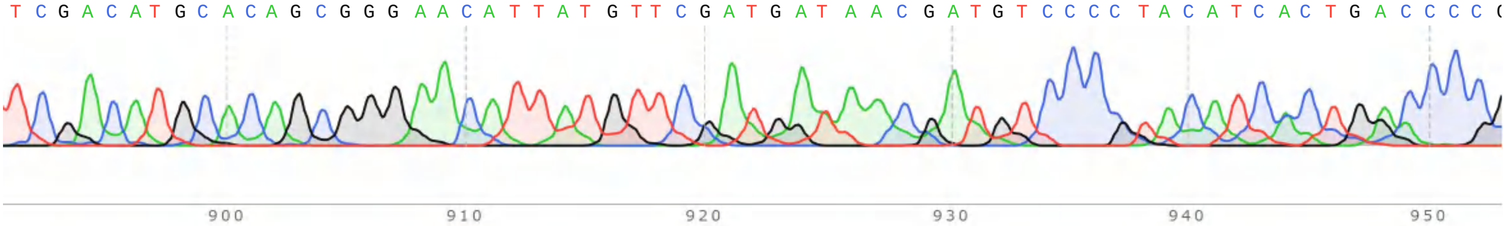
T C G A C A T G C A C A G C G G G A A C A T T A T G T T C G A T G A T A A C G A T G T C C C C T A C A T C A C T G A C C C C
900 910 920 930 940 950

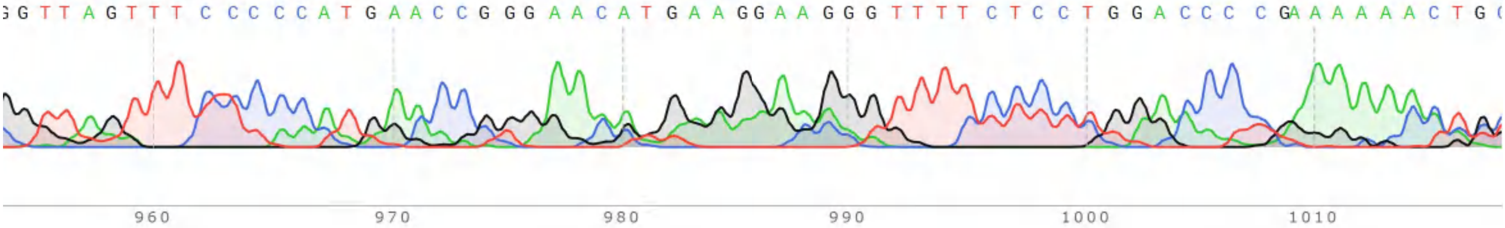
G T T A G T T T C C C C C A T G A A C C G G G A A C A T G A A G G A A G G G T T T T C T C C T G G A C C C C G A A A A A A C T G
960 970 980 990 1000 1010

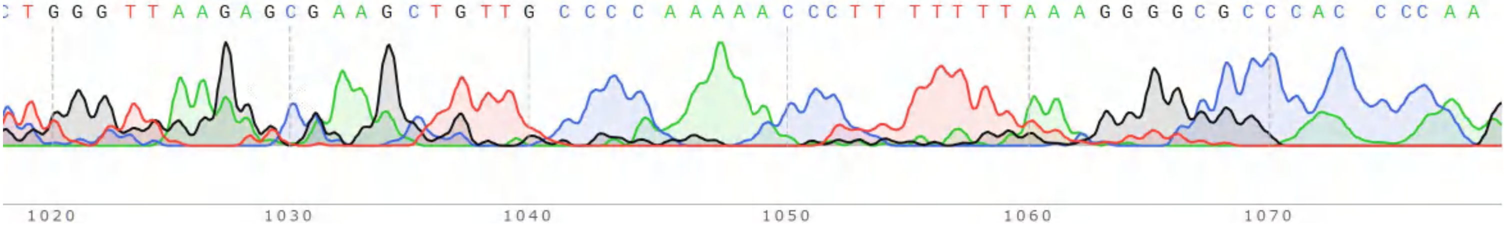
T G G G T T A A G A G C G A A G C T G T T G C C C C A A A A A C C C T T T T T T T A A A G G G G C G C C C A C C C C A A
1020 1030 1040 1050 1060 1070

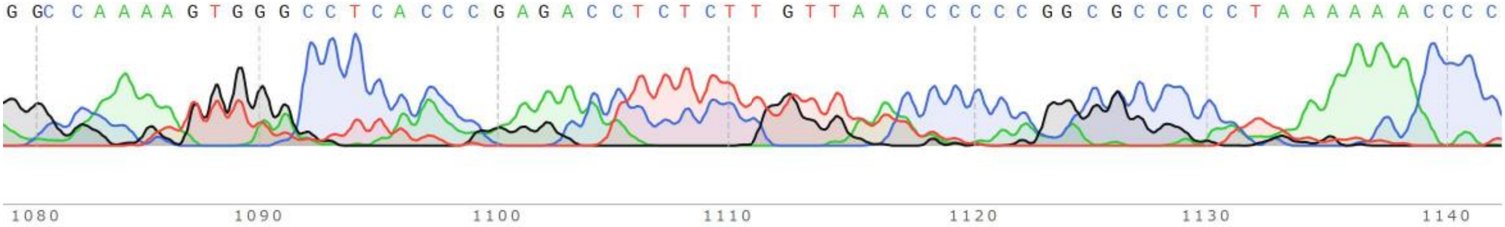
G G C C A A A A G T G G G C C T C A C C C G A G A C C T C T C T T G T T A A C C C C C C G G C G C C C C C T A A A A A A C C C C
1080 1090 1100 1110 1120 1130 1140

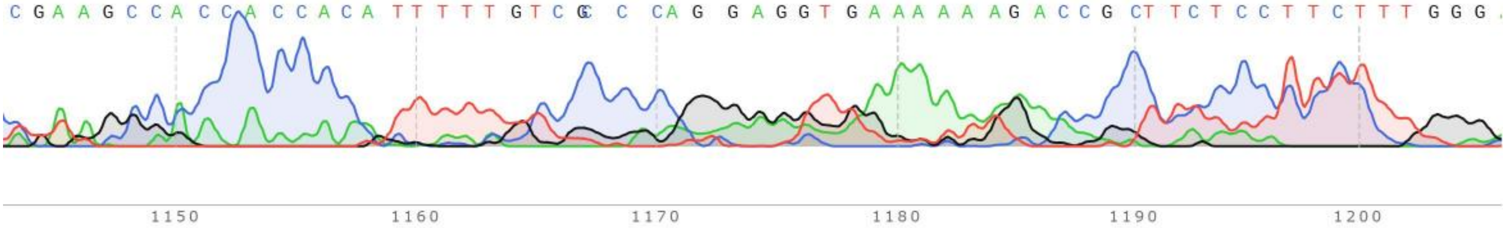
C G A A G C C A C C A C C A C A T T T T T G T C G C C A G G A G G T G A A A A A A G A C C G C T T C T C C T T C T T T G G G
1150 1160 1170 1180 1190 1200

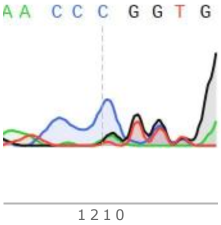
A A C C C G G T G
1210

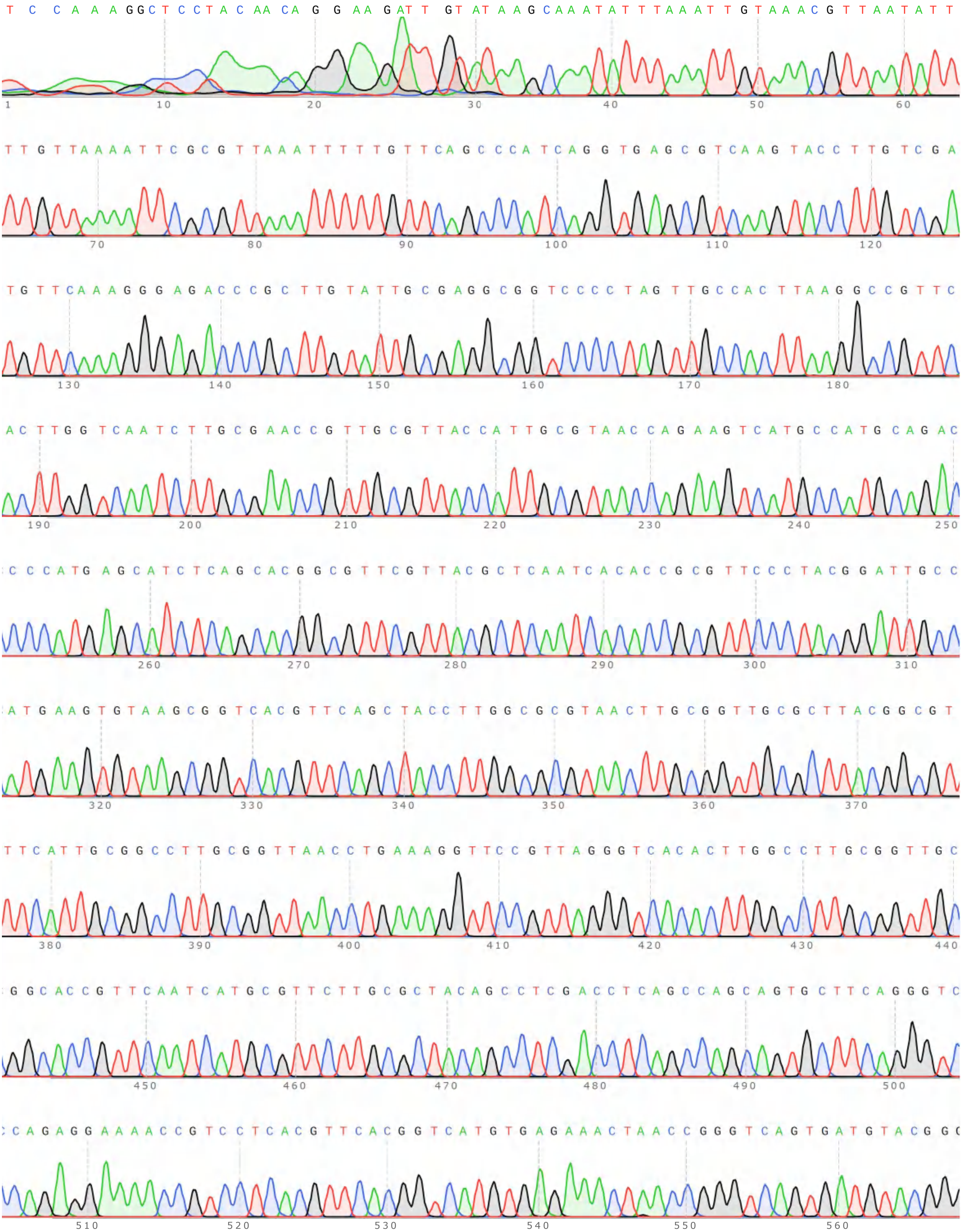
TCCAAAGGCTCCTACAACAGGAAGATTGTATAAGCAAATATTTAAATTGTAAACGTTAATATT
1 10 20 30 40 50 60
TTGTTAAAATTCGCGTTAAATTTTTGTTCAGCCCATCAGGTGAGCGTCAAGTACCTTGTCGA
70 80 90 100 110 120
TGTTCAAAGGGAGACCCGCTTGTATTGCGAGGCGGTCCCCTAGTTGCCACTTAAGGCCGTTC
130 140 150 160 170 180
ACTTGGTCAATCTTGCGAACCGTTGCGTTACCATTGCGTAACCAGAAGTCATGCCATGCAGAC
190 200 210 220 230 240 250
CCCATGAGCATCTCAGCACGGCGTTCGTTACGCTCAATCACACCGCGTTCCCTACGGATTGCC
260 270 280 290 300 310
ATGAAGTGTAAGCGGTCACGTTCAGCTACCTTGGCGCGTAACTTGCGGTTGCGCTTACGGCGT
320 330 340 350 360 370
TTCATTGCGGCCTTGCGGTTAACCTGAAAGGTTCCGTTAGGGTCACACTTGGCCTTGCGGTTGC
380 390 400 410 420 430 440
GGCACCGTTCAATCATGCGTTCTTGCGCTACAGCCTCGACCTCAGCCAGCAGTGCTTCAGGGTC
450 460 470 480 490 500
CAGAGGAAAACCGTCCTCACGTTCACGGTCATGTGAGAAACTAACCGGGTCAGTGATGTACGGC
510 520 530 540 550 560

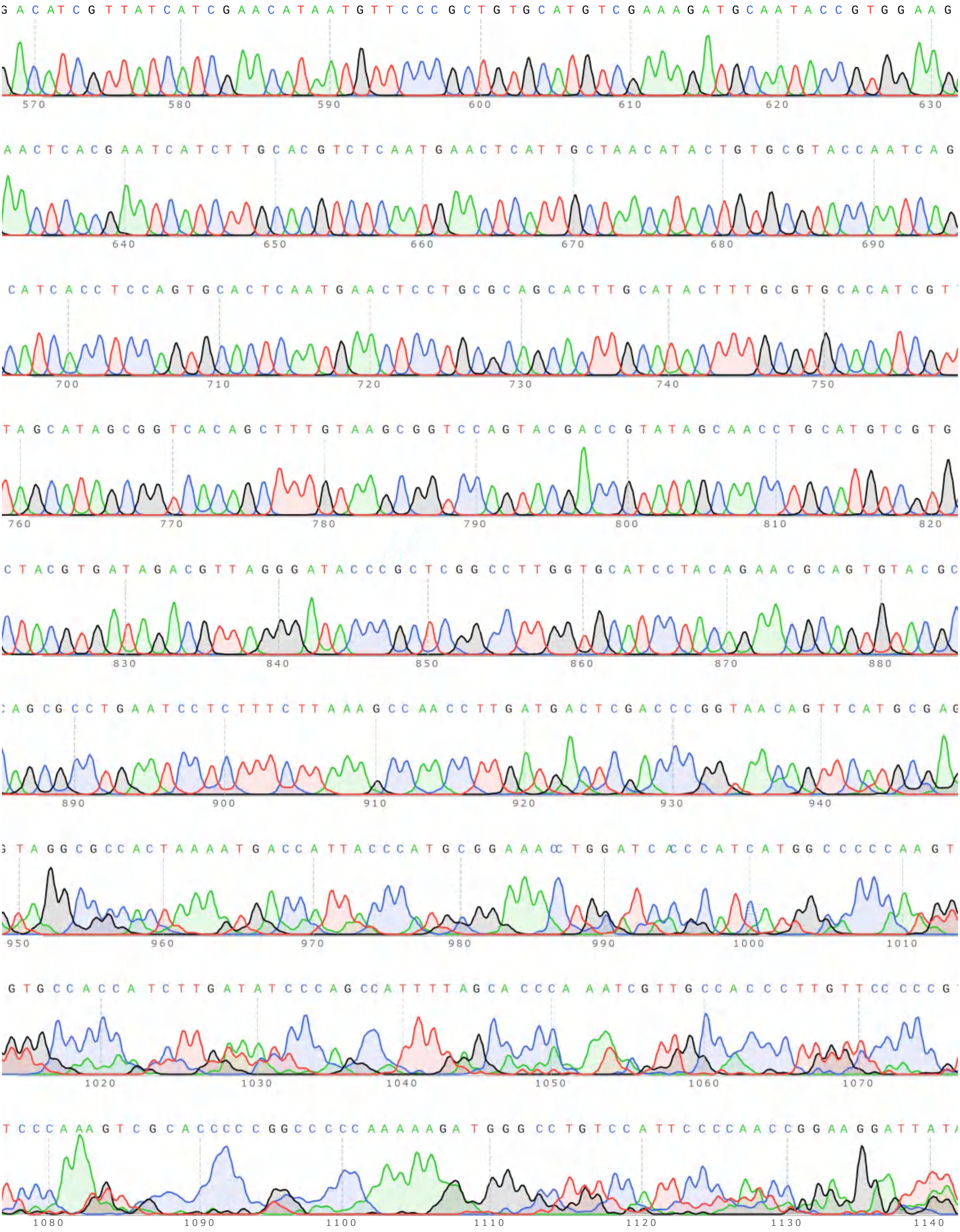
GACATCGTTATCATCGAACATAATGTTCCCGCTGTGCATGTCGAAAGATGCAATACCGTGGAAG
AACTCACGAATCATCTTGCACGTCTCAATGAACTCATTGCTAACATACTGTGCGTACCAATCAG
CATCACCTCCAGTGCACTCAATGAACTCCTGCGCAGCACTTGCATACTTTGCGTGCACATCGT
TAGCATAGCGGTCACAGCTTTGTAAGCGGTCCAGTACGACCGTATAGCAACCTGCATGTCGTG
CTACGTGATAGACGTTAGGGATACCCGCTCGGCCTTGGTGCATCCTACAGAACGCAGTGTACGC
AGCGCCTGAATCCTCTTTCTTAAAGCCAACCTTGATGACTCGACCCGGTAACAGTTCATGCGAG
TAGGCGCCACTAAAATGACCATTACCCATGCGGAAACCTGGATCACCCATCATGGCCCCCAAGT
GTGCCACCATCTTGATATCCCAGCCATTTTAGCACCCAAATCGTTGCCACCCTTGTTCCCCCG
TCCCAAAGTCGCACCCCCGGCCCCCAAAAAGATGGGCCTGTCCATTCCCCAACCGGAAGGATTATA

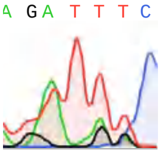
A G A T T T C

AT T T TC G C C G AT T T C G G A A C CAC C A T C A A A C A G G A T T T T C G C C T G C T G G G G C A A A C C A

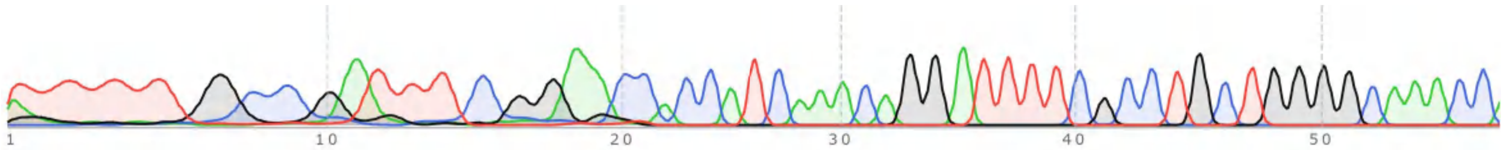

A G C G T G G A C C G C T T G C T G C A A C T C T C T C A G G G C C A G G C G G T G A A G G G C A A T C A G C T G T T G C C

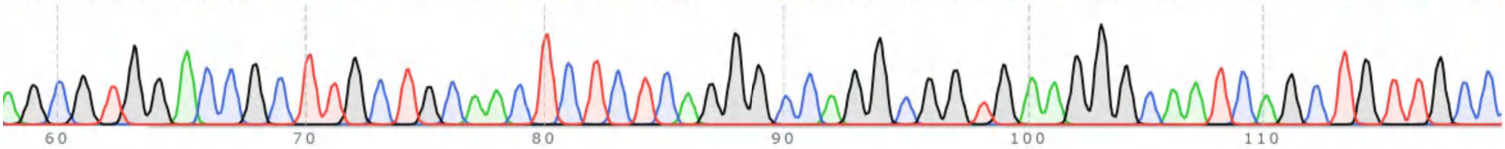

C G T C T C T G G A T A A T G T T T T T T G C G C C G A C A T C A T A A C G G T T C T G G C A A A T A T T C T G A A A T G A G

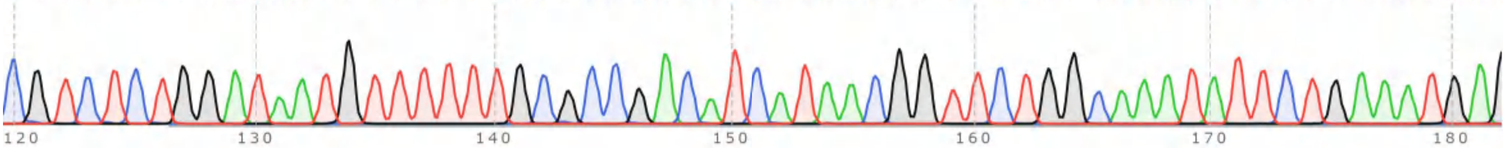

G C T G T T G A C A A T T A A T C A T C G G C T C G T A T A A T G T G T G G A A T T G T G A G C G G A T A A C A A T T T C A C

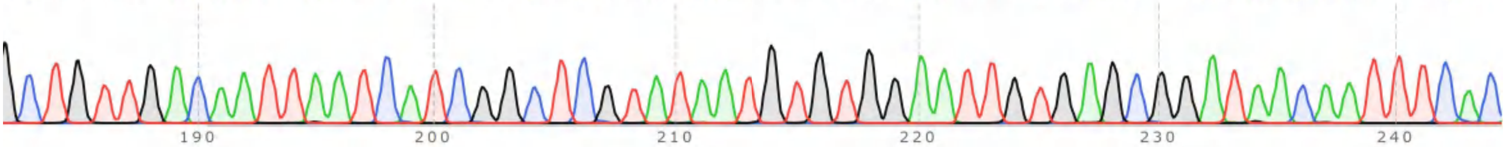

A C A G G A A A C A G A G C A C A T C A G C A G G A G A T C T A A A G A G G A G A A A G G A T C T A T G A A C T A C A C C G A

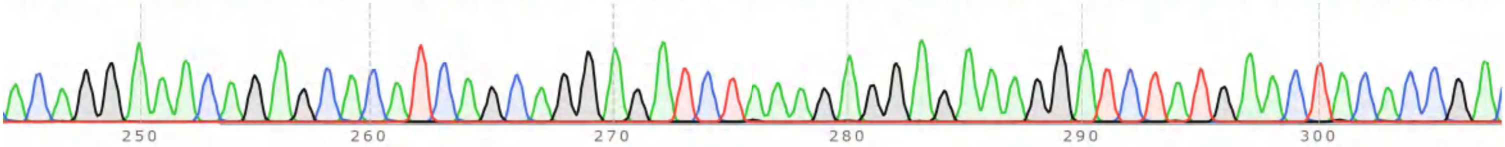

C A T T C A G G C A C G T C T G G C A A T C A T C A A G T C A C T C C C G A T T A G C G A A C T G G A C A A G C G C C A G C C T

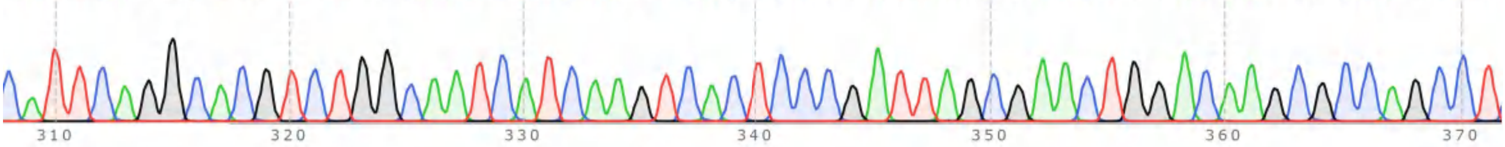

C T T C T G G T T G C A C T G G C T G C T G A C A T C G T G A A C G G T G A G A C A A G C G A T G G C A A C G A T A C T G A T G

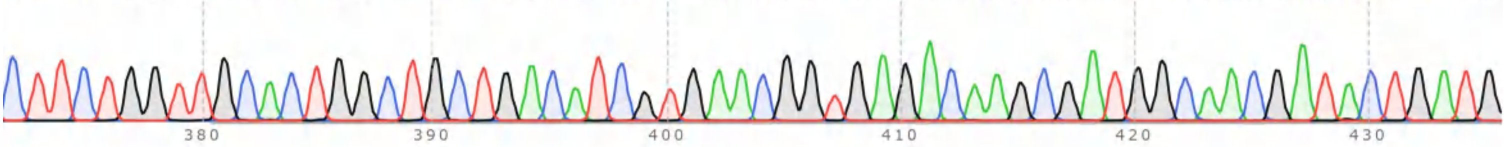

G C T C A A A T G G C T T G G A G T A T C A A G A C T G G T G G C A C A C A T T G G G T G C A C T G A T G C G T G A T G C A G G

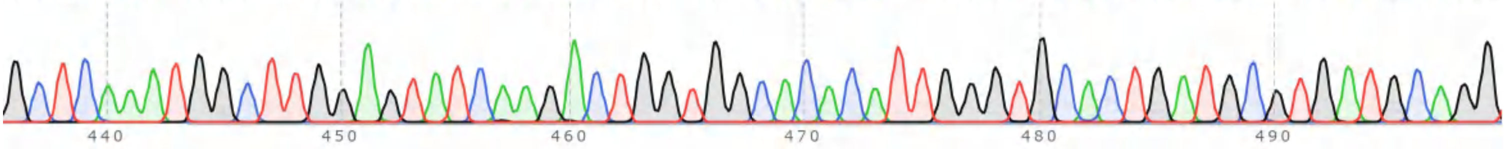

T T T C C G C A T G T T G G G T A A T G G T C A C T T T A G T G C G G C C T A C T C G C A T G A A C T G T T A C C G G G T C G A

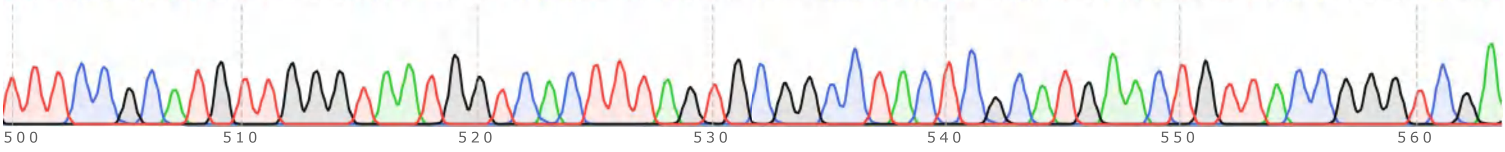

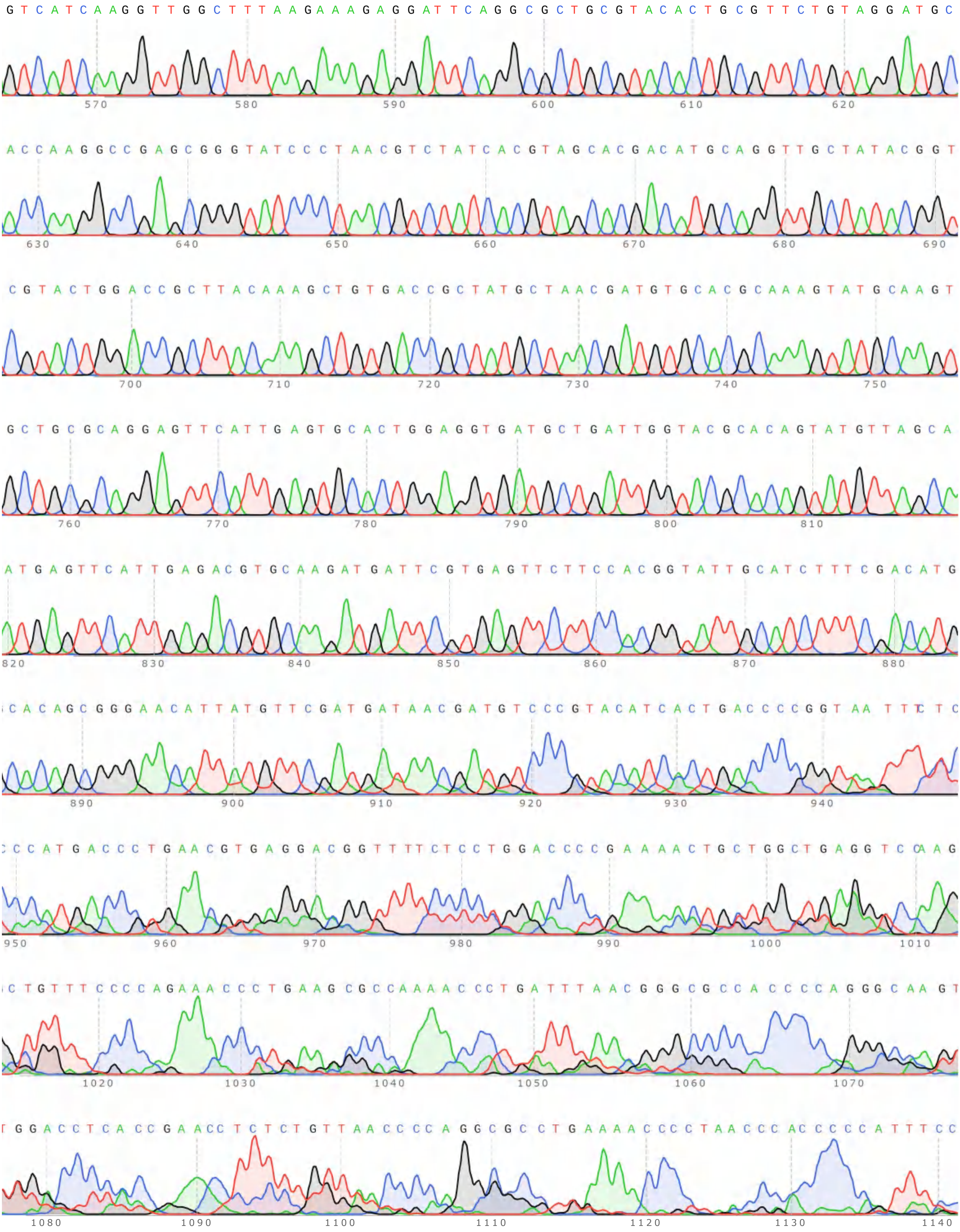
G T C A T C A A G G T T G G C T T T A A G A A A G A G G A T T C A G G C G C T G C G T A C A C T G C G T T C T G T A G G A T G C
A C C A A G G C C G A G C G G G T A T C C C T A A C G T C T A T C A C G T A G C A C G A C A T G C A G G T T G C T A T A C G G T
C G T A C T G G A C C G C T T A C A A A G C T G T G A C C G C T A T G C T A A C G A T G T G C A C G C A A A G T A T G C A A G T
G C T G C G C A G G A G T T C A T T G A G T G C A C T G G A G G T G A T G C T G A T T G G T A C G C A C A G T A T G T T A G C A
A T G A G T T C A T T G A G A C G T G C A A G A T G A T T C G T G A G T T C T T C C A C G G T A T T G C A T C T T T C G A C A T G
C A C A G C G G G A A C A T T A T G T T C G A T G A T A A C G A T G T C C C G T A C A T C A C T G A C C C C G G T A A T T C T C
C C A T G A C C C T G A A C G T G A G G A C G G T T T T C T C C T G G A C C C C G A A A A C T G C T G G C T G A G G T C C A A G
C T G T T T C C C C A G A A A C C C T G A A G C G C C A A A A C C C T G A T T T A A C G G G C G C C A C C C C A G G G C A A G T
T G G A C C T C A C C G A A C C T C T C T G T T A A C C C C A G G C G C C T G A A A A C C C C T A A C C C A C C C C C A T T T C C

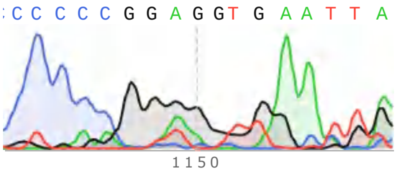
C C C C C C G G A G G T G A A T T A
1150

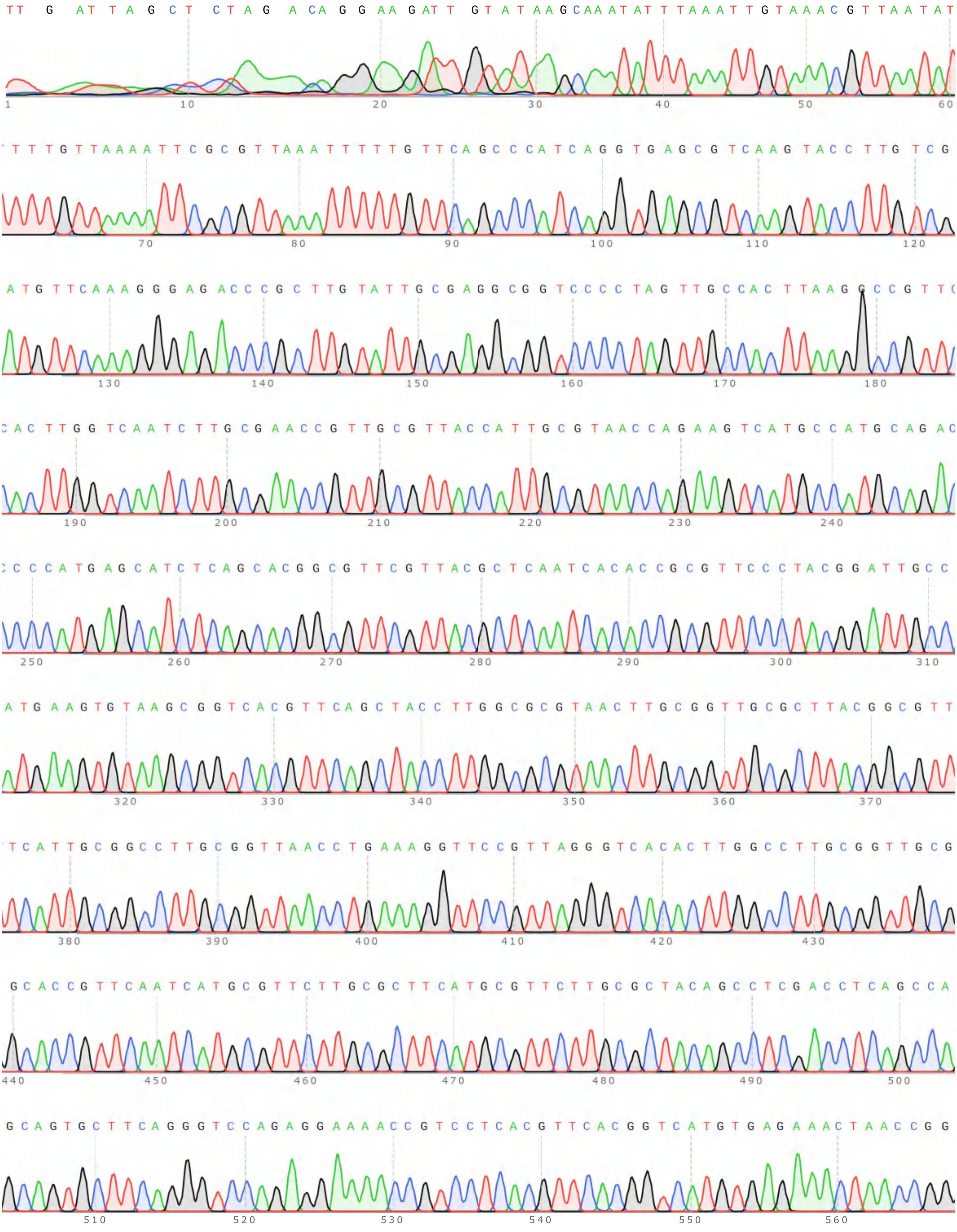
TT G AT T A G C T C T A G A CA G G AA G AT T G T A T A A G C A A A T A T T T A A A T T G T A A A C G T T A A T A T
1 10 20 30 40 50 60
T T T T G T T A A A A T T C G C G T T A A A T T T T T T G T T C A G C C C A T C A G G T G A G C G T C A A G T A C C T T G T C G
70 80 90 100 110 120
A T G T T C A A A G G G A G A C C C G C T T G T A T T G C G A G G C G G T C C C C T A G T T G C C A C T T A A G G C C G T T C
130 140 150 160 170 180
C A C T T G G T C A A T C T T G C G A A C C G T T G C G T T A C C A T T G C G T A A C C A G A A G T C A T G C C A T G C A G A C
190 200 210 220 230 240
C C C C A T G A G C A T C T C A G C A C G G C G T T C G T T A C G C T C A A T C A C A C C G C G T T C C C T A C G G A T T G C C
250 260 270 280 290 300 310
A T G A A G T G T A A G C G G T C A C G T T C A G C T A C C T T G G C G C G T A A C T T G C G G T T G C G C T T A C G G C G T T
320 330 340 350 360 370
T C A T T G C G G C C T T G C G G T T A A C C T G A A A G G T T C C G T T A G G G T C A C A C T T G G C C T T G C G G T T G C G
380 390 400 410 420 430
G C A C C G T T C A A T C A T G C G T T C T T G C G C T T C A T G C G T T C T T G C G C T A C A G C C T C G A C C T C A G C C A
440 450 460 470 480 490 500
G C A G T G C T T C A G G G T C C A G A G G A A A A C C G T C C T C A C G T T C A C G G T C A T G T G A G A A A C T A A C C G G
510 520 530 540 550 560

G T C A G T G A T G T A C G G G A C A T C G T T A T C A T C G A A C A T A A T G T T C C C G C T G T G C A T G T C G A A A G A T

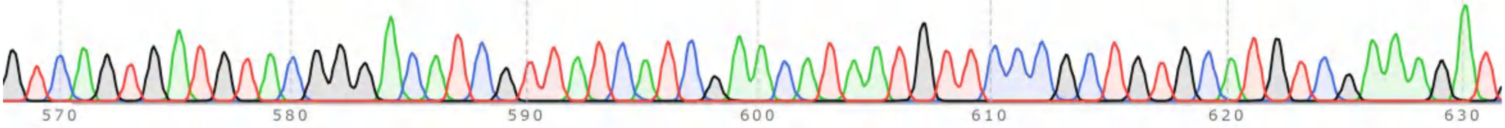

G C A A T A C C G T G G A A G A A C T C A C G A A T C A T C T T G C A C G T C T C A A T G A A C T C A T T G C T A A C A T A C T

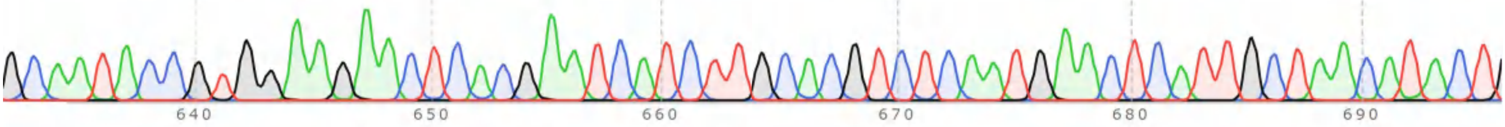

G T G C G T A C C A A T C A G C A T C A C C T C C A G T G C A C T C A A T G A A C T C C T G C G C A G C A C T T G C A T A C T T

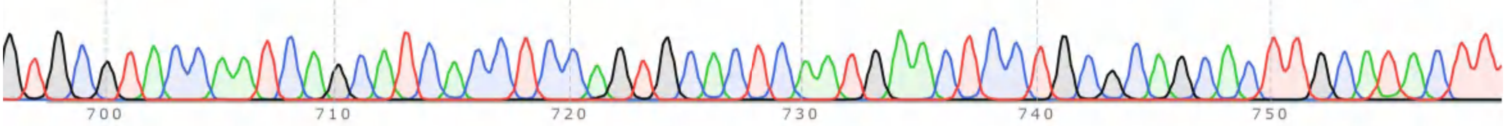

T G C G T G C A C A T C G T T A G C A T A G C G G T C A C A G C T T T G T A A G C G G T C C A G T A C G A C C G T A T A G C A A

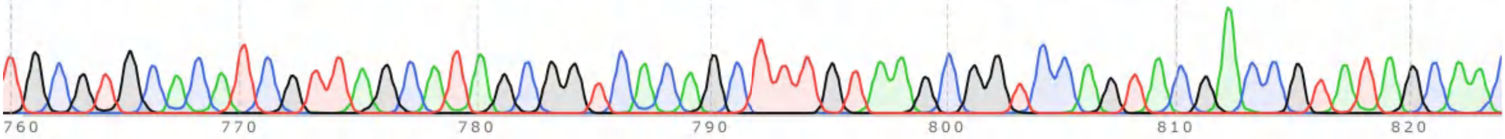

C C T G C A T G T C G T G C T A C G T G A T A G A C G T T A G G G A T A C C C G C T C G G C C T T G G T G C A T C C T A C A G A

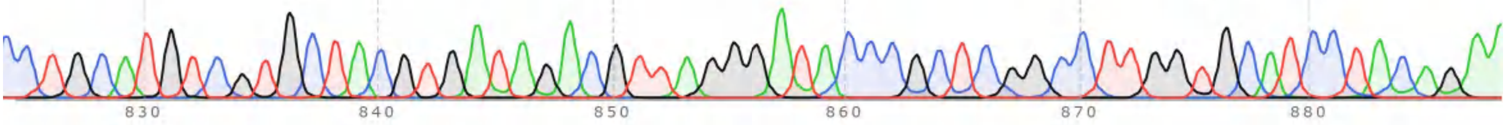

A C G C A G T G T A C G C A G C G C C T G A A T C C T C T T T C T T A A A G C C A A C C T T G A T G A C T C G A C C C G G T A A C

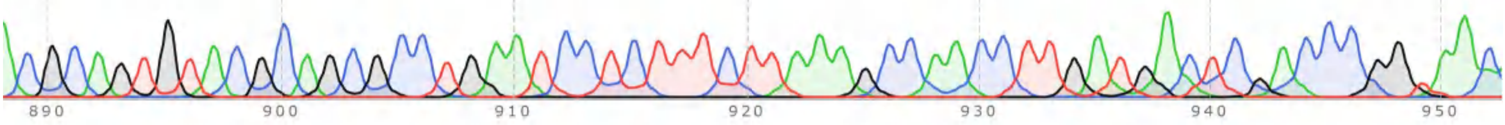

A G T T C A T G C G A G T A G G C G C C A C T A A A G T G A C C T T A C C C C A C A T G C G G A A A C C T G C T C C C C C A T C

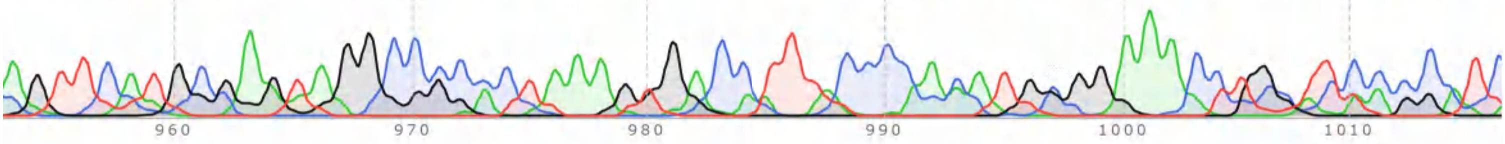

A T T G C C C C C A A G T G T G C C A C A C T C T T T A T A C T C C A G C C T T T T G G C C C C C C A A T T T T T G C C C C

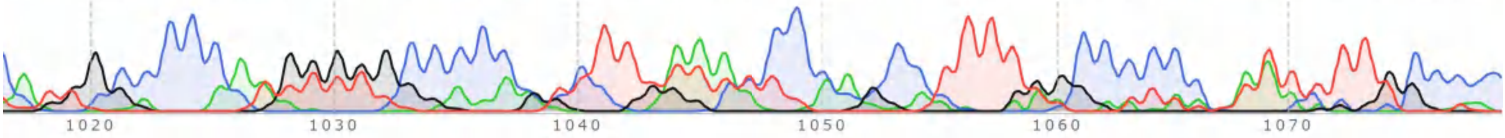

C C T T G T T C C C C C T T C C A A A T T T G C C A C C C A G G A A C C A A A A A A G A T G G G C C T G T C C A T T C T C C A C

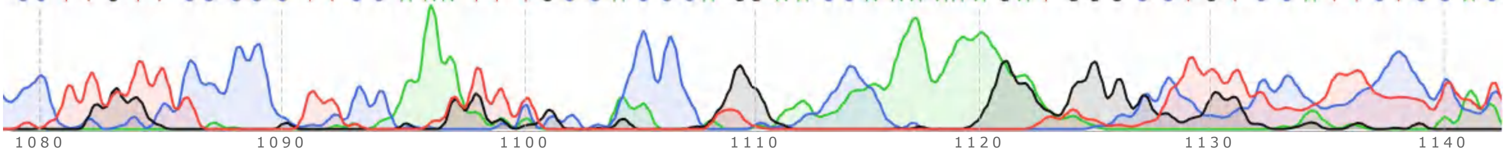

C C G G A G G A G T G T G A T G T C C A A C C C C C C A A G C C C G G G A T

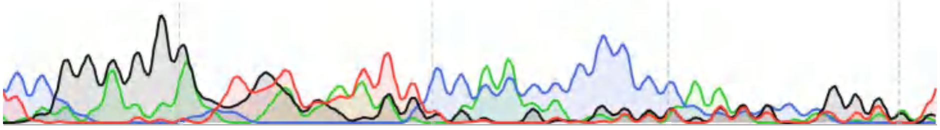

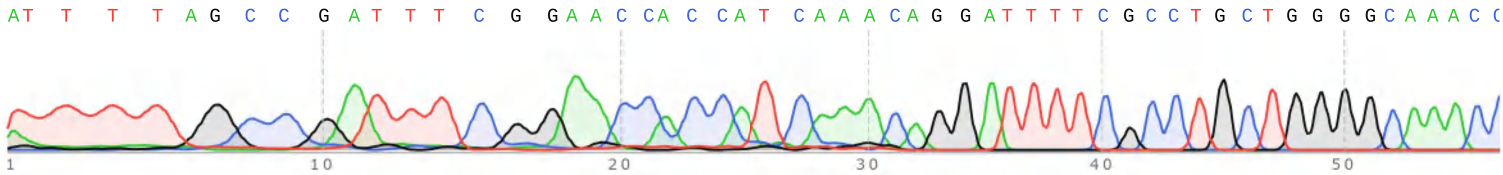
AT T T T A G C C G A T T T C G G A A C CA C CA T C A A A CA G G A T T T T C G C C T G C T G G G G C A A A C C
1 10 20 30 40 50

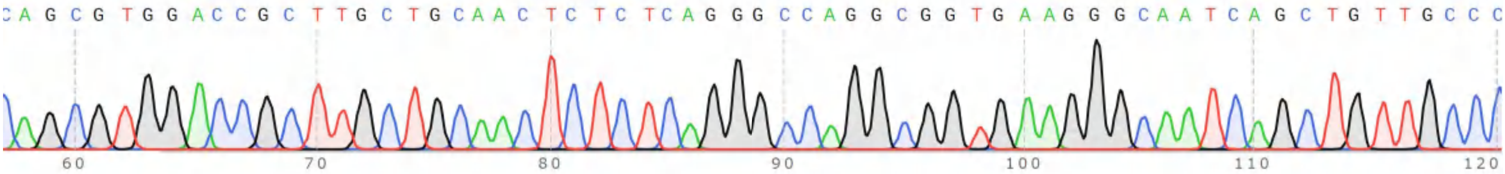
C A G C G T G G A C C G C T T G C T G C A A C T C T C T C A G G G C C A G G C G G T G A A G G G C A A T C A G C T G T T G C C C
60 70 80 90 100 110 120

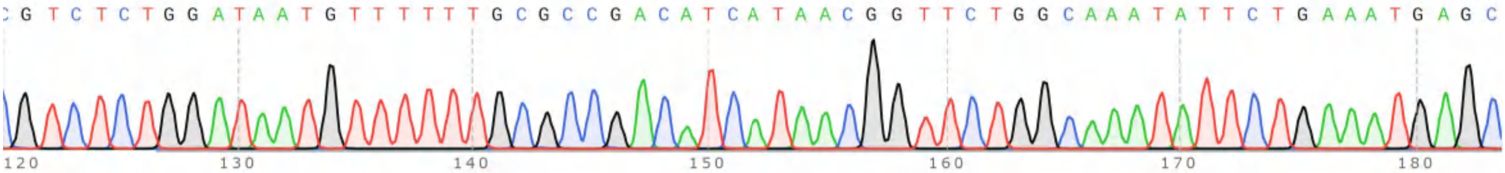
C G T C T C T G G A T A A T G T T T T T T G C G C C G A C A T C A T A A C G G T T C T G G C A A A T A T T C T G A A A T G A G C
120 130 140 150 160 170 180

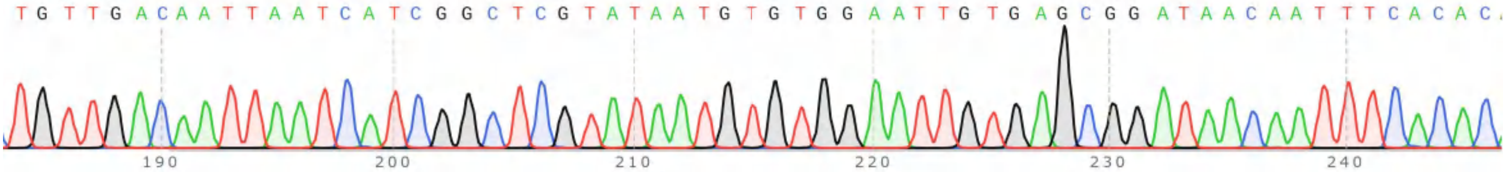
T G T T G A C A A T T A A T C A T C G G C T C G T A T A A T G T G T G G A A T T G T G A G C G G A T A A C A A T T T C A C A C A
190 200 210 220 230 240

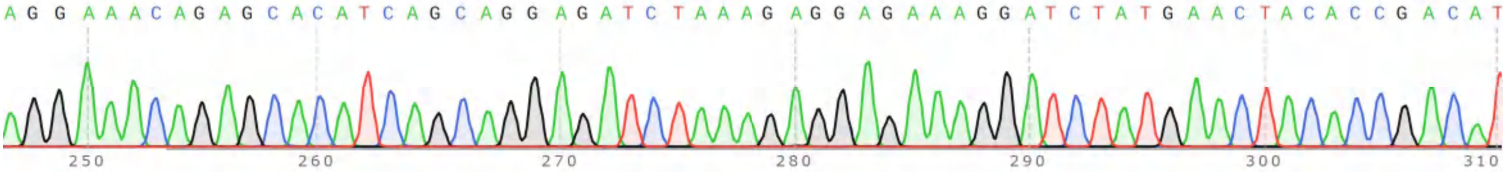
A G G A A A C A G A G C A C A T C A G C A G G A G A T C T A A A G A G G A G A A A G G A T C T A T G A A C T A C A C C G A C A T
250 260 270 280 290 300 310

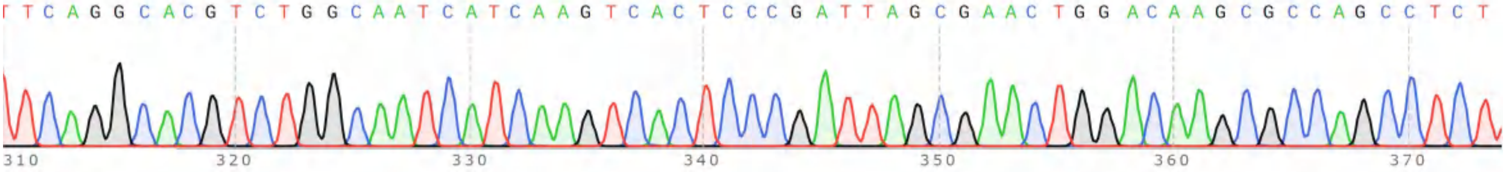
T T C A G G C A C G T C T G G C A A T C A T C A A G T C A C T C C C G A T T A G C G A A C T G G A C A A G C G C C A G C C T C T
310 320 330 340 350 360 370

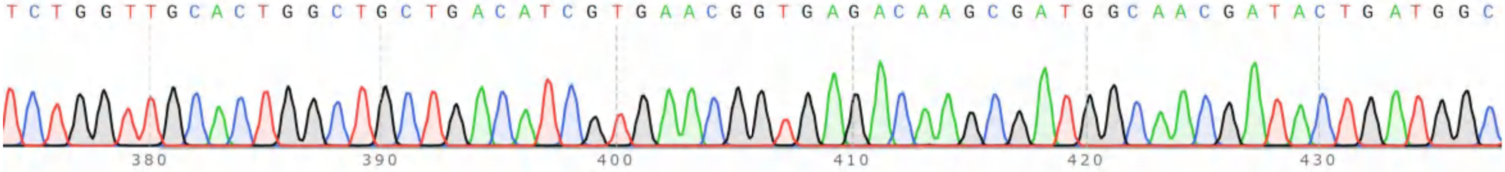
T C T G G T T G C A C T G G C T G C T G A C A T C G T G A A C G G T G A G A C A A G C G A T G G C A A C G A T A C T G A T G G C
380 390 400 410 420 430

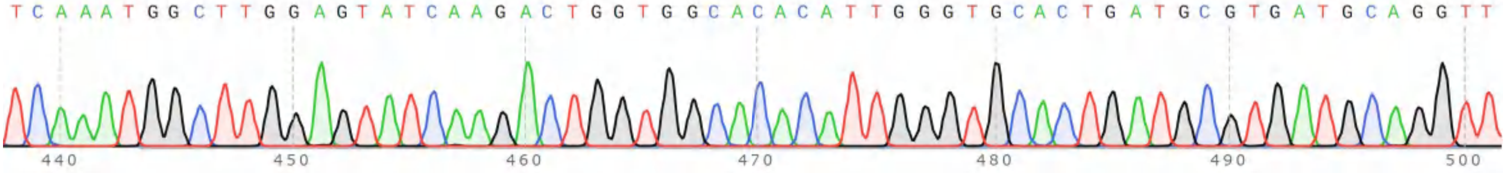
T C A A A T G G C T T G G A G T A T C A A G A C T G G T G G C A C A C A T T G G G T G C A C T G A T G C G T G A T G C A G G T T
440 450 460 470 480 490 500

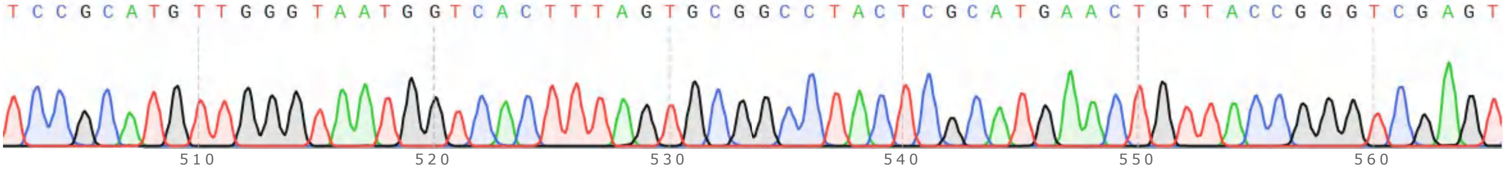
T C C G C A T G T T G G G T A A T G G T C A C T T T A G T G C G G C C T A C T C G C A T G A A C T G T T A C C G G G T C G A G T
510 520 530 540 550 560

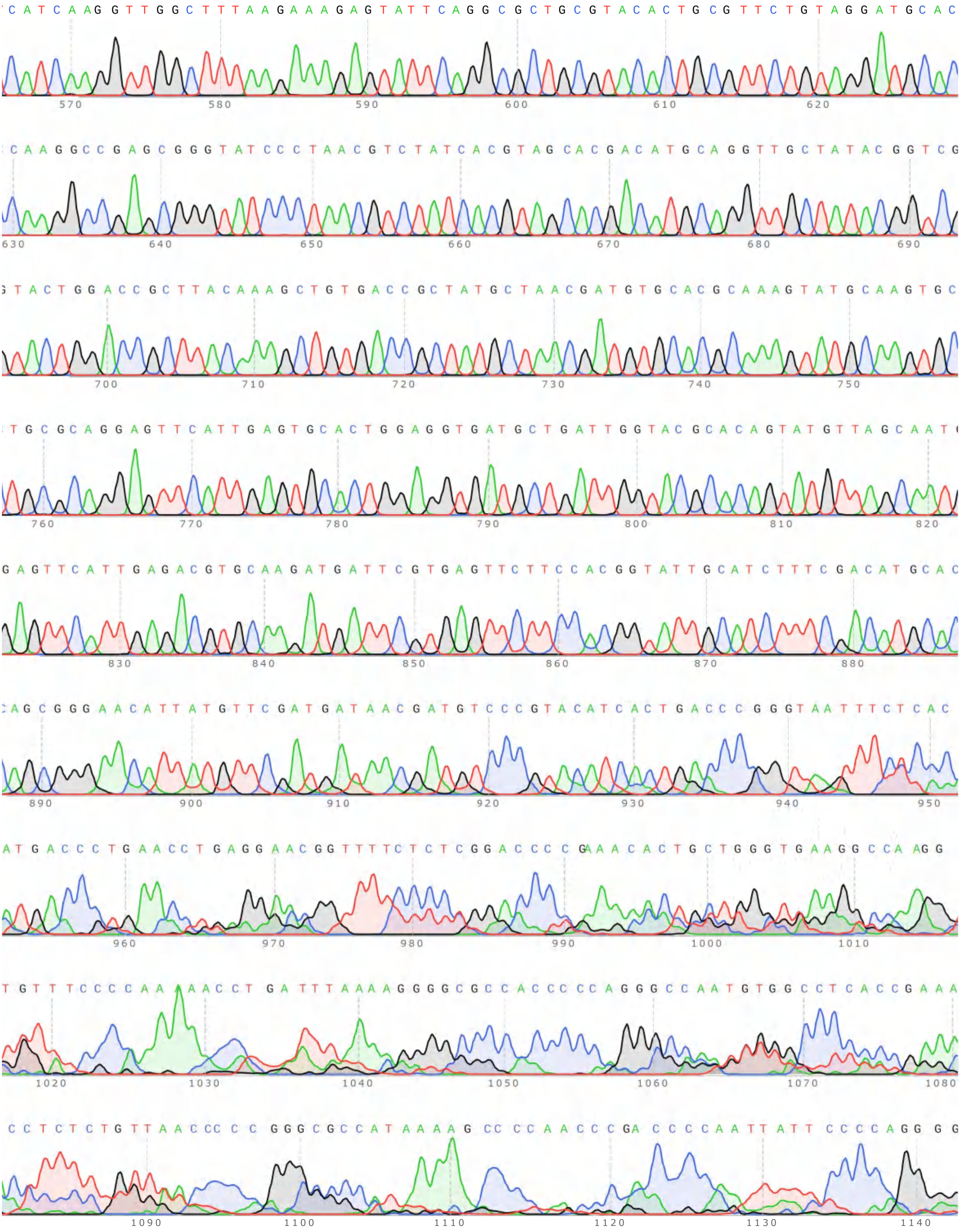
CATCAAGGTTGGCTTTAAGAAAGAGTATTCAGGCGCTGCGTACACTGCGTTCTGTAGGATGCAC
570 580 590 600 610 620
CAAGGCCGAGCGGGTATCCCTAACGTCTATCACGTAGCACGACATGCAGGTTGCTATACGGTCG
630 640 650 660 670 680 690
TACTGGACCGCTTACAAAGCTGTGACCGCTATGCTAACGATGTGCACGCAAAGTATGCAAGTGC
700 710 720 730 740 750
TGCGCAGGAGTTCATTGAGTGCACTGGAGGTGATGCTGATTGGTACGCACAGTATGTTAGCAAT
760 770 780 790 800 810 820
GAGTTCATTGAGACGTGCAAGATGATTCGTGAGTTCTTCCACGGTATTGCATCTTTCGACATGCAC
830 840 850 860 870 880
AGCGGGAACATTATGTTCGATGATAACGATGTCCCGTACATCACTGACCCGGGTAATTTCTCAC
890 900 910 920 930 940 950
ATGACCCTGAACCTGAGGAACGGTTTTCTCTCGGACCCCGAAACACTGCTGGGTGAAGGCCAAGG
960 970 980 990 1000 1010
TGTTTCCCCAAAAACCTGATTTAAAAGGGGCGCCACCCCCAGGGCCAATGTGGCCTCACCGAAA
1020 1030 1040 1050 1060 1070 1080
CCTCTCTGTTAACCCCCGGGCGCCATAAAAGCCCCAACCCGACCCCAATTATTCCCCAGGGG
1090 1100 1110 1120 1130 1140

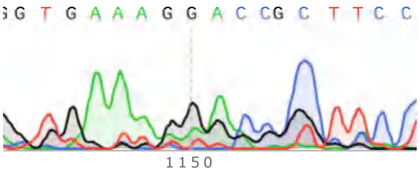
G T G A A A G G A C CG C T T C C
1150

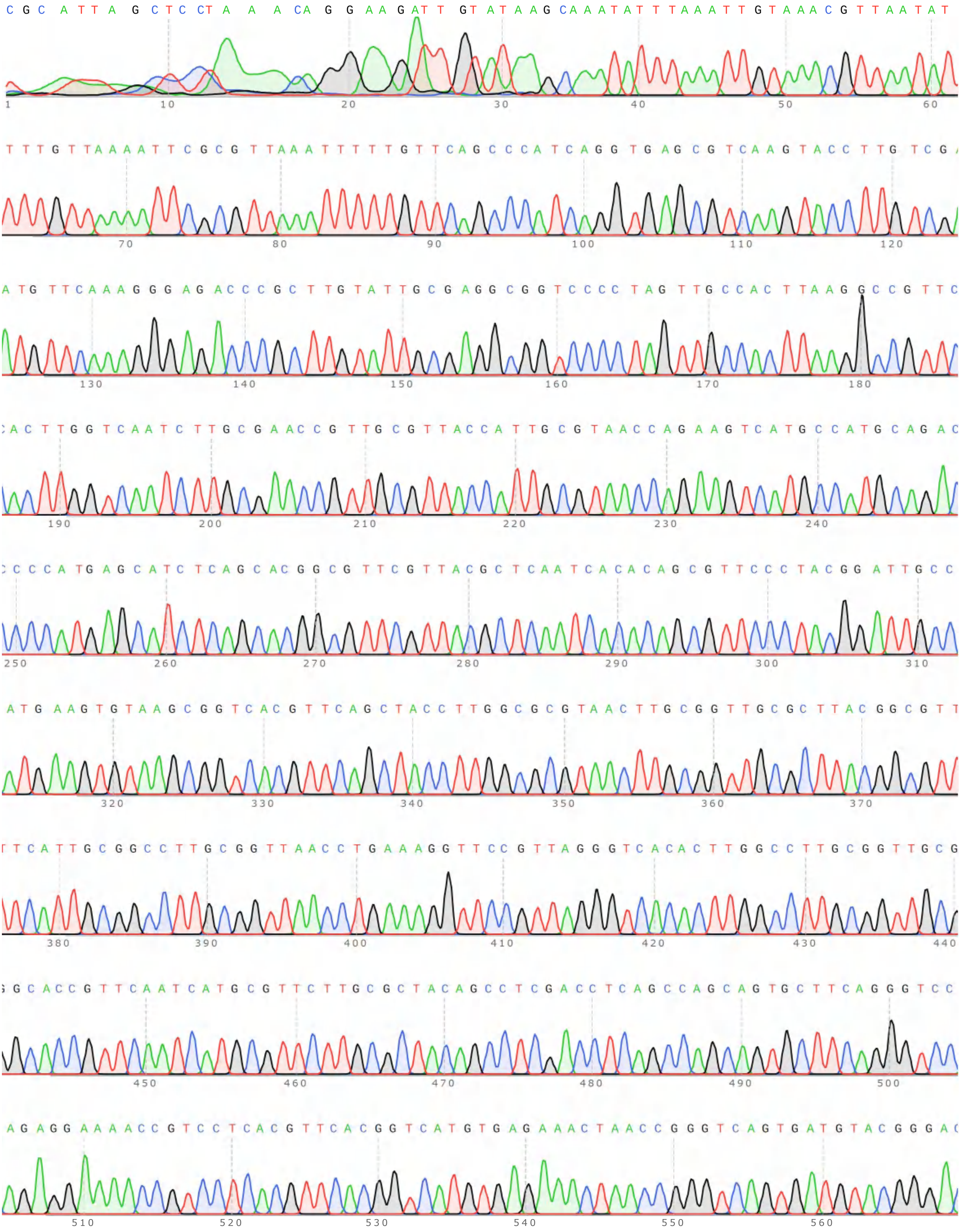
CGCATTAGCTCCTAAACAGGAAGATTGTATAAGCAAATATTTAAATTGTAAACGTTAATAT
1 10 20 30 40 50 60
TTTGTTAAAATTCGCGTTAAATTTTTGTTCAGCCCATCAGGTGAGCGTCAAGTACCTTGTCG
70 80 90 100 110 120
ATGTTCAAAGGGAGACCCGCTTGTATTGCGAGGCGGTCCCCTAGTTGCCACTTAAGGCCGTTC
130 140 150 160 170 180
CACTTGGTCAATCTTGCGAACCGTTGCGTTACCATTGCGTAACCAGAAGTCATGCCATGCAGAC
190 200 210 220 230 240
CCCCATGAGCATCTCAGCACGGCGTTCGTTACGCTCAATCACACAGCGTTCCCTACGGATTGCC
250 260 270 280 290 300 310
ATGAAGTGTAAGCGGTCACGTTCAGCTACCTTGGCGCGTAACTTGCGGTTGCGCTTACGGCGTT
320 330 340 350 360 370
TTCATTGCGGCCTTGCGGTTAACCTGAAAGGTTCCGTTAGGGTCACACTTGGCCTTGCGGTTGCG
380 390 400 410 420 430 440
GGCACCGTTCAATCATGCGTTCTTGCGCTACAGCCTCGACCTCAGCCAGCAGTGCTTCAGGGTCC
450 460 470 480 490 500
AGAGGAAAACCGTCCTCACGTTCACGGTCATGTGAGAAACTAACCGGGTCAGTGATGTACGGGAC
510 520 530 540 550 560

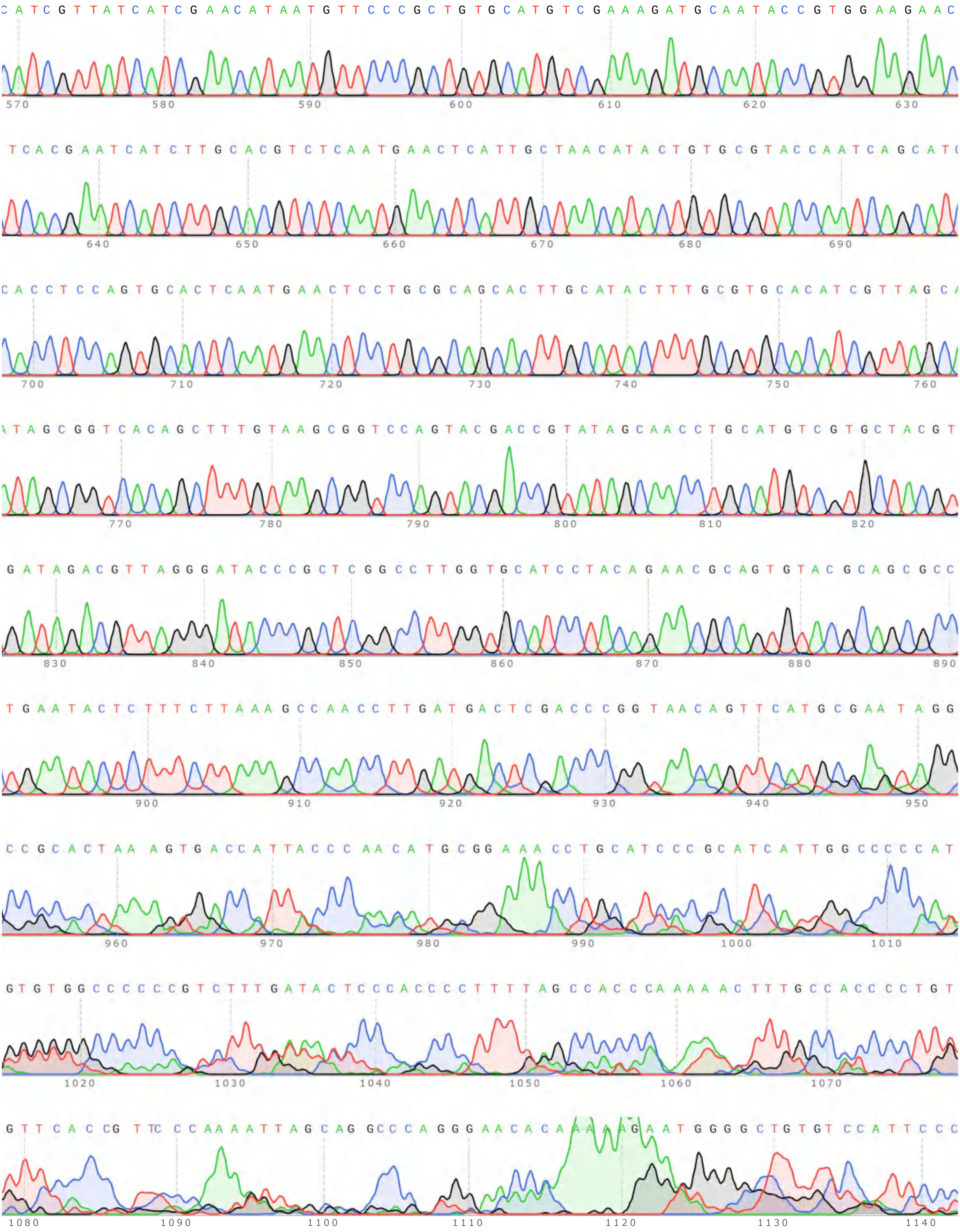
C A T C G T T A T C A T C G A A C A T A A T G T T C C C G C T G T G C A T G T C G A A A G A T G C A A T A C C G T G G A A G A A C
570 580 590 600 610 620 630
T C A C G A A T C A T C T T G C A C G T C T C A A T G A A C T C A T T G C T A A C A T A C T G T G C G T A C C A A T C A G C A T
640 650 660 670 680 690
C A C C T C C A G T G C A C T C A A T G A A C T C C T G C G C A G C A C T T G C A T A C T T T G C G T G C A C A T C G T T A G C A
700 710 720 730 740 750 760
A T A G C G G T C A C A G C T T T G T A A G C G G T C C A G T A C G A C C G T A T A G C A A C C T G C A T G T C G T G C T A C G T
770 780 790 800 810 820
G A T A G A C G T T A G G G A T A C C C G C T C G G C C T T G G T G C A T C C T A C A G A A C G C A G T G T A C G C A G C G C C
830 840 850 860 870 880 890
T G A A T A C T C T T T C T T A A A G C C A A C C T T G A T G A C T C G A C C C G G T A A C A G T T C A T G C G A A T A G G
900 910 920 930 940 950
C C G C A C T A A A G T G A C C A T T A C C C A A C A T G C G G A A A C C T G C A T C C C G C A T C A T T G G C C C C C A T
960 970 980 990 1000 1010
G T G T G G C C C C C C G T C T T T G A T A C T C C C A C C C C T T T T A G C C A C C C A A A A A C T T T G C C A C C C C T G T
1020 1030 1040 1050 1060 1070
G T T C A C C G T T C C C A A A A T T A G C A G G C C C A G G G A A C A C A A A A G A A T G G G G C T G T G T C C A T T C C C
1080 1090 1100 1110 1120 1130 1140

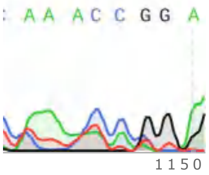
A A A C C G G A
1 1 5 0

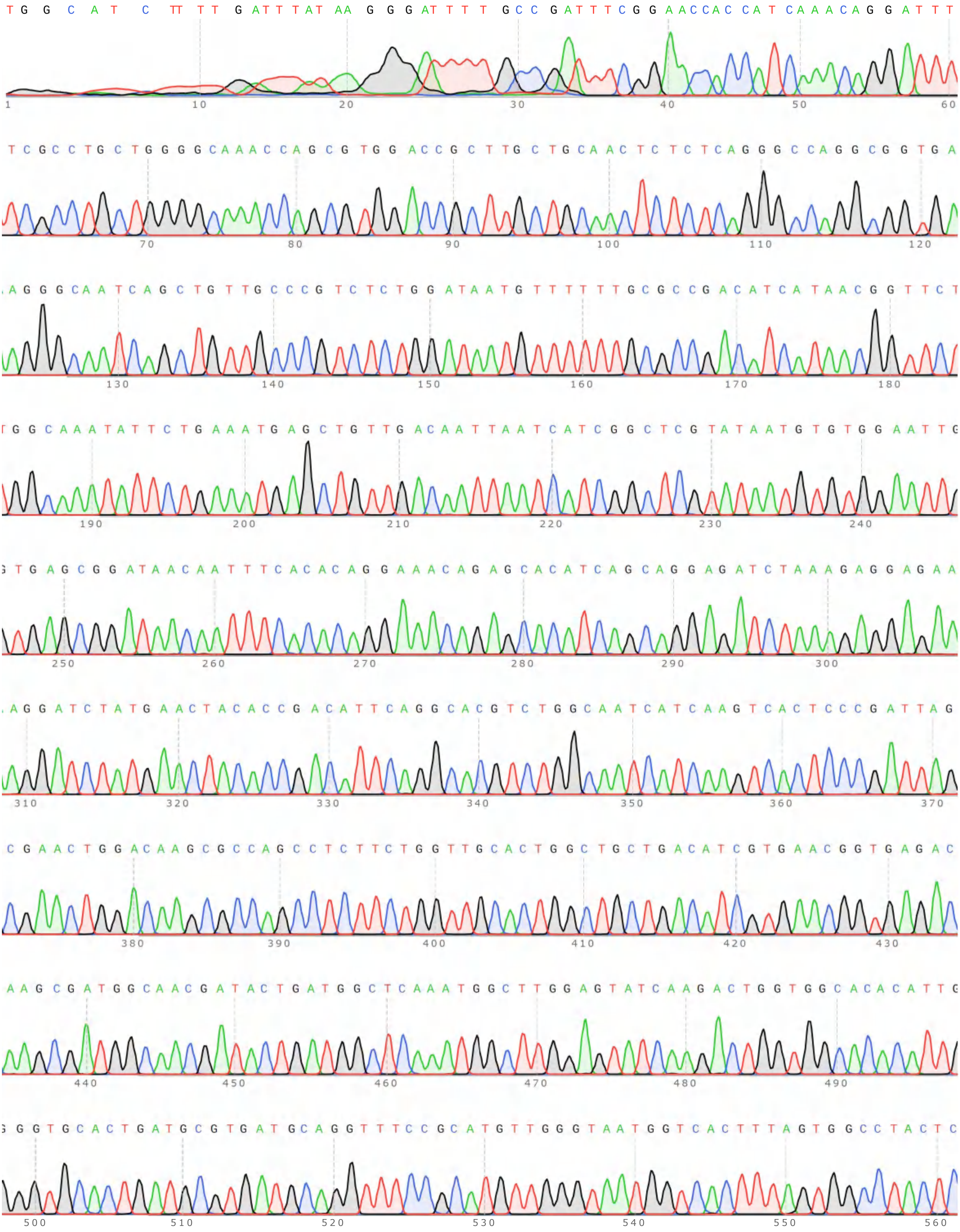
T G G C A T C TT T T G A T T T A T A A G G G A T T T T G C C G A T T T C G G A A C C A C C A T C A A A C A G G A T T T
1 10 20 30 40 50 60
T C G C C T G C T G G G G C A A A C C A G C G T G G A C C G C T T G C T G C A A C T C T C T C A G G G C C A G G C G G T G A
70 80 90 100 110 120
A G G G C A A T C A G C T G T T G C C C G T C T C T G G A T A A T G T T T T T T G C G C C G A C A T C A T A A C G G T T C T
130 140 150 160 170 180
T G G C A A A T A T T C T G A A A T G A G C T G T T G A C A A T T A A T C A T C G G C T C G T A T A A T G T G T G G A A T T G
190 200 210 220 230 240
G T G A G C G G A T A A C A A T T T C A C A C A G G A A A C A G A G C A C A T C A G C A G G A G A T C T A A A G A G G A G A A
250 260 270 280 290 300
A G G A T C T A T G A A C T A C A C C G A C A T T C A G G C A C G T C T G G C A A T C A T C A A G T C A C T C C C G A T T A G
310 320 330 340 350 360 370
C G A A C T G G A C A A G C G C C A G C C T C T T C T G G T T G C A C T G G C T G C T G A C A T C G T G A A C G G T G A G A C
380 390 400 410 420 430
A A G C G A T G G C A A C G A T A C T G A T G G C T C A A A T G G C T T G G A G T A T C A A G A C T G G T G G C A C A C A T T G
440 450 460 470 480 490
G G G T G C A C T G A T G C G T G A T G C A G G T T T C C G C A T G T T G G G T A A T G G T C A C T T T A G T G G C C T A C T C
500 510 520 530 540 550 560

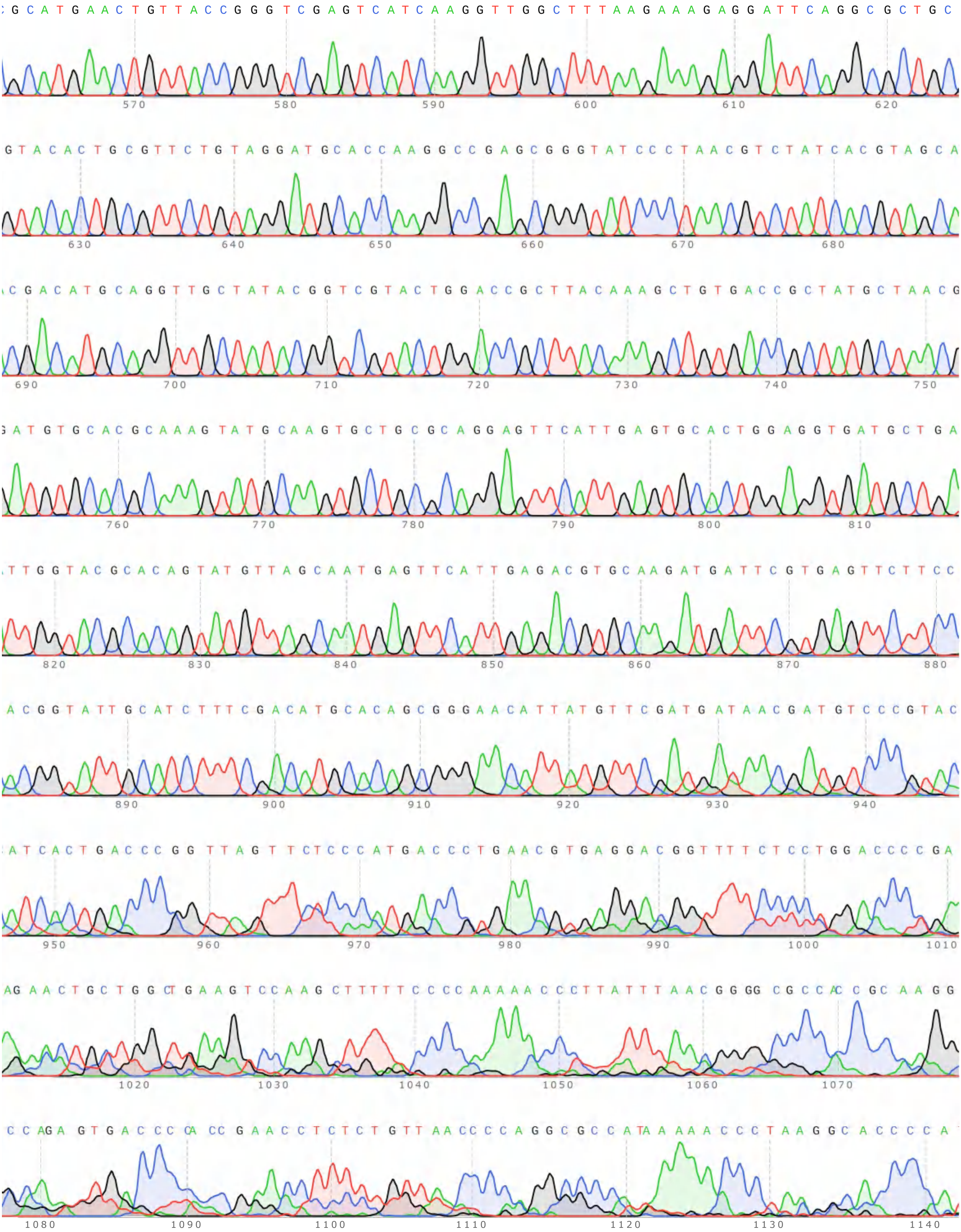
G C A T G A A C T G T T A C C G G G T C G A G T C A T C A A G G T T G G C T T T A A G A A A G A G G A T T C A G G C G C T G C
570 580 590 600 610 620
G T A C A C T G C G T T C T G T A G G A T G C A C C A A G G C C G A G C G G G T A T C C C T A A C G T C T A T C A C G T A G C A
630 640 650 660 670 680
C G A C A T G C A G G T T G C T A T A C G G T C G T A C T G G A C C G C T T A C A A A G C T G T G A C C G C T A T G C T A A C G
690 700 710 720 730 740 750
A T G T G C A C G C A A A G T A T G C A A G T G C T G C G C A G G A G T T C A T T G A G T G C A C T G G A G G T G A T G C T G A
760 770 780 790 800 810
T T G G T A C G C A C A G T A T G T T A G C A A T G A G T T C A T T G A G A C G T G C A A G A T G A T T C G T G A G T T C T T C C
820 830 840 850 860 870 880
A C G G T A T T G C A T C T T T C G A C A T G C A C A G C G G G A A C A T T A T G T T C G A T G A T A A C G A T G T C C C G T A C
890 900 910 920 930 940
A T C A C T G A C C C G G T T A G T T C T C C C A T G A C C C T G A A C G T G A G G A C G G T T T T C T C C T G G A C C C C G A
950 960 970 980 990 1000 1010
AG A A C T G C T G G CT G A A G T C C A A G C T T T T T C C C C A A A A A C C C T T A T T T A A C G G GG C G C C AC C GC A A G G
1020 1030 1040 1050 1060 1070
C C AGA G T G A C C C CA CC G A A C C T C T C T G T T AA CC C C A G G C G C C ATA A A A A C C C T A A G G C A C C C C A
1080 1090 1100 1110 1120 1130 1140

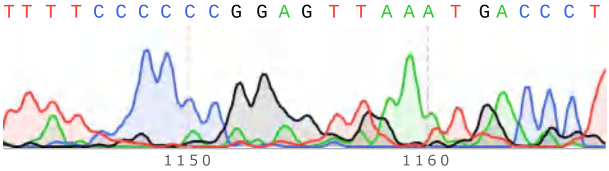
T T T T C C C C C C G G A G T T A A A T G A C C C T
1150
1160

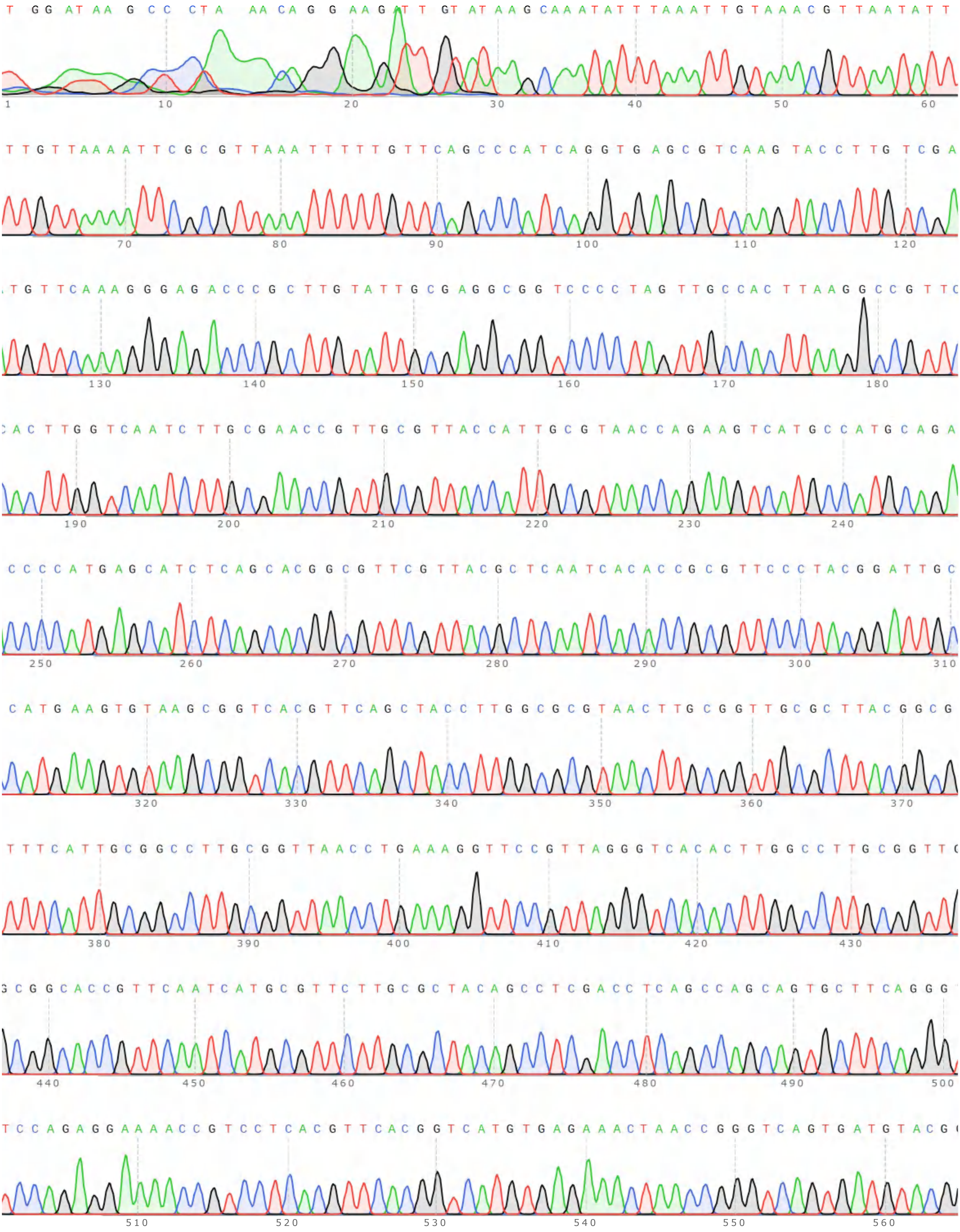
T GG AT AA G C C CT A AA CA G G AA G AT T GT AT A A G C A A A T A T T T A A A T T G T A A A C G T T A A T A T T
T T G T T A A A A T T C G C G T T A A A T T T T T G T T C A G C C C A T C A G G T G A G C G T C A A G T A C C T T G T C G A
T G T T C A A A G G G A G A C C C G C T T G T A T T G C G A G G C G G T C C C C T A G T T G C C A C T T A A G G C C G T T C
A C T T G G T C A A T C T T G C G A A C C G T T G C G T T A C C A T T G C G T A A C C A G A A G T C A T G C C A T G C A G A
C C C C A T G A G C A T C T C A G C A C G G C G T T C G T T A C G C T C A A T C A C A C C G C G T T C C C T A C G G A T T G C
C A T G A A G T G T A A G C G G T C A C G T T C A G C T A C C T T G G C G C G T A A C T T G C G G T T G C G C T T A C G G C G
T T T C A T T G C G G C C T T G C G G T T A A C C T G A A A G G T T C C G T T A G G G T C A C A C T T G G C C T T G C G G T T
G C G G C A C C G T T C A A T C A T G C G T T C T T G C G C T A C A G C C T C G A C C T C A G C C A G C A G T G C T T C A G G G
T C C A G A G G A A A A C C G T C C T C A C G T T C A C G G T C A T G T G A G A A A C T A A C C G G G T C A G T G A T G T A C G

GGACATCGTTATCATCGAACATAATGTTCCCGCTGTGCATGTCGAAAGATGCAATACCGTGGAA

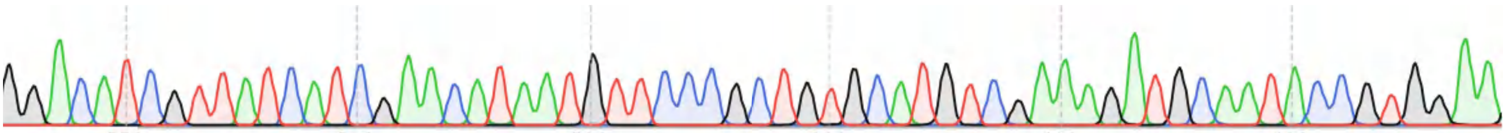

GAACTCACGAATCATCTTGCACGTCTCAATGAACTCATTGCTAACATACTGTGCGTACCAATCA

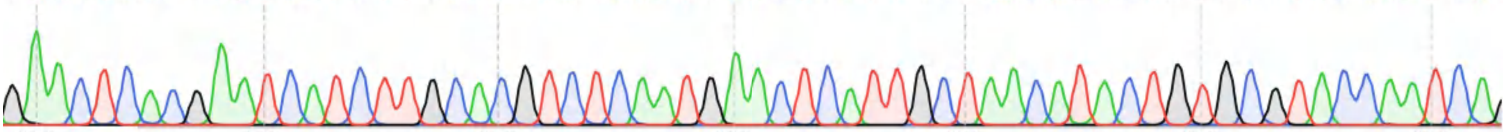

GCATCACCTCCAGTGCACTCAATGAACTCCTGCGCAGCACTTGCATACTTTGCGTGCACATCGTT

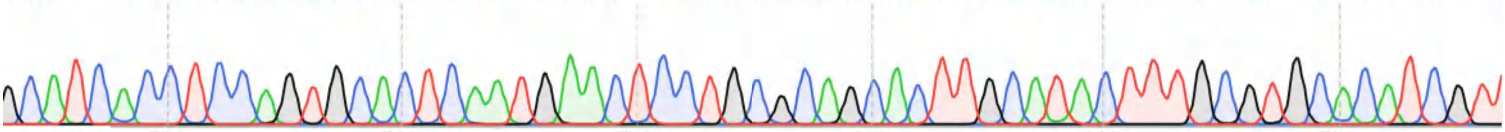

TAGCATAGCGGTCACAGCTTTGTAAGCGGTCCAGTACGACCGTATAGCAACCTGCATGTCGTGCT

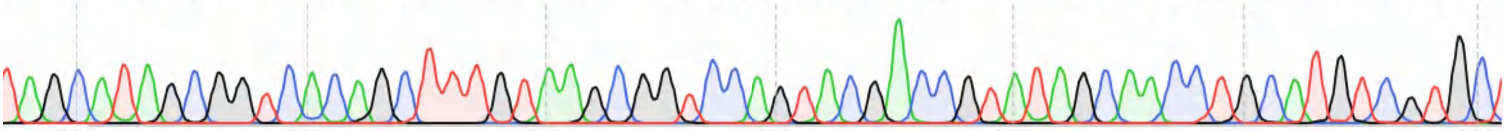

TACGTGATAGACGTTAGGGATACCCGCTCGGCCTTGGTGCATCCTACAGAACGCAGTGTACGCAG

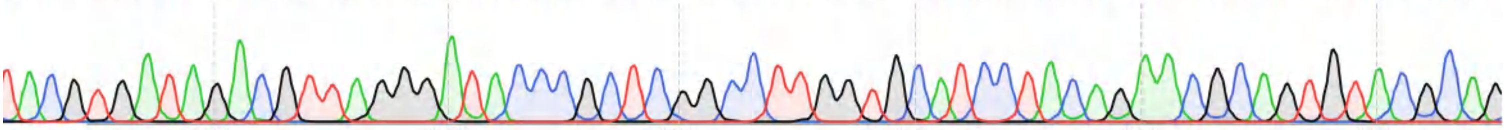

CGCCTGAATCCTCTTTCTTAAAGCCAACCTTGATGACTCGACCCGGTAACAGTTCATGCGAGTA

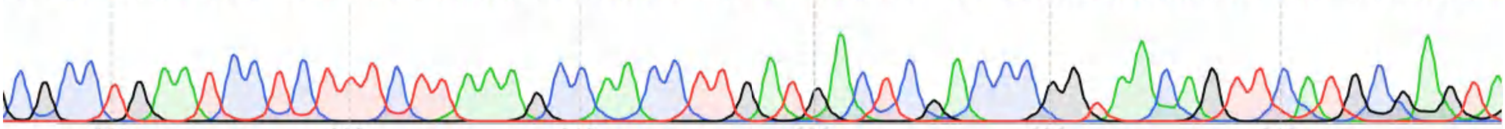

GGCCACTAAAGTGACCATTACCCAACATGCGGAAACCTGCATCACGCATCAGGGCACCCAAGGT

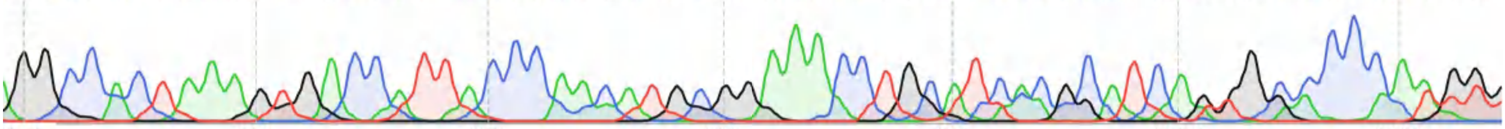

GTGCCACCCGTCTTGATACTCCAACCCTTTTAGCACCCAAATACTTTGCCCTCCCTGTGTCCACC

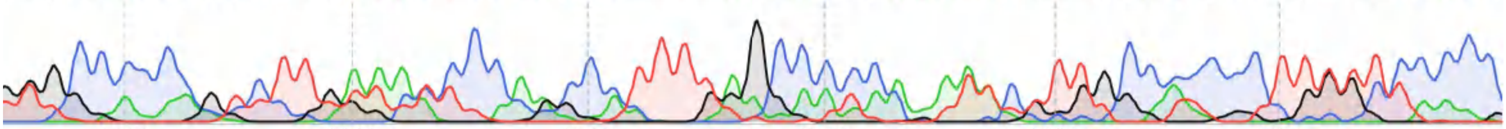

GTCCCAAATTCCCCCCCAGGCACCCAAAAAAGGGGGGCTGTCCATTCCCTACCCGGAGGGC

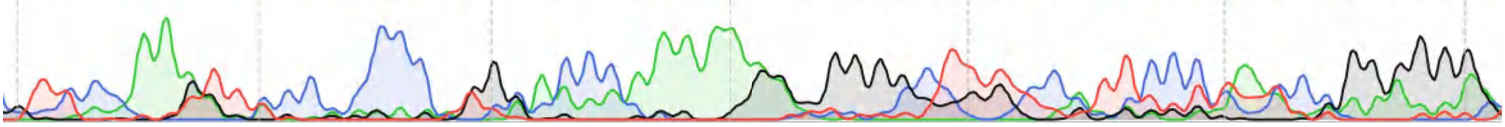

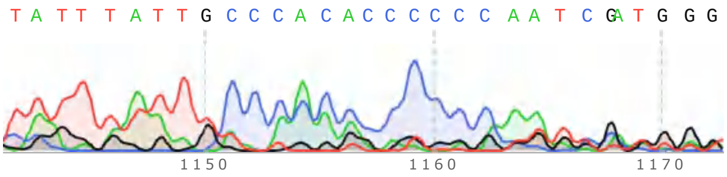

Data Files S2 Sequencing results of recombinant phages after wild-type JSS1_004 was integrated into the M13 genome. Following integration of wild-type JSS1_004 into the M13 genome, propagation was attempted in *E. coli* JM109. Single plaques were isolated for PCR amplification and sequencing, but no M13 recombinants harboring intact wild-type JSS1_004 were detected. Sequencing of putative recombinants revealed pervasive mutations, including missense mutations and premature transcriptional termination caused by base substitutions, as well as frameshift mutations resulting from nucleotide insertions or deletions. The theoretical sequence of M13-KI*004* and representative mutation profiles are provided above, confirming the incompatibility of full-length JSS1_004 with M13 replication despite homologous recombination attempts.
